# Sex- and Age-Related Physiological Profiles for Brachial, Vertebral, Carotid, and Femoral Arteries Blood Flow Velocity Parameters During Growth and Aging (4–76 Years): Comparison With Clinical Cut-Off Levels

**DOI:** 10.3389/fphys.2021.729309

**Published:** 2021-08-26

**Authors:** Yanina Zócalo, Daniel Bia

**Affiliations:** Physiology Department, School of Medicine, CUiiDARTE, Republic University, Montevideo, Uruguay

**Keywords:** adolescents, adults, blood flow velocity, brachial, carotid, children, femoral, physiological aging

## Abstract

Ultrasound-derived blood flow velocity (BFV) levels [e.g., peak systolic velocity (PSV)], intrabeat indexes (e.g., resistive), and intersegment ratios [e.g., internal/common carotid artery (ICA/CCA) PSV ratio] are assessed to describe cardiovascular physiology and health status (e.g., disease severity evaluation and/or risk stratification). In this respect, fixed cut-off values (disregard of age or sex) have been proposed to define “significant” vascular disease from BFV-derived data (parameters). However, the use of single fixed cut-off values has limitations. Accurate use of BFV-derived parameters requires knowing their physiological age-related profiles and the expected values for a specific subject. To our knowledge, there are no studies that have characterized BFV profiles in large populations taking into account: (i) data from different age-stages (as a continuous) and transitions (childhood–adolescence–adulthood), (ii) complementary parameters, (iii) data from different arteries, and (iv) potential sex- and hemibody-related differences. Furthermore, (v) there is little information regarding normative data [reference intervals (RIs)] for BFV indexes.

**Aims:** The aims of this study are the following: (a) to determine the need for age-, body side-, and sex-specific profiles for BFV levels and derived parameters (intrabeat indexes and intersegment ratios), and (b) to define RIs for BFV levels and parameters, obtained from CCA, ICA, external carotid, vertebral, femoral, and brachial arteries records.

**Methods:** A total of 3,619 subjects (3–90 years) were included; 1,152 were healthy (without cardiovascular disease and atheroma plaques) and non-exposed to cardiovascular risk factors. BFV data were acquired. The agreement between left and right data was analyzed (Concordance correlation, Bland–Altman). Mean and SD equations and age-related profiles were obtained for BFV levels and parameters (regression methods; fractional polynomials).

**Results:** Left and right body-side derived data were not always equivalent. The need for sex-specific RIs was dependent on the parameter and/or age considered. RIs were defined for each studied artery and parameter. Percentile curves were compared with recommended fixed cut-off points. The equations for sex, body-side, and age-specific BFV physiological profiles obtained in the large population (of children, adolescents, and adults) studied were included (spreadsheet formats), enabling to determine for a particular subject, the expected values and potential data deviations.

## Introduction

B-mode ultrasound (US) evaluation of peripheral arteries (e.g., carotid and femoral) morpho-structural characteristics (e.g., atherosclerotic plaques presence, diameters, and intima-media thickness) has been proposed as a valuable strategy to assess vascular health status and to improve risk stratification and/or disease diagnose in asymptomatic and healthy subjects. Data from US-derived vascular evaluation has also contributed to enhancing cardiovascular physiology and disease knowledge (e.g., expected vascular changes in relation to growth and aging; Naghavi et al., 2006; Sosnowski et al., 2007; Laclaustra et al., 2016; Marin et al., 2020). Structural assessment can be complemented by Doppler US evaluation, which allows quantifying blood flow velocity (BFV) levels and indexes that give data about micro- and macro-vascular status, and have shown to vary in association with physiological and pathological states (Hwang, 2017).

Using software integrated into conventional US devices, different BFV parameters can be evaluated: (i) BFV levels [e.g., peak systolic (PSV), end-diastolic velocity (EDV)], (ii) “intra-beat” indexes [e.g., resistive (RI), pulsate (PI)], and (iii) “inter-segment” velocity ratios. These complementary parameters are clinically important to assess the functional status of the macro and microvascular systems. As an example, low common carotid artery (CCA) PSV, and EDV are independently associated with cardiovascular disease (CVD) and events (e.g., stroke, heart disease) (Chuang et al., 2011, 2016); high internal carotid artery (ICA) PSV contributes to predict and grade ICA stenosis (Tokunaga et al., 2016); and low common femoral artery (CFA) PSV is predictive of ipsilateral iliac occlusion (Shaalan et al., 2003). On the other hand, CCA PI, and RI are associated with the prevalence of cerebral atherosclerosis and cardiovascular events (e.g., ischemic stroke) (Hitsumoto, 2019), ICA PI is associated with the global burden of small vessels disease (Lau et al., 2018), and CFA PI contributes to detecting peripheral vascular disease (Clifford et al., 1981). In turn, ICA/CCA velocity ratios contribute to detect and define arterial stenosis (Kochanowicz et al., 2009).

Classically US-derived BFV indexes have been used in adults in whom the presence of an arterial alteration is suspected. In this context, single fixed cut-off points (without discriminating by age, hemibody, and/or sex) have been considered, mainly aiming at defining the significance (degree) of a stenosis (e.g., ICA PSV ≥125 cm/s or ICA PSV/CCA PSV ≥2 indicate arterial stenosis <50%) (Oates et al., 2009; Hodgkiss-Harlow and Bandyk, 2013; Freire et al., 2015; Santos et al., 2019). However, changes in BFV indexes could be found long before cut-off values (e.g., indicating significant stenosis) are reached. Indeed, early alterations in microvascular and macrovascular structures or functions (e.g., mimicking the effects of aging) can appear at any stage of life (even in childhood), reflecting a dissociation between chronologic and biologic vascular age and indicating a relative increase in cardiovascular risk (CVR) (Jani and Rajkumar, 2006; Bruno et al., 2020). Consequently, accurate use of BFV parameters in both clinical and cardiovascular research requires knowing the expected (physiological) age-related profiles and the predicted value for a specific subject. However, to our knowledge, there are no studies that have characterized BFV profiles in large populations: (i) considering several complementary indexes, (ii) analyzing data from different arteries, and taking into account potential (iii) differences between left and right hemibodies and/or (iv) sex-related differences, considering “age × sex” interaction (moderation). What is more important, in our understanding, is that no works are assessing BFV variations (as a continuous) considering data from different age stages and their transitions (childhood–adolescence–adulthood). It is to note that studies that aimed at analyzing age-related differences did not allow for their adequate and comprehensive characterization because of the following reasons: (i) considered small numbers of subjects [e.g., *n* = 50 (Seidel et al., 1999; Kuhl et al., 2000), *n* = 78 (Scheel et al., 2000), *n* = 92 (Zbornikova and Lassvik, 1986)], (ii) did not exclude subjects with vascular disease and/or exposed to factors known to impact on the vascular system (Kochanowicz et al., 2009), and/or (iii) compared “mean values” of defined groups considering wide age ranges [e.g., 10 (Zbornikova and Lassvik, 1986)], 20 (Scheel et al., 2000; Albayrak et al., 2007; Nemati et al., 2009), or 30 (Yazici et al., 2005) years (difference in age of subjects in the same group).

Currently, it is unknown whether BFV parameters (i) provide equivalent data when obtained from left or right hemibodies and (ii) levels depend on the age and/or sex of the subjects. Furthermore, there is little information regarding physiological profiles and reference intervals (RIs) for BFV parameters obtained in a large healthy population including children, adolescents, and adults. The aims of this study were the following: (i) to evaluate the agreement between BFV parameters obtained from both hemibodies, (ii) to determine the need for age and/or sex-specific RIs, (iii) to define age-related physiological profiles and RIs for BFV parameters obtained from the assessment of CCA, ICA, external carotid artery (ECA), vertebral artery (VA), CFA, and brachial artery (BA) in a cohort of healthy children, adolescents, and adults.

## Materials and Methods

### Study Population

The work was carried out in the context of the Centro Universitario de Investigación, Innovación y Diagnóstico Arterial (CUiiDARTE) project (Bia et al., 2011; Santana et al., 2012a,b; Zócalo et al., 2017; Bia and Zócalo, 2021; Zócalo and Bia, 2021), a population-based work developed in Uruguay. In this study, we considered data from 3,619 subjects included in the CUiiDARTE Database. This contains data on demographic and anthropometric variables, exposure to cardiovascular risk factors (CRFs), personal and family history of CVD, and data on hemodynamic, structural, and functional vascular parameters (Bia et al., 2011; Santana et al., 2012a,b; Zócalo and Bia, 2016, 2021; Zócalo et al., 2017; Castro et al., 2019; Bia and Zócalo, 2021). All procedures agree with the Declaration of Helsinki (1975 and reviewed in 1983). The work protocol was reviewed and approved by the Ethics Committee of Centro Hospitalario Pereira Rossell, Universidad de la República. The participants provided their written informed consent to participate in this study. In adults, written informed consent was obtained prior to the evaluation. In subjects <18 years, written consent of the parents and assent of the children were obtained before the work. Subjects or parents (in case of subjects aged <18 years) provided informed written consent to have data from their medical records used in research.

### Anthropometric and Clinical Evaluation

A brief clinical and anthropometric evaluation enabled us to assess CRFs. A family history of CVD was defined by the presence of first-degree (all the subjects) and/or (for subjects ≤ 18 years) second-degree relatives with early (<55 years in men; <65 years in women) CVD. Body weight (BW) and height (BH) were measured with the subject wearing light clothing and no shoes. Standing BH was measured using a portable stadiometer and recorded to the nearest 0.1 cm. BW was measured with an electronic scale (841/843, Seca Inc., Hamburg, Germany; model HBF-514C, Omron Inc., Chicago, Illinois, USA) and recorded to the nearest 0.1 kg. Body mass index (BMI) was calculated as BW-to-squared BH ratio. In children and adolescents, BMI *z*-scores (*z*-BMI) were calculated using software of WHO (Anthro-v.3.2.2; Anthro-Plus-v.1.0.4) (Castro et al., 2019).

### Cardiovascular Evaluation

Participants were asked to avoid exercise, tobacco, alcohol, caffeine, and food-intake 4 h before the evaluation. All records were performed in a temperature-controlled room (21–23°C), with the subject in the supine position and after resting for at least 10–15 min, which enabled reaching steady hemodynamic states. Using an oscillometric device (HEM-433INT; Omron Healthcare Inc., Lake Forest, Illinois, USA), heart rate and brachial systolic and diastolic blood pressure (bSBP, bDBP) were recorded simultaneously and/or immediately before or after each US record. Then, brachial pulse pressure (bPP = bSBP–bDBP) and mean pressure (bMBP = bDBP+bPP/3) were obtained.

Cardiovascular evaluation in CUiiDARTE includes assessing the following: (i) peripheral (brachial, radial, and ankle) and central (aortic and carotid) blood pressure (BP) levels, central (aortic and carotid) pulse wave analysis and wave separation analysis-derived parameters (e.g., augmentation index, forward and backward pressure components); (ii) CCA, CFA, and BA beat-to-beat diameter waves and intima-media thickness; (iii) BA reactivity (e.g., flow-mediated dilation and low flow-mediated constriction); (iv) carotid, CA, CFA, and BA Doppler-derived BFV profiles and indexes; (v) ankle–brachial index (ABI); (vi) screening for carotid and femoral atherosclerotic plaques; (vii) CCA, CFA, and BA local stiffness (e.g., elastic modulus); (viii) systemic hemodynamic evaluation (e.g., vascular resistances, cardiac output, and index quantified from BA pulse contour analysis and/or cardiography impedance); (ix) regional stiffness (carotid–femoral and carotid–radial pulse wave velocity). In this study, we focused on US/Doppler-derived BFV data.

### Carotid, Vertebral, Femoral, and Brachial Artery US

#### Morpho-Structural Parameters and Ankle-Brachial Index

Left and right CCA, ICA, ECA, VA, CFA, and left BA were examined (B-Mode and Doppler US, 7–13 MHz, linear transducer, M-Turbo, SonoSite Inc., Bothell, WA, USA) (Zócalo and Bia, 2016). Transverse and longitudinal arterial views were obtained to assess the presence of atherosclerotic plaques. Near and far walls were analyzed. Images were obtained from anterior, lateral, and posterior angles. An atherosclerotic plaque was defined as the following (i) focal wall thickening at least 50% greater than the adjacent segment (ii) focal thickening protruding into the lumen at least 0.5 mm, (iii) or an intima-media thickness ≥1.5 mm (Zbornikova and Lassvik, 1986; Marin et al., 2020).

Left and right bSBP and bDBPas well as tibial artery systolic and diastolic pressures (taSBP, taDBP) were obtained (no fixed order) at 5 min intervals (Hem-4030, Omron Inc., Illinois, USA). At least five measurements were obtained from each recording site. Then, ABI, a marker of arterial permeability and central-peripheral BP amplification, was calculated (Zócalo and Bia, 2016):

(1) $$\begin{array}{l} \text{ABI}=\frac{taSBP}{baSBP} \end{array}$$

An ABI <0.9 is conventionally used as a cut-off value to define the peripheral obstructive arterial disease and increased CVR. Right and left ABI values were used as screening to rule out arterial stenosis of at least 50% distal to CFA (Zócalo and Bia, 2016). None of the subjects included in the RIs group had ABI values <0.9.

#### Hemodynamic or BFV-Related Parameters

**Figure 1:**
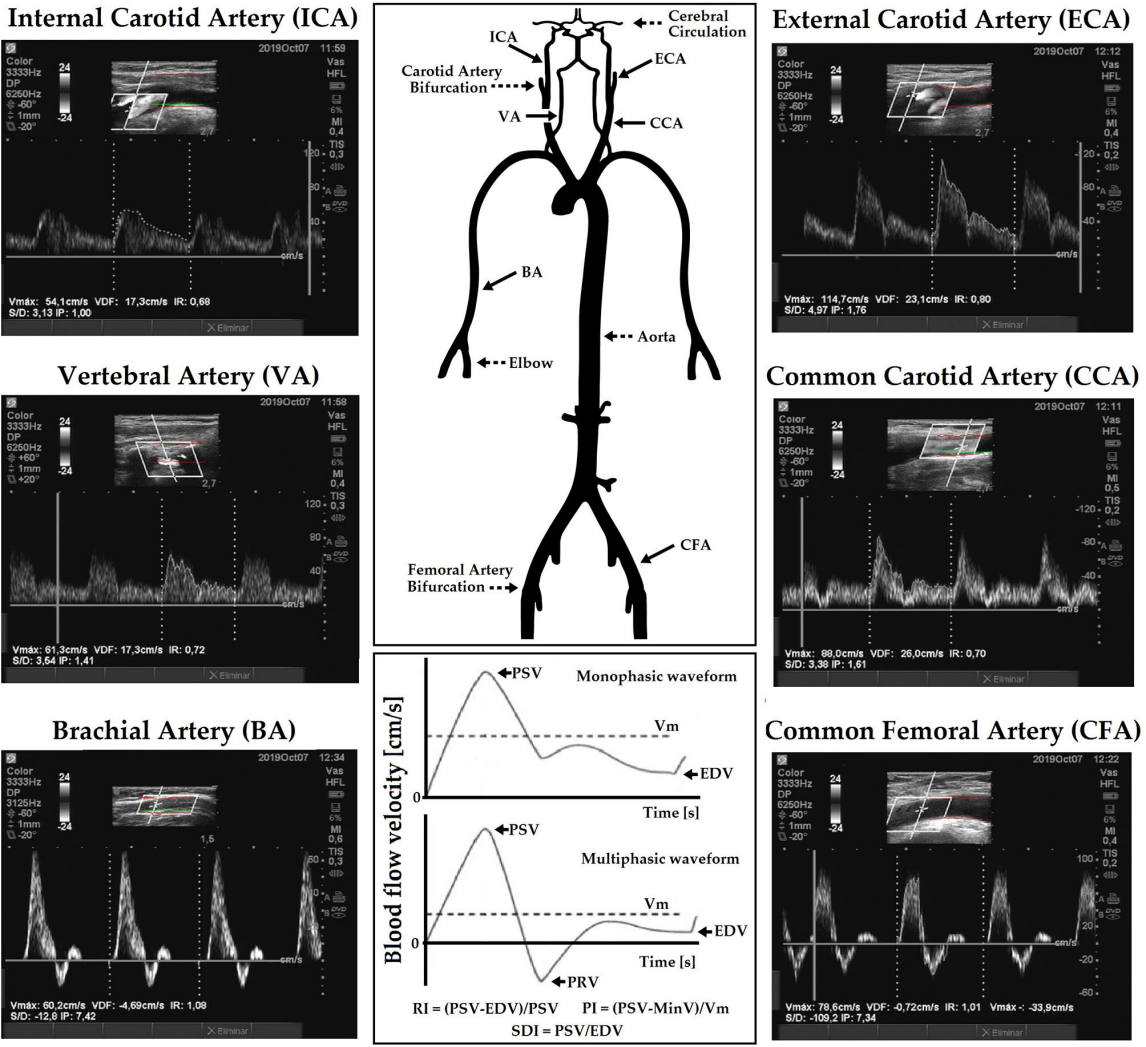
Diagram showing arterial blood flow velocity (BFV) waves recordings and resistive (RI), pulsate (PI), and systo-diastolic index (SDI) calculus, for monophasic and multiphasic waves. Multiphasic waves cross the zero-flow baseline (0 cm/s) and contain both forward and reverse velocity components (e.g., common femoral artery, CFA; brachial artery, BA). Monophasic waves do not cross the zero-flow baseline (e.g., common carotid artery, CCA; Internal carotid artery, ICA) and reflect blood that flows in a single direction during the cardiac cycle. High-resistive waves have a sharp upstroke and a brisk downstroke and may be either multi (e.g., common femoral artery, CFA) or monophasic (e.g., external carotid artery, ECA). Low-resistive waves are monophasic, and contain a prolonged downstroke in the late systole with the continuous forward flow throughout diastole without an end-systolic notch (e.g., ICA). There is a “hybrid” waveform that is monophasic but has features of both high and low resistivity as it contains both brisk downstroke but also continuous forward flow throughout diastole (e.g., CCA).

Common carotid artery, ICA, ECA, VA, CFA, and BA BFVs and patterns were examined by experienced investigators (Doppler US/color flow mapping; 7–13 MHz linear array) (Figure 1). The scan head was moved until the US beam was aligned with the arterial axis so that appropriate longitudinal images were obtained (Gerhard-Herman et al., 2006; Zócalo and Bia, 2016). Measurements were obtained in the longitudinal views, considering arterial segments as long as possible, in which both anterior and posterior walls were visualized (Oates et al., 2009). The insonation angle was always between 30° and 60° (angles > 60° would result in overestimation of the velocity) (Oates et al., 2009). The above enabled to ensure reliable center-line velocity data. Studies lasted for 30–40 min.

Carotid and vertebral evaluations were done with head of the subject rotated away from the side being examined. The sample volume was placed 15–20 mm proximal or distal to CCA bifurcation, defined as the tip of the flow divider, to obtain (right and left) ICA, ECA, or CCA BFV waveforms (Oates et al., 2009). Sample volume was placed below or above the standard site of measurements (e.g., in the ICA) if higher flow acceleration was identified with an aliasing artifact. VA was identified between the transverse processes of the vertebrae at the level of CCA bifurcation (V2 segment) (Kuhl et al., 2000). CFA data were obtained from the proximal straight arterial portion at the groin. Since changes in the BFV profile can occur at the bifurcation, care was taken to avoid it during data recording. BA data were obtained from a straight segment of at least 1 cm (elbow level).

The forward PSV reached during a beat, velocity at the end of the diastolic phase, excluding the notch before the next systole, and mean velocity (Vm) levels were computed from drawing the BFV waveform envelope. Peak reversal diastolic velocity (PRV; reverse, backward or upstream flow toward central aorta) was also computed in the CFA (Figure 1). PSV can vary in association with physiological (e.g., physical activity) or pathological (e.g., stenosis, arterio-venous fistula) conditions. In turn, EDV is usually a faithful mirror of vascular conditions and needs of the downstream territory; its reduction reflects an increase in the distal vascular resistance.

“Intra-beat” indexes, RI, PI, and systo-diastolic index (SDI) (unitless and independent of possible inaccuracies in the estimation of spectral velocity due to arterial diameter or angle correction), were calculated (Figure 1; Oates et al., 2009; Oglat et al., 2018). These indexes provide information about blood flow and resistance that cannot be obtained from measurements of absolute velocity alone (Oates et al., 2009). RI (Pourcelot's index), which reflects global resistance or needs of the downstream territory was calculated as follows:

(2) $$\begin{array}{l} RI\;=\frac{\left(PSV-EDV\right)}{PSV} \end{array}$$

Resistive index reaches its limit in high resistance territories with no end-diastolic blood flow (EDV = 0), and so RI = 1. PI (or Gosling's Index) can be calculated from the equation:

(3) $$\begin{array}{l} PI\;=\frac{\left(PSV-MinV\right)}{Vm} \end{array}$$

The minimal (negative or positive) velocity is denoted as MinV. MinV could be the PRV (e.g., for CFA) or EDV (e.g., for CCA). Vm (or TAMaxV), the time average of maximum velocities, should not be confused with TAMeanV (time average of mean velocities), which corresponds to the mean of the mean velocity envelope curve of a beat and is frequently used for estimation of blood flow volume. PI also reflects the vascular resistance of the downstream territory, even between territories of high resistance. Thus, PI can demonstrate the differences of resistance in territories incompatible with RI (territories where RI = 1). RI or PI and the range of downstream territory perfusion are inversely related; when the resistance increases (RI and PI increase), perfusion would decrease (Oglat et al., 2018). Both indexes are partially determined by vascular stiffness (e.g., higher the stiffness, lesser the wall buffering function, and consequently, higher PI). SDI was quantified as follows:

(4) $$\begin{array}{l} SDI\;=\frac{PSV}{EDV} \end{array}$$

Several potential sources of variability in ICA PSV have been described (e.g., variations in the geometry of the bifurcations or bulb size, presence of collateral flow effects including intracranial/ECA collateral flow, and variations in US device parameters) (Oates et al., 2009). The effects of the described factors on ICA BFV would be mitigated by the use of “inter-segment” velocity ratios or indexes. These would also moderate intermachine (US devices) differences. Two different ratios were calculated: (i) PSV ratio (PSVR) and (ii) St Mary's ratio (SMR):

(5) $$\begin{array}{l} PSVR\;=\frac{ICA\;PSV}{CCA\;PSV} \end{array}$$

(6) $$\begin{array}{l} SMR\;=\frac{ICA\;PSV}{CCA\;EDV} \end{array}$$

Note that SMR is the ratio between a value increasing with the degree of stenosis and a value that decreases with increasing ICA resistance due to progressive stenosis. Table 1 shows indexes obtained from measurement and calculations.

**Table 1:** Blood flow velocity (BFV) and related parameters.

**Blood velocity parameters**	**Intra-segment velocity indexes**	**Inter-segment velocities ratios**
Peak systolic velocity (PSV)	Resistive (RI) = PSV – EDV)/PSV	PSVR = ICA_PSV_/CCA_PSV_
End-diastolic velocity EDV	Pulsate (PI) = PSV – MinV)/Vm	SMR = ICA_PSV_/CCA_EDV_
Peak reversal velocity (PRV)	Systo-Diastolic (SDI) = PSV/EDV	–

*PSVR, Peak Systolic Velocity Ratio; SMR, St Mary Ratio*.

### Data Analysis

**Figure 2:**
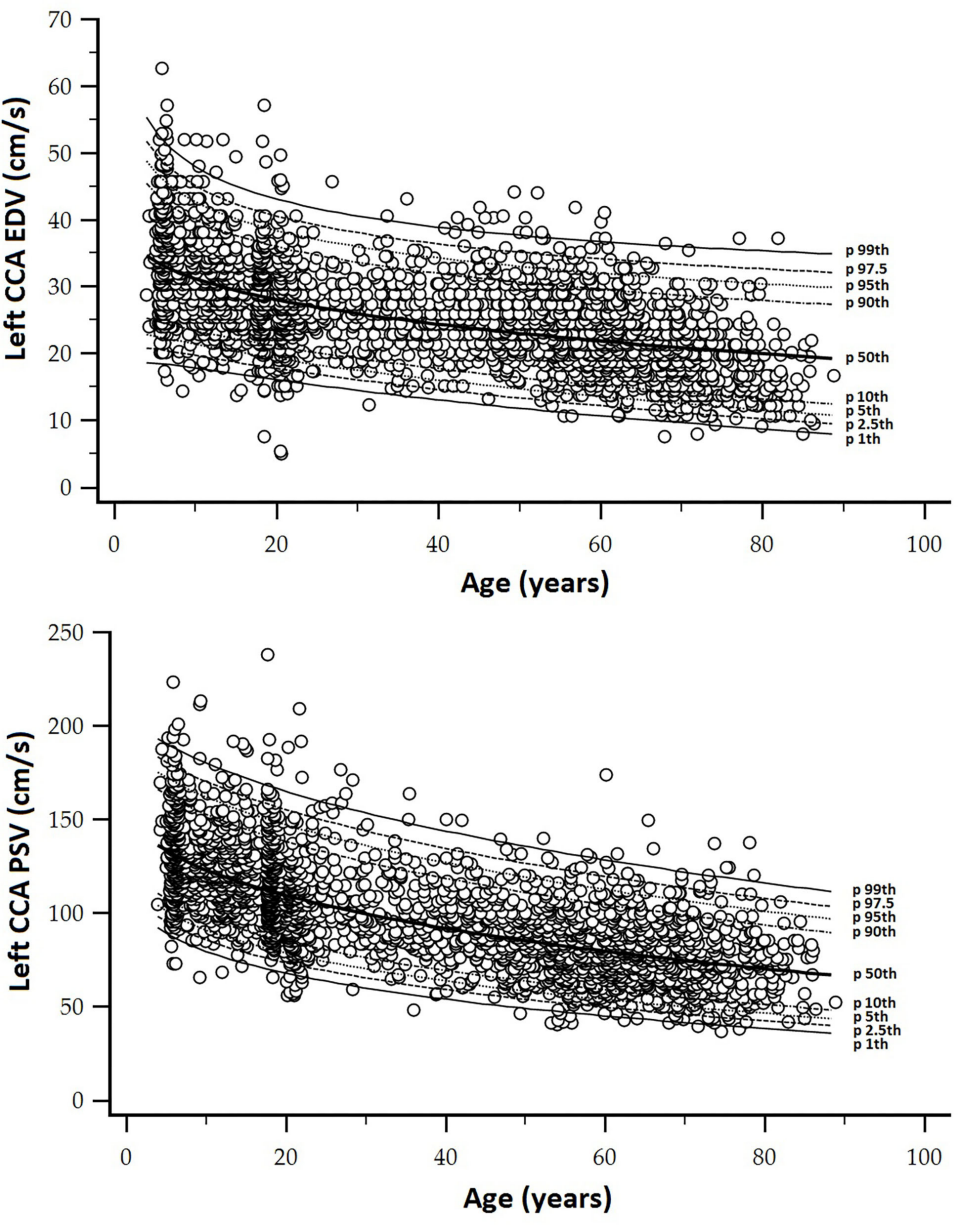
**(A)** Top: Age-related profiles (1st, 2.5th, 5th, 10th, 50th, 90th, 95th, 97.5th, and 99th percentiles) for left CCA end-diastolic velocity (EDV). **(B)** Bottom: Age-related profiles (1st, 2.5th, 5th, 10th, 50th, 90th, 95th, 97.5th, and 99th percentiles) for left CCA peak systolic velocity (PSV).

A step-wise analysis was performed. First, after descriptive statistics were computed and checked (Tables 2, 3; Supplementary Material 1: Tables S1, S2), it was analyzed whether the studied variables showed (in our population) the expected tendency in terms of age-related variations (Figure 2).

**Table 2:** Demographic, anthropometric, and clinical characteristics of subjects.

	**All (** ***n*** **=** **3,619; Age: 2.8–89.0 years)**							**Reference intervals group (** ***n*** **=** **1,152; Age: 2.8–76.5 years)**						
**Variable**	**MV**	**SD**	**Min**	**p25th**	**p50th**	**p75th**	**Max**	**MV**	**SD**	**Min**	**p25th**	**p50th**	**p75th**	**Max**
Age (years)	33.9	24.2	2.8	11.6	24.0	56.5	89.0	17.3	13.5	2.8	6.2	15.1	20.5	76.5
BW (Kg)	61.2	25.3	12.3	45.6	63.2	78.1	150.6	46.1	22.4	12.3	22.1	50.5	64.0	105.0
BH (m)	1.5	0.2	0.9	1.5	1.6	1.7	2.0	1.5	0.3	0.9	1.2	1.6	1.7	1.9
BMI (Kg./m^2^)	24.1	6.0	11.5	19.7	23.7	27.8	71.3	20.0	4.1	11.5	16.5	19.5	23.1	30.0
z-BMI (SD)	0.9	1.5	−4.6	−0.1	0.7	1.8	8.0	0.3	0.9	−4.6	−0.3	0.4	1.0	2.0
TC (mg/dl)	200.2	44.3	94.3	170.0	196.0	227.0	379.0	192.2	26.0	99.0	174.0	194.5	212.0	238.0
HDL (mg/dl)	51.2	15.1	17.0	41.0	49.0	60.0	122.0	57.6	12.2	41.0	49.0	55.0	64.0	100.0
LDL (mg/dl)	123.4	39.8	28.0	95.0	119.0	146.0	323.0	114.7	26.0	31.0	98.0	116.0	132.0	180.0
TG (mg/dl)	133.2	86.0	24.0	80.0	111.0	158.5	783.0	95.8	42.3	24.0	62.0	86.0	120.0	272.0
Glicaemia (mg/dl)	94.4	18.7	40.0	85.0	92.0	100.0	307.0	86.9	9.1	40.0	83.0	87.0	92.0	114.0
bSBP (mmHg)	119.0	16.8	64.3	107.1	118.5	128.8	235.0	111.1	13.0	80.0	100.6	110.4	120.0	171.0
bDBP (mmHg)	68.9	10.4	41.3	60.8	67.7	75.8	129.2	64.1	7.9	46.7	58.6	62.8	69.0	97.4
TC ≥240 mg/dl (%)	7.2							0.0						
HDL <40 mg/dl (%)	8.9							0.0						
Glic ≥126 mg/dl (%)	0.9							0.0						
Current Smoke (%)	11.5							0.0						
Hypertension (%)	26.5							0.0						
Diabetes (%)	5.7							0.0						
History of CVD (%)	8.8							0.0						
Obesity (%)	21.9							0.0						
Familiar CVD (%)	13.5							6.4						
Sedentarism (%)	45.6							31.2						
Anti-HT (%)	21.8							0.0						
Anti-Hyperlip. (%)	13.5							0.0						
Anti-Diabetic (%)	4.1							0.0						
Atheroma Plaq. (%)	22.2							0.0						
Right ABI	1.14	0.08	0.71	1.08	1.14	1.19	1.61	1.14	0.08	0.90	1.08	1.13	1.19	1.61
Left ABI	1.15	0.08	0.68	1.09	1.15	1.20	1.45	1.14	0.08	0.90	1.09	1.14	1.20	1.45

*MV, mean value; SD, standard deviation; Min, minimal; Max, maximal; p25th, p50th, p75th, 25, 50 and 75 percentile; BW, BH, BMI, Body weight, height and mass index; bSBP, bDBP, brachial systolic and diastolic pressure; ABI, ankle-brachial index; CVD, cardiovascular disease; TC, total cholesterol; Familiar CVD, familiar history of premature CVD; Anti-HT, antihypertensive agents; anti-hyperlip, anti-hyperlipidemic agents; anti-diabetic, antidiabetic agents; TG, triglycerides; Glic, Glicaemia; Atheroma Plaq., atherosclerotic plaques*.

**Table 3:** BFV parameters and derived indexes.

	**All (** ***n*** **=** **3,619; Age: 2.8–89.0 years)**							**Reference intervals group (** ***n*** **=** **1,152; Age: 2.8–76.5 years)**						
**Variable**	**MV**	**SD**	**Min**	**p25th**	**p50th**	**p75th**	**Max**	**MV**	**SD**	**Min**	**p25th**	**p50th**	**p75th**	**Max**
**Left common carotid artery**														
PSV (cm/s)	101.2	28.7	36.9	79.9	98.8	120.4	238.0	114.1	25.5	55.8	96.6	111.9	129.4	223.6
EDV (cm/s)	26.5	7.5	5.1	21.3	25.9	31.2	62.7	29.4	7.2	5.1	24.4	28.8	33.3	62.7
RI	0.73	0.06	0.49	0.69	0.73	0.77	1.01	0.74	0.06	0.54	0.70	0.74	0.78	1.01
PI	1.85	0.51	0.22	1.51	1.77	2.11	6.68	1.91	0.51	0.22	1.59	1.82	2.16	6.68
SDI	3.93	1.08	1.85	3.24	3.74	4.43	23.02	4.02	1.16	2.15	3.37	3.82	4.45	23.02
**Left internal carotid artery**														
PSV (cm/s)	81.2	25.8	27.2	62.4	76.1	94.4	217.0	91.3	26.2	35.1	72.6	86.8	106.6	213.9
EDV (cm/s)	30.6	10.4	0.7	23.6	28.9	36.0	87.5	34.6	10.5	9.1	27.4	33.0	40.3	87.5
RI	0.62	0.07	0.37	0.57	0.62	0.67	0.97	0.62	0.07	0.37	0.57	0.61	0.67	0.88
PI	1.15	0.30	0.28	0.95	1.11	1.28	4.15	1.16	0.31	0.28	0.95	1.12	1.30	4.15
SDI	2.75	0.92	1.60	2.34	2.63	3.00	38.06	2.69	0.54	1.60	2.30	2.60	3.00	5.21
**Left external carotid artery**														
PSV (cm/s)	91.0	24.2	34.3	74.0	88.3	105.2	249.9	92.1	23.3	38.1	75.0	89.8	106.0	196.7
EDV (cm/s)	14.3	5.9	0.7	10.7	13.7	17.3	57.1	13.5	5.6	0.8	10.1	12.9	16.0	45.4
RI	0.84	0.06	0.60	0.80	0.84	0.88	1.00	0.85	0.06	0.60	0.81	0.85	0.89	1.00
PI	2.52	0.77	0.15	2.02	2.41	2.86	9.39	2.68	0.79	0.15	2.15	2.55	3.04	7.39
SDI	8.80	13.9	2.53	5.08	6.26	8.17	179.31	10.47	17.44	2.53	5.31	6.61	8.80	165.30
**Left ICA/CCA velocity ratios**														
PSVR	0.83	0.23	0.32	0.68	0.80	0.95	3.00	0.81	0.20	0.32	0.67	0.80	0.93	1.56
SMR	3.20	1.12	1.12	2.53	3.00	3.62	19.04	3.16	0.92	1.30	2.55	3.03	3.59	8.45
**Left vertebral artery**														
PSV (cm/s)	59.4	19.9	19.5	45.4	55.9	70.0	181.5	67.03	21.26	21.30	52.50	63.90	79.30	181.50
EDV (cm/s)	17.2	5.9	3.1	13.0	16.7	20.6	54.1	18.59	6.06	3.81	14.40	17.80	22.80	54.10
RI	0.7	0.1	0.4	0.7	0.7	0.8	1.0	0.71	0.08	0.47	0.66	0.72	0.77	1.01
PI	1.5	0.5	0.6	1.2	1.4	1.7	7.2	1.56	0.47	0.68	1.22	1.50	1.80	4.37
SDI	3.8	3.0	1.5	2.8	3.4	4.2	74.2	3.94	3.25	1.52	2.95	3.57	4.37	70.90
**Right common carotid artery**														
PSV (cm/s)	94.3	27.2	30.8	74.3	91.3	111.8	227.2	106.7	24.23	46.40	90.10	104.60	121.90	210.60
EDV (cm/s)	25.2	7.2	7.2	20.2	24.4	29.7	57.7	28.12	6.95	9.01	23.10	27.40	32.00	56.60
RI	0.7	0.1	0.5	0.7	0.7	0.8	0.9	0.73	0.06	0.52	0.70	0.73	0.77	0.89
PI	1.8	0.5	0.4	1.5	1.8	2.1	4.6	1.92	0.50	0.78	1.58	1.84	2.16	4.61
SDI	3.8	1.0	1.2	3.2	3.7	4.3	10.0	3.90	0.92	1.82	3.28	3.69	4.34	9.51
**Right internal carotid artery**														
PSV (cm/s)	78.2	24.1	24.0	62.4	73.6	88.3	209.3	86.33	24.56	33.20	69.20	82.20	98.60	207.90
EDV (cm/s)	29.3	9.4	1.8	22.8	27.9	34.3	80.5	32.78	9.39	10.70	26.00	31.20	38.10	80.50
RI	0.6	0.1	0.4	0.6	0.6	0.7	0.9	0.61	0.07	0.38	0.57	0.62	0.66	0.88
PI	1.3	4.1	0.4	1.0	1.1	1.3	184.0	1.15	0.27	0.47	0.95	1.11	1.29	2.48
SDI	2.7	0.6	1.6	2.4	2.6	3.0	12.5	2.67	0.49	1.61	2.33	2.60	2.96	5.29
**Right external carotid artery**														
PSV (cm/s)	91.2	25.2	32.8	73.1	88.3	106.0	220.8	92.15	25.05	36.50	73.10	89.95	107.50	195.60
EDV (cm/s)	14.0	5.8	0.1	10.7	13.2	16.7	48.2	12.96	5.49	0.72	9.90	12.65	15.90	40.60
RI	0.8	0.1	0.6	0.8	0.9	0.9	1.0	0.86	0.06	0.66	0.82	0.86	0.89	1.02
PI	2.6	0.8	0.3	2.1	2.5	3.0	8.1	2.83	0.82	0.25	2.28	2.73	3.23	6.45
SDI	9.7	27.9	2.4	5.2	6.5	8.4	1157.0	11.53	19.16	2.91	5.51	6.91	9.00	152.20
**Right ICA/CCA velocity ratios**														
PSVR	0.87	0.24	0.31	0.72	0.84	0.98	3.46	0.82	0.20	0.33	0.69	0.81	0.93	1.74
SMR	3.28	1.12	1.00	2.58	3.06	3.68	14.23	3.13	0.84	1.19	2.56	3.00	3.56	7.34
**Right vertebral artery**														
PSV (cm/s)	53.5	20.6	22.4	41.8	49.8	61.7	487.0	58.28	17.08	22.70	45.40	56.30	67.20	139.40
EDV (cm/s)	15.2	5.3	3.6	11.5	14.8	18.3	38.1	16.17	5.42	3.61	12.30	15.20	19.00	38.10
RI	0.7	0.1	0.4	0.7	0.7	0.8	1.0	0.72	0.09	0.47	0.66	0.72	0.77	1.01
PI	1.5	0.6	0.5	1.2	1.4	1.8	10.0	1.59	0.50	0.69	1.22	1.52	1.84	3.86
SDI	3.8	2.4	1.8	2.9	3.4	4.2	51.1	3.83	1.26	1.76	2.90	3.55	4.43	9.22
**Left common femoral artery**														
PSV (cm/s)	114.5	31.5	39.6	91.3	111.6	134.5	278.5	120.6	30.7	41.1	99.5	118.2	139.4	278.5
PRV (cm/s)	−29.7	12.7	−77.4	−37.3	−30.0	−22.8	40.9	−28.4	14.3	−77.4	−37.3	−28.8	−20.9	38.1
RI	1.0	0.1	0.7	0.9	1.0	1.0	1.6	0.98	0.07	0.73	0.92	1.01	1.01	1.27
PI	7.6	9.7	1.7	4.5	6.2	8.3	206.2	6.59	6.48	1.71	4.00	5.58	7.38	122.20
SDI	78.5	55.0	−10.7	11.1	97.9	122.2	198.3	69.73	58.17	−10.7	10.00	89.05	122.20	192.30
**Right common femoral artery**														
PSV (cm/s)	114.4	31.1	34.3	91.3	110.4	134.5	236.0	120.6	29.91	44.00	99.70	117.70	138.90	224.60
PRV (cm/s)	−30.8	12.6	−80.0	−38.1	−31.0	−24.1	38.5	−30.55	14.38	−80.0	−38.9	−31.30	−23.10	33.00
RI	1.0	0.1	0.2	1.0	1.0	1.0	2.6	0.98	0.10	0.24	0.92	1.01	1.01	2.58
PI	8.0	7.8	1.2	4.7	6.3	8.7	101.1	7.17	6.86	1.89	4.23	5.66	7.71	83.90
SDI	82.5	52.9	−7.5	11.6	99.9	122.2	209.9	75.70	55.78	−7.48	10.50	95.90	123.90	192.20
**Left brachial artery**														
PSV (cm/s)	74.3	22.8	27.8	57.3	70.9	86.8	191.1	83.9	25.2	27.8	67.0	82.2	98.1	191.1
EDV (cm/s)	3.4	8.9	−48.1	0.0	0.0	9.4	52.6	3.6	10.9	−48.1	0.0	1.3	10.8	33.0
RI	1.0	0.1	0.6	0.9	1.0	1.0	1.4	0.94	0.10	0.56	0.88	0.98	1.00	1.41

*MV, mean value; SD, standard deviation; Min., minimum; Max, maximum; p25th, p50th, p75th, 25, 50 and 75 percentiles; PSV, peak systolic velocity; PRV, peak reversal velocity; EDV, end-diastolic velocity; RI, PI, SDI, resistive, pulsate and systo-diastolic index; PSVR, PSV ratio; SMR, St' Mary ratio; ICA, internal carotid artery; CCA, common carotid artery*.

Second, a subgroup of subjects (*n* = 1,688, 864 women) was defined to determine the RIs following European reference values for arterial measurements collaboration group criteria (Engelen et al., 2013, 2015; Bossuyt et al., 2015). This subgroup included subjects who did not have any of the following: (i) CVD; (ii) use of BP-, lipid- and/or glucose-lowering drugs; (iii) arterial hypertension (≥18 years: bSBP ≥ 140 and/or bDBP ≥ 90 mmHg; <18 years: bSBP and bDBP <95th percentile for sex, age, and BH); (iv) smoking; (v) diabetes (self-reported or fasting plasma glucose ≥126 mg/dl); (vi) dyslipidemia (self-reported or total cholesterol ≥240 mg/dl or HDL <40 mg/dl); (vii) obesity (≥18 years: BMI ≥30 kg/m^2^; <18 years: z-BMI ≥2.0). None of them had congenital, chronic, or infectious diseases, or heart rhythm other than sinus rhythm. In this subgroup, we analyzed the association of the presence of carotid and femoral atherosclerotic plaques with BFV indexes (partial correlations; age-adjusted) (Supplementary Material 1: Tables S3, S4). Results showed that the presence of plaques was associated with BFV levels. Then, subjects with plaques were excluded from the RIs subgroup (now called, BFV RIs subgroup; *n* = 1,152, women: 582) (Tables 2, 3; Supplementary Material 1: Tables S1, S2).

**Figure 3:**
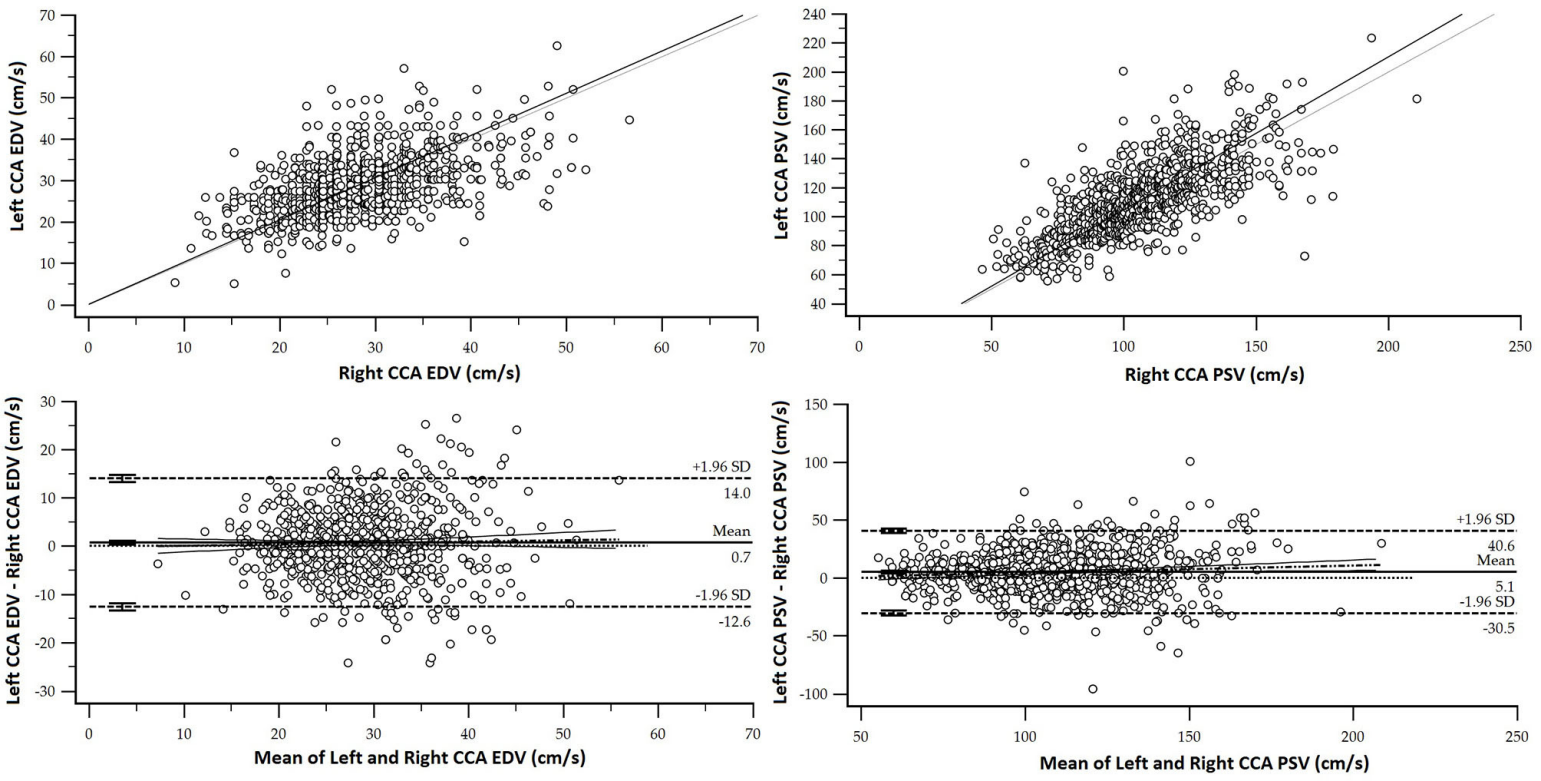
Top. Association (scatter diagram) between left and right CCA and EDV and PSV for the RIs subgroup. Bottom. Bland–Altman diagram. There were significant mean (EDV: 0.71 cm/s and PSV: 5.09 cm/s) and proportional errors.

Third, aiming at determining if RIs for a similar BFV index from left and right body sides were necessary, we analyzed the degree of agreement between them by assessing the following (i) concordance correlation coefficients (CCC) and (ii) mean and proportional differences between data (Bland–Altman analysis) (Supplementary Material 1: Tables S5–S10). Figure 3 exemplifies results obtained when comparing left and right CCA PSV and EDV. As a result, specific RIs for left and right CCA PSV and EDV, ICA PSV, and VA PSV and EDV were defined as necessary (Supplementary Material 1: Table S11). Some indexes (e.g., CCA RI) showed significant statistical differences between sides but were not clinically relevant for RIs construction (e.g., differences <1%).

Fourth, we evaluated whether age and/or sex-specific RIs were necessary using multiple linear regression models that included interaction analysis (Sex × Age) with Johnson–Neyman significance regions definition (Supplementary Material 1: Tables S12–S18). Variables “*y*,” “*x*,” and “*w*” (moderating variable) were assigned, respectively, to the BFV indexes, sex, and age. We identified the following indexes that (i) required sex-specific RIs only from a certain age, (ii) required sex-specific RIs regardless of age, (iii) did not require sex-specific RIs, (iv) did not require age and sex-specific RIs (Supplementary Material 1: Table S11). Even in the cases in which a difference was found between the left and right side, and/or by sex, the RIs were also expressed for hemibodies average, as well as for the entire RIs group (both sexes).

**Figure 4:**
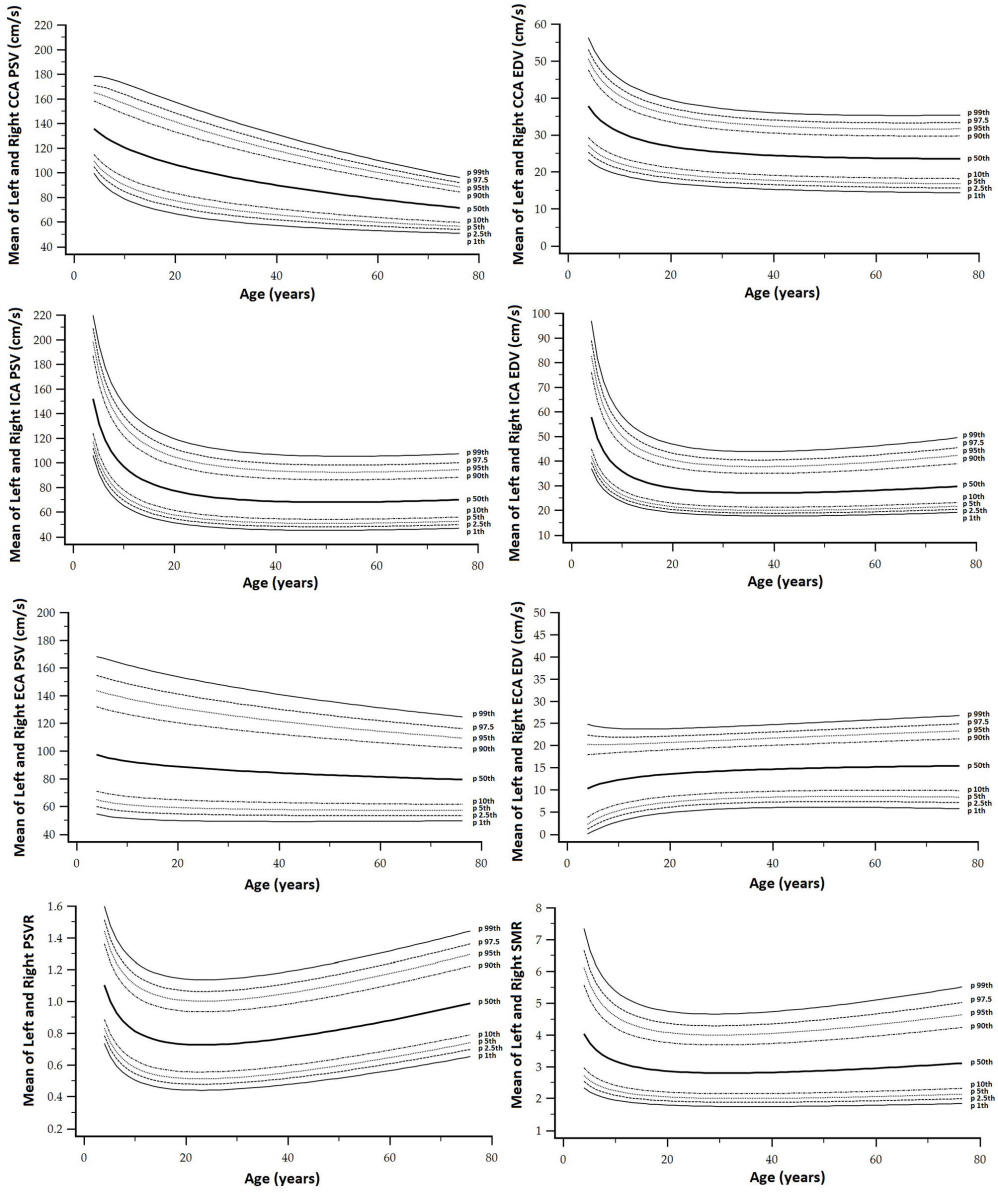
Vertebral artery (VA), CFA, and BA PSV and EDV age-related percentile curves.

**Figure 5:**
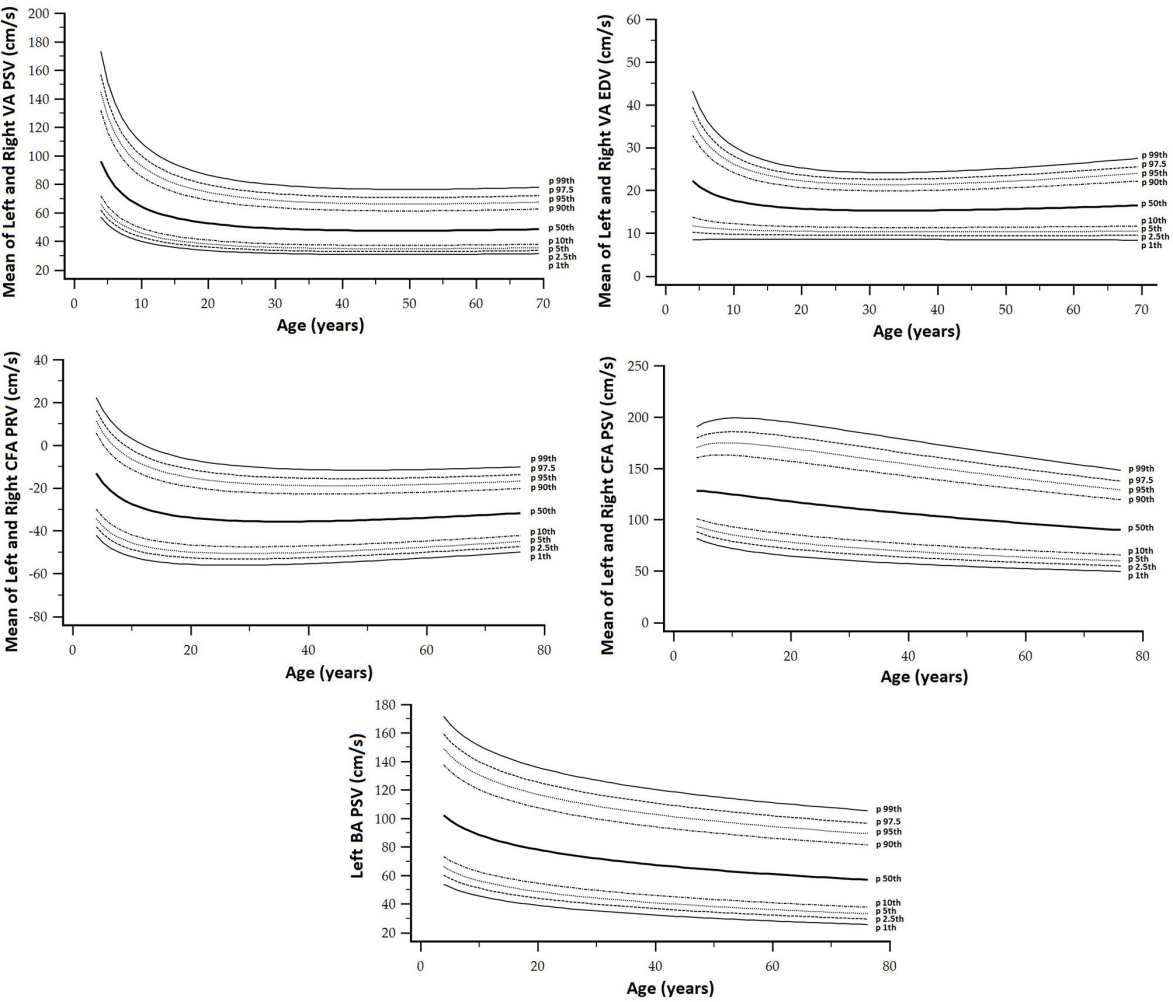
CCA, ICA, and ECA and PSV ratio (PSVR), and St Mary ratio (SMR) age-related percentile curves.

Finally, as a fifth step, age-related percentile curves and RIs were obtained. Age-related equations were obtained for mean values (MV) and SD. To this end, we implemented parametric regression methods based on fractional polynomials (FPs) (Royston and Wright, 1998), included in the European Reference Values for Arterial Measurements Collaboration Group methodological strategy and already used by our group (Diaz et al., 2018; Díaz et al., 2019, 2020; Zócalo et al., 2020; Bia and Zócalo, 2021). Briefly, fitting FPs age-specific MV and SD regression curves for the different variables (e.g., CCA EDV) were defined using an iterative procedure (generalized least squares). Then, age-specific equations were obtained for the different parameters. For instance, the CCA EDV MV equation would be “CCA EDV MV = a + b × Age^p^ + c × Age^q^+…,” where *a, b, c*, are the coefficients, and *p, q*, are the powers, with numbers selected from the set [−2, −1, −0.5, 0, 0.5, 1, 2, 3] estimated from the regression for mean CCA EDV curve, and similarly, from the regression for SD curve. FPs with powers [1,2], that is, with p = 1 and q = 2, illustrate an equation with the form a+b × Age+c × Age^2^ (Royston and Wright, 1998). Residuals were used to assess the model fit, which was deemed appropriate if the scores were normally distributed, with a mean of 0 and an SD of 1, randomly scattered above and below 0 when plotted against age. Best fitted curves, considering visual and mathematical criteria (Kurtosis and Skewness coefficients) were selected. Using the equations obtained for MV and SD (Supplementary Material 1: Table S19), age-specific percentiles were defined using the standard normal distribution (*Z*). The 1st, 2.5th, 5th, 10th, 25th, 50th, 75th, 90th, 95th, 97.5th, and 99th percentiles were calculated, for example for CCA EDV: mean CCA EDV+Zp × SD, where Zp assumed the values −2.3263, −1.9599, −1.6448, −1.2815, −0.6755, 0, 0.6755, 1.2815, 1.6448, 1.9599, and 2.3263, respectively. Year-by-year RIs data can be found in Supplementary Material 1: Tables S20–S105. Figures 4, 5 illustrate percentile curves obtained for VA, CFA, BA, CCA, ICA, ECA, PSVR, and SMR. Supplementary Material 2 (Figures S1–S33) shows BFV indexes age-related percentile curves (for all, women and men, and for left and right side, when appropriate).

The obtained percentiles were compared with data from other works, and the fixed cut-off values recommended to identify arterial stenosis > 50% (**Figures 7–12**) are as follows : (i) CCA PSV <150 or <182 cm/s (Freire et al., 2015), (ii) ICA PSV <125 or <140 cm/s (Oates et al., 2009; Freire et al., 2015), (iii) ICA EDV <40 cm/s (Freire et al., 2015), (iv) ECA PSV <150 cm/s (Freire et al., 2015), (v) VA PSV <85 cm/s (Santos et al., 2019), (vi) VA EDV <27 cm/s (Santos et al., 2019), PSVR <2.0 (Oates et al., 2009), SMR <8.0 (Oates et al., 2009), and CFA PI > 4 (Hodgkiss-Harlow and Bandyk, 2013).

The minimum sample size required was 377 (Bellera and Hanley, 2007). Like in previous works and according to the central limit theorem, normal distribution was considered (taking into account Kurtosis and Skewness coefficients distribution and sample size <30) (Lumley et al., 2002). Data analysis was done using MedCalc-Statistical Software (version 18.5, MedCalc Inc., Ostend, Belgium) and IBM-SPSS software (version 26, SPSS Inc., IL, USA). PROCESS version 3.5 (SPSS extension) was used for moderation (interaction) analysis (Hayes, 2020). A *p* < 0.05 was considered statistically significant.

## Results

### BFV and Atherosclerotic Plaques Presence in Asymptomatic Subjects

Several BFV levels, ratios, and indexes were associated with atherosclerotic plaque presence (Supplementary Material 1: Tables S3, S4).

Peak systolic velocity of BA and PRV of CFA were associated with atherosclerotic plaque presence in any of the studied segments (Supplementary Material 1: Table S3).

Right and left ICA PSV and EDV were positively associated with plaque presence in the evaluated segment and in CCA. In turn, ICA RI was associated with the existence of plaques in ICA and ECA. The associations described for ICA were not observed in CCA (Supplementary Material 1: Table S4). Then, the hemodynamic impact of atherosclerotic plaques presence would be greater in the former.

On the other hand, ICA/CCA ratios were associated with plaque presence in different arterial segments. The finding of atherosclerotic plaques in any segment, as well as in CCA, was associated with VA PSV level (irrespective of age), indicating that plaques in the carotid system may be associated with increased velocities in the vertebro-basilar territory (Supplementary Material 1: Table S4).

### BFV and Hemibody and/or Sex-Related Differences

For some BFV indexes, data from the right and left sides were not equivalent (CCC and Bland-Altman tests) (Supplementary Material 1: Tables S5–S11). However, despite the statistical significance, in general the differences would not be clinically significant. In this regard, the statistically significant differences were (mean error) as follows: (i) 5.1 and 0.7 cm/s for CCA PSV and EDV, (ii) 1.5 cm/s for ICA PSV, (iii) 0.5 cm/s for ECA EDV, and (iv) 4.89 and 1.76 cm/s for VA PSV and EDV (the highest values were always obtained from left hemibody) (Supplementary Material 1: Tables S5–S11).

On the contrary, there were no statistical differences between RI, PI, and SDI data from left and right hemibodies, or they were not clinically significant (e.g., 0.005 for CCA RI). Then, in addition to hemibody-specific RIs, we opted for defining RIs for left- and right-averaged data (Supplementary Material 1: Table S11).

We identified the following indexes that: (i) did not require sex-specific RIs (e.g. CCA EDV), (ii) did not require age and sex-specific RIs (e.g., BA EDV), (iii) require sex-specific RIs regardless of age (e.g., CCA PSV), and (iv) require sex-specific RIs only from a certain age (e.g., ICA EDV) (Supplementary Material 1: Table S11). It is to note, that compared to men, women showed the following: (i) lower CCA PSV or ECA EDV levels for almost all ages; (ii) lower CCA RI, PI and SDI, although values tend to be similar at 5–7 and over <70 years, (iii) lower ICA, PSV and PI, mainly in children, adolescents and young adults, (iv) higher PSVR, although at lower and higher ages the levels tend to be similar for both sexes, (v) similar CFA PSV values at low ages and then levels that exceeded those of men increasingly with age (Supplementary Material 1: Tables S12–S18).

### Age-, Body Side- and Sex-Related RIs

**Figure 6:**
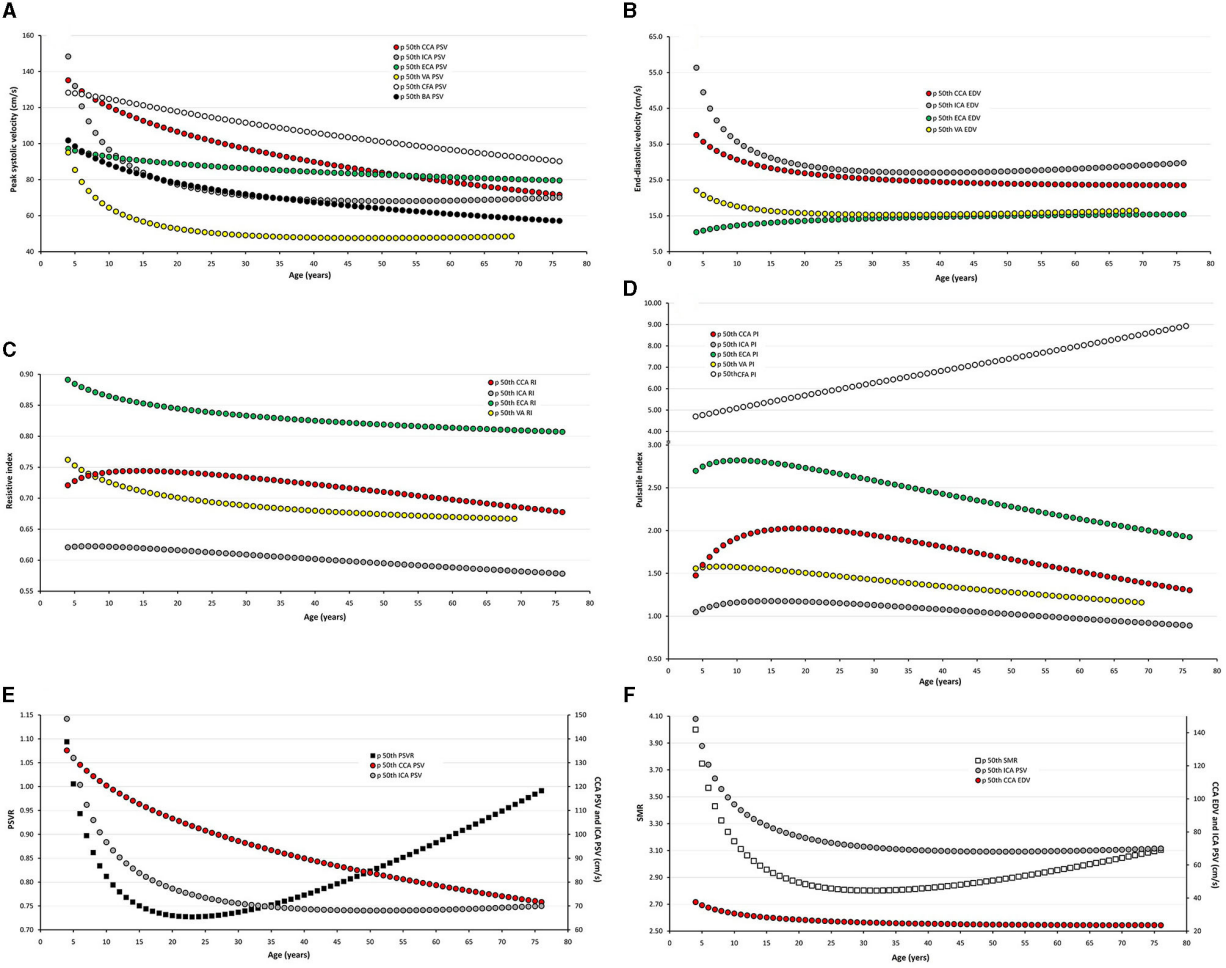
Age-related percentile 50th curves for PSV **(A)**, EDV **(B)**, RI **(C)**, PI **(D)**, PSVR **(E)**, and SMR **(F)**.

**Figure 7:**
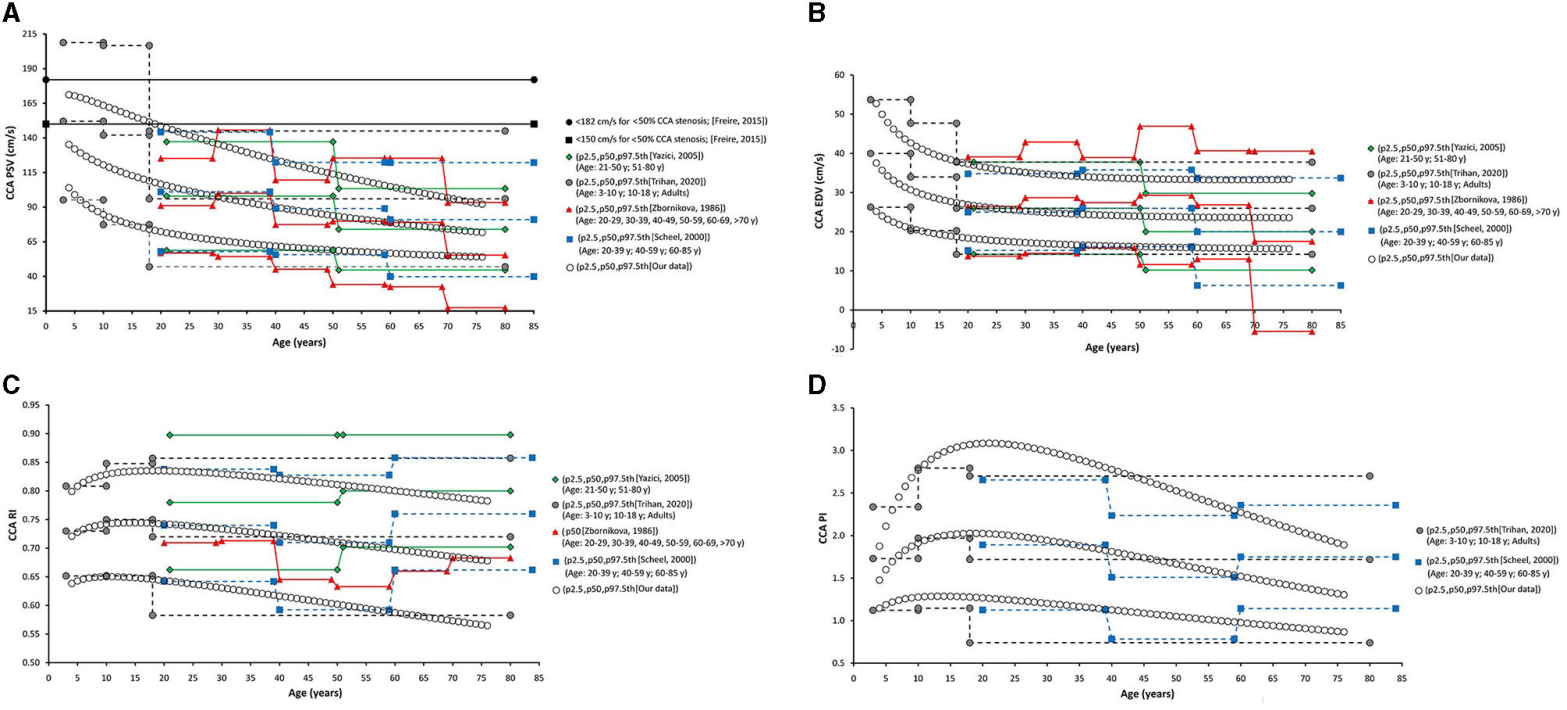
Common carotid artery. Comparison among curves of PSV **(A)**, EDV **(B)**, RI **(C)**, and PI **(D)**, obtained in our study (2.5th, 50th, and p97.5th percentiles) and mean values (p50th), and 2.5th (MV-1.96 × SD) and 97.5 (MV+1.96 × SD) percentiles obtained by other authors when studying groups of subjects of different ages. The fixed cut-off levels used to identify (graduate) levels of arterial stenosis are also shown.

**Figure 8:**
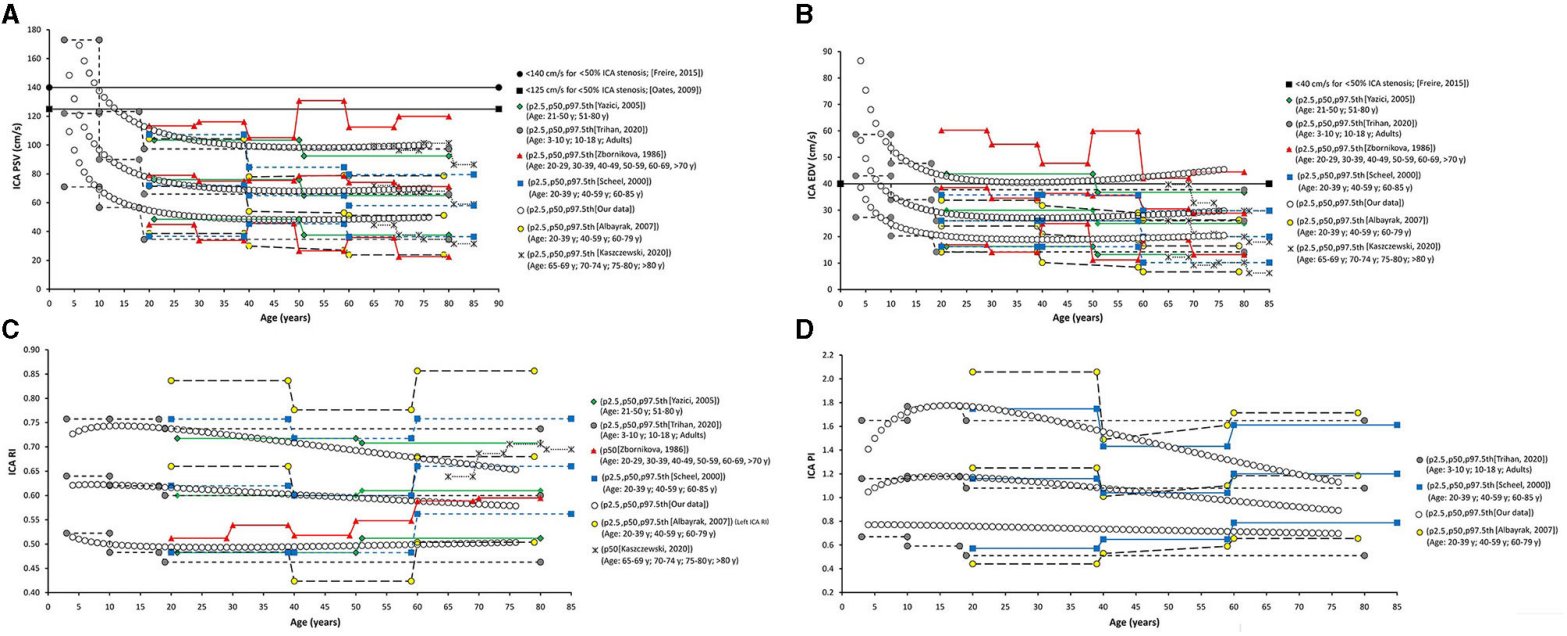
Internal carotid artery. Comparison among curves of PSV **(A)**, EDV **(B)**, RI **(C)**, and PI **(D)**, obtained in our study (2.5th, 50th, and p97.5th percentiles) and mean values (p50th), and 2.5th (MV-1.96 × SD) and 97.5 (MV+1.96 × SD) percentiles obtained by other authors when studying groups of subjects of different ages. The fixed cut-off levels used to identify (graduate) levels of arterial stenosis are also shown.

**Figure 9:**
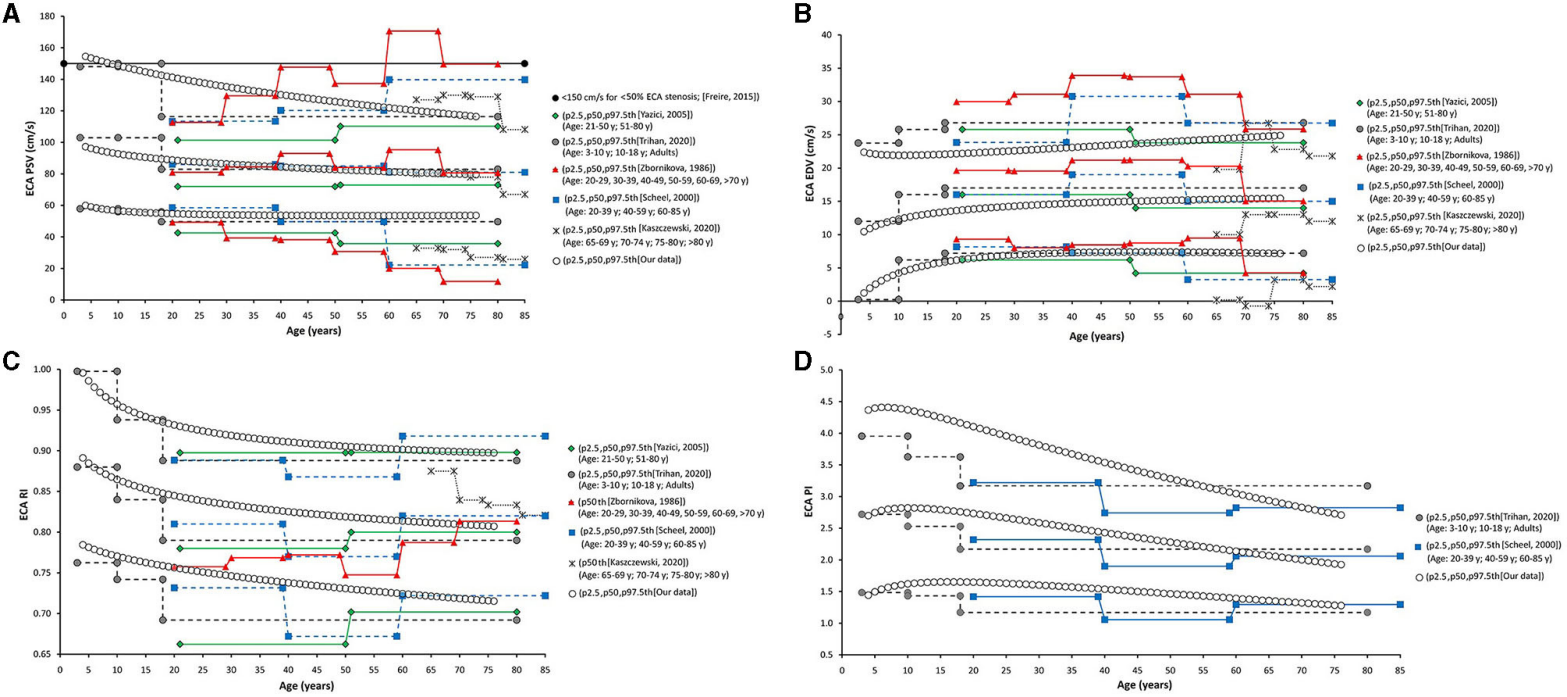
External carotid artery. Comparison among curves of PSV **(A)**, EDV **(B)**, RI **(C)**, and PI **(D)**, obtained in our study (2.5th, 50th, and p97.5th percentiles) and mean values (p50th), and 2.5th (MV–1.96 × SD) and 97.5 (MV+1.96 × SD) percentiles obtained by other authors when studying groups of subjects of different ages. The fixed cut-off levels used to identify (graduate) levels of arterial stenosis are also shown.

**Figure 10:**
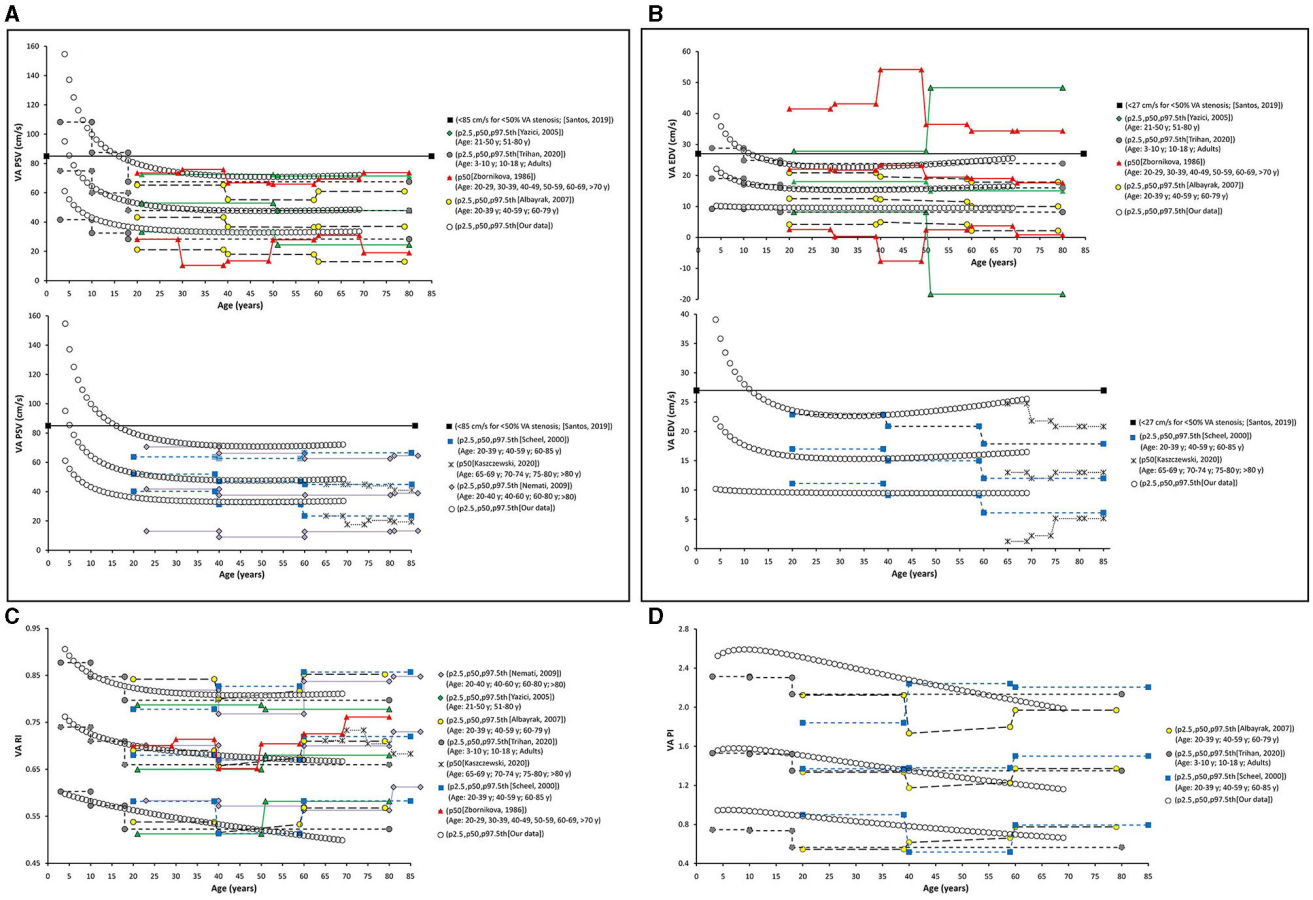
Vertebral artery. Comparison among curves of PSV **(A)**, EDV **(B)**, RI **(C)**, and PI **(D)**, obtained in our study (2.5th, 50th and p97.5th percentiles) and mean values (p50th), and 2.5th (MV−1.96 × SD) and 97.5 (MV+1.96 × SD) percentiles obtained by other authors when studying groups of subjects of different ages. The fixed cut-off levels used to identify (graduate) levels of arterial stenosis are also shown.

**Figure 11:**
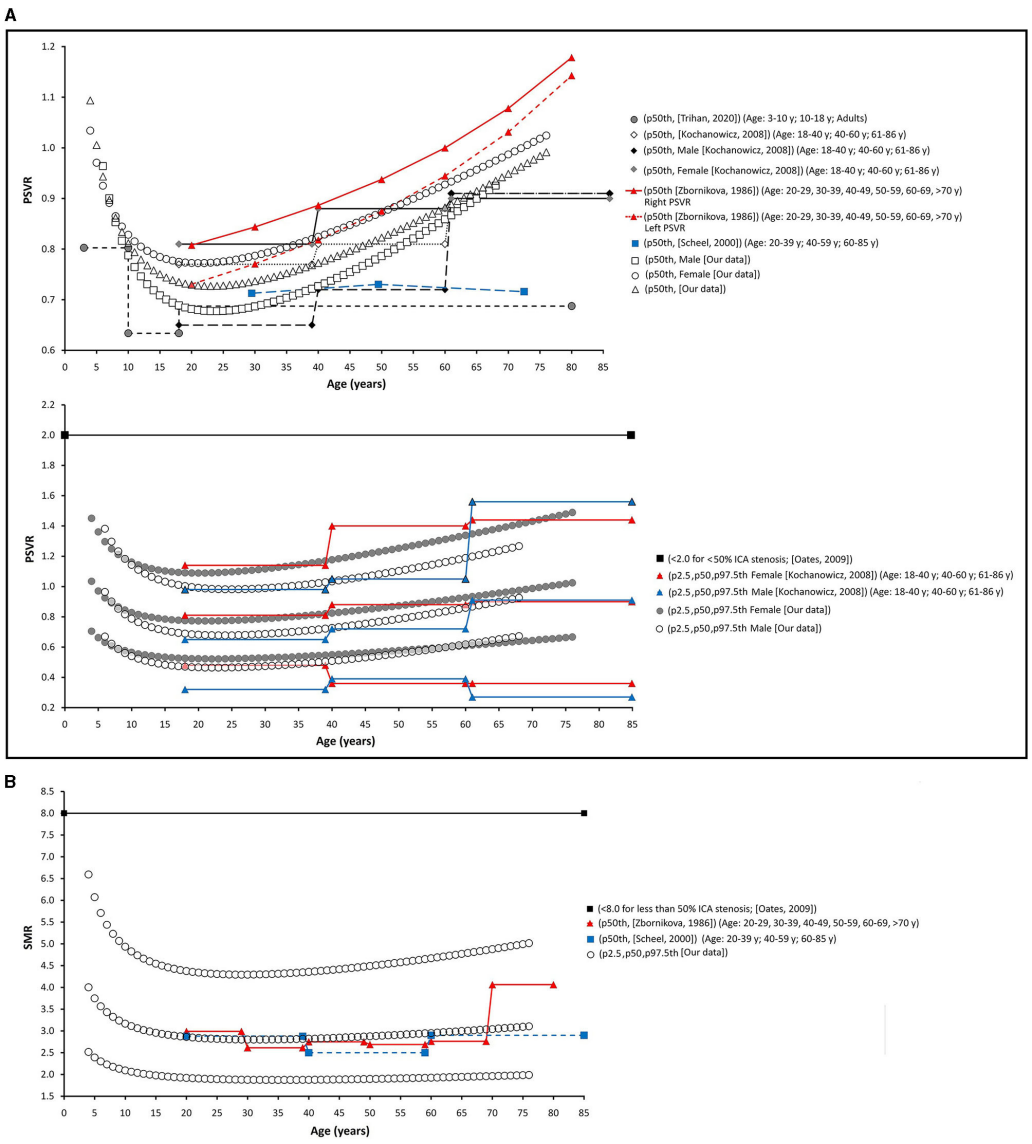
Internal/common carotid artery velocity ratios. Comparison between curves of PSVR **(A)**, and SMR **(B)** obtained in our work (2.5th, 50th, and p97.5th percentiles) and mean values (p50th), and 2.5th (MV−1.96 × SD) and 97.5 (MV+1.96 × SD) percentiles obtained by other authors when studying groups of subjects of different ages. The fixed cut-off levels used to identify (graduate) levels of arterial stenosis are also shown.

**Figure 12:**
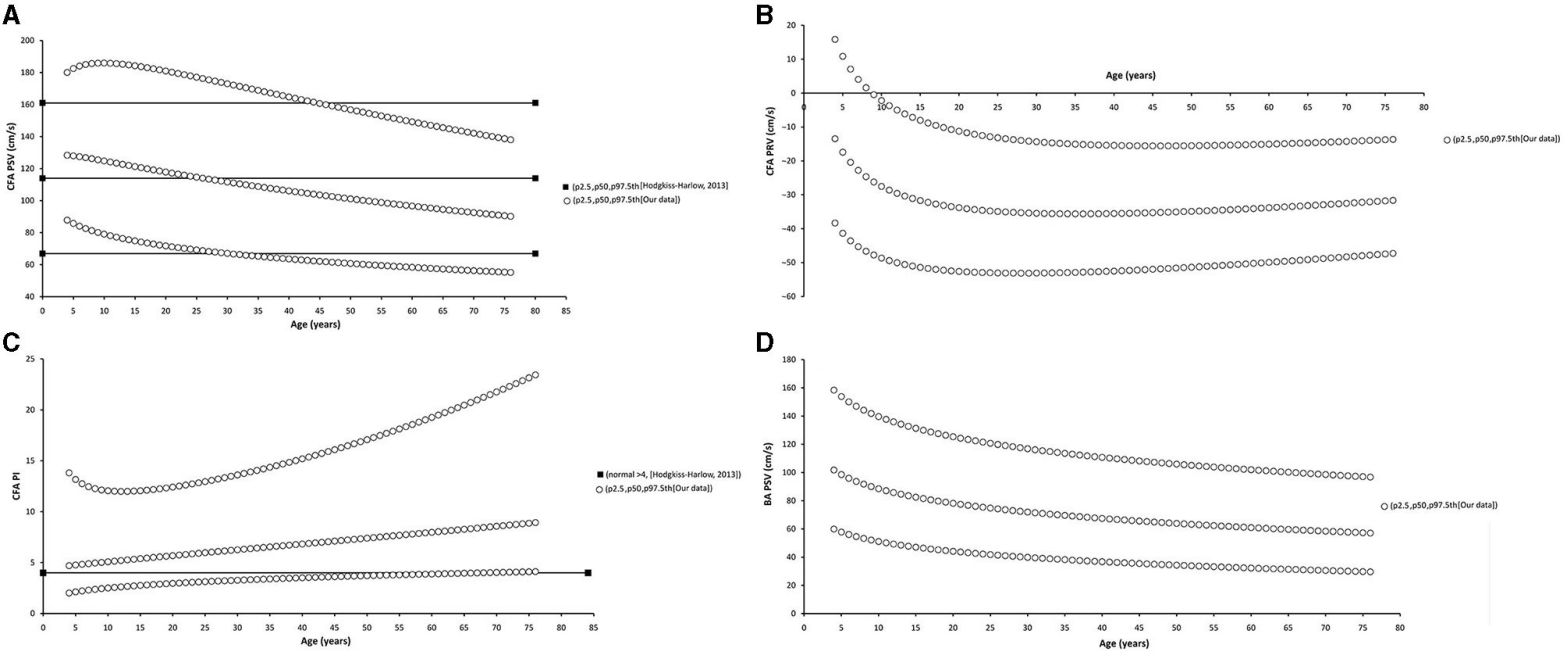
Common femoral artery (CFA) and brachial artery (BA). Comparison between curves of CFA PSV **(A)**, CF PRV **(B)**, CFA PI **(C)**, and BA PSV **(D)** obtained in our study (2.5th, 50th, and p97.5th percentiles) and mean values (p50th), and 2.5th (MV−1.96 × SD) and 97.5 (MV+1.96 × SD) percentiles obtained by other authors when studying groups of adults. The fixed cut-off levels used to identify (graduate) levels of arterial stenosis are also shown.

Data for year-by-year RIs can be seen in Supplementary Material 1: Tables S20–S105. The 1st, 2.5th, 5th, 10th, 25th, 50th, 75th, 90th, 95th, 97.5th, and 99th percentiles were calculated. Equations for sex, body side, and age-specific percentiles were included in text and spreadsheet formats. Age-related profiles (considering both sexes, p50th) for the different arterial segments and BFV indexes are shown in Figure 6. The comparison of this work data (p2.5th, p50th and p97.5th) and that obtained by other authors [p50th (MV), p2.5th (MV−1.96 × SD), and p97.5th (MV+1.96 × SD)] is shown in Figures 7–**12**.

When comparatively analyzing p2.5th, p50th, and p97.5th curves obtained in this work with fixed cut-off values recommended to detect arterial stenosis >50%, the following was observed: (i) at low ages (children and/or adolescents) the thresholds were generally exceeded, whereas (ii) at old ages cut-off levels exceeded p97.5th values (more so at older ages) (Figures 7–**12**).

## Discussion

### Age-Related Profiles

#### Carotid and Vertebral Arteries PSV and EDV

Common carotid artery ICA, ECA, and VA PSV levels were lower at higher ages but there were “regional” differences in the age-related profiles of arteries. The greatest reduction was observed in childhood/adolescence, however, whereas CCA PSV showed a continuous fall, ICA, ECA, and VA PSV levels stabilized at about 30–35 years (Figure 6A). The ratio between PSV levels in the carotid arteries showed age-related variations. During childhood ICA PSV was higher than ECA PSV, but approximately at 10–15 years of age, the relationship reversed and, thereafter, ECA PSV remained higher than ICA PSV.

The ICA and VA PSV showed similar age-related profiles, but ICA PSV levels were always higher than those of the VA (Figure 6A). ECA EDV increased, whereas CCA, ICA, and VA EDV showed a reduction during childhood and adolescence. On the other hand, disregard of the arterial segment considered, the values stabilized at about 30–35 years (Figure 6B). CCA and ICA EDV level, as well as VA and ECA EDV remained at similar levels from ~20–30 years onward.

In general terms, age-related PSV and EDV values and variations observed for CCA (Figures 7A,B), ICA (Figures 8A,B), ECA (Figures 9A,B), and VA (Figures 10A,B) were in agreement with data (and differences) reported for children, adolescents, and/or adults (Zbornikova and Lassvik, 1986; Scheel et al., 2000; Yazici et al., 2005; Albayrak et al., 2007; Demirkaya et al., 2008; Nemati et al., 2009; Kaszczewski et al., 2020; Trihan et al., 2020). In this regard, it should be noted that previous works compared (statistically) mean values obtained for groups of subjects within a wide age range [e.g., intervals of 10 (Zbornikova and Lassvik, 1986), 20 (Scheel et al., 2000), or 30 years (Yazici et al., 2005)], which would result in the finding of “step curves” that do not allow to visualize gradual physiological age-associated variations in BFV indexes. At the same time, the percentile curves obtained could show “deviations” that would not represent the real age-associated variations, determining (in some cases) non-physiological percentiles (e.g., CCA EDV p2.5th reached negative values) (Figure 7B).

#### Carotid and Vertebral Arteries RI and PI

In the first decades of life, RI and PI of carotid and vertebral arteries showed different behaviors (increases or falls), but they subsequently showed a gradual reduction with age (Figures 6C,D). CCA RI reached a maximum approximately at 15–20 years and then it fell to values even lower than the observed in childhood. The above described agrees with previous works in children, adolescents, and young or middle-aged adults (Zbornikova and Lassvik, 1986; Scheel et al., 2000; Trihan et al., 2020) (Figures 7C,D).

On the other hand, some works described an increase in CCA RI and/or PI in subjects over 60 years (Zbornikova and Lassvik, 1986; Scheel et al., 2000) (Figure 7C). This could be explained by the inclusion of subjects with atherosclerotic plaques in ICA and/or ECA (whose prevalence increases with age) (Scheel et al., 2000). A similar consideration applies to ICA and ECA (see below).

Data obtained for ICA RI and PI (Figures 8C,D) agree with previous results (Trihan et al., 2020). Like us, other authors reported an age-related reduction in ICA RI and PI (Yazici et al., 2005; Trihan et al., 2020). However, works in which subjects with atherosclerotic plaques were not excluded showed an increase in ICA RI in <60 years (Figures 8C,D).

External carotid artery RI and PI showed an age-related reduction (mainly in the first two decades of life) (Trihan et al., 2020). Age-associated changes in ECA RI have been described in a limited way, and the available data are not coincident (e.g., interspersed increases and decreases with variations depending on the age group considered have been reported) (Zbornikova and Lassvik, 1986; Scheel et al., 2000) (Figures 9C,D).

#### “Inter-Segment” Velocity Ratios: PSV and St Mary's Ratio

Peak systolic velocity ratio decreased until approximately 20–25 years (Figure 6E), and, thereafter, it increased markedly and steadily. Despite both, CCA and ICA PSV decreased with age in the following stages: (i) in the first decade of life there was a reduction in PSVR, mainly explained by the significant reduction in ICA PSV, whereas (ii) from 20–25 years onward, it was observed an increase in PSVR, mainly attributed to the reduction in CCA PSV (Figure 6E). This agrees with data previously obtained in children and adolescents (Trihan et al., 2020) and in adults (Zbornikova and Lassvik, 1986; Kochanowicz et al., 2009) (Figures 11A,B).

St Mary's ratio decreased until about 10–15 years (mainly due to the reduction in ICA PSV) and thereafter stabilized at 3.0. This agrees with data and trends previously described (Zbornikova and Lassvik, 1986; Scheel et al., 2000) (Figures 6F, 11C).

#### Common Femoral and Brachial Artery Velocity and Indexes

There was a sustained age-related reduction in CFA PSV (Figures 6A, 12A). This could be associated with a reduction in cardiac ejection rate.

During the first two decades of life, there was an age-related reduction in CFA PRV. Thereafter, it stabilized or showed a slight increase (Figure 12B). The degree of PRV variation (reversal level) in the first two decades would result from the interplay between vascular changes that would lead to higher (e.g., age-related increase in arterial stiffness and peripheral-to-aortic pressure gradient) or lower (e.g., age-related increase in arterial diameters) wave reflection levels (Hashimoto and Ito, 2010).

Opposite to the described carotid and vertebral arteries, there was a continuous age-associated increase in CFA PI. Although CFA PSV decreased with age, CFA PI increased, mainly due to an increase in forward–backward velocity oscillation around a mean value that decreases with age. It is to note that p2.5th for CFA PI data in adults overlapped the cut-off value (CFA PI = 4) proposed to define lower limb perfusion normality (Hodgkiss-Harlow and Bandyk, 2013) (Figure 12C).

There was a gradual age-related reduction in left BA PSV (Figures 6, 12D), which is in line with the described for other arteries.

### Body Side-Related Differences

Despite for some BFV indexes there were statistical differences between right and left hemibodies data, the differences would not be significant in clinical practice. Previous works regarding this issue showed dissimilar findings, but in case there were differences, they were small.

Scheel et al. reported no significant side-to-side differences in flow velocities and waveform parameters in paired extracranial vessels (CCA, ICA, EC and VA), in a work that included 78 healthy adults (age MV/SD: 52/19 years, range: 20–85 years, both sexes) (Scheel et al., 2000). In turn, in healthy children and adolescents, Schoning and Hartig found no significant side-to-side differences in extracranial carotid velocity indexes (Schoning and Hartig, 1998). Other authors found higher flow velocities in left than in right CCA (Donis et al., 1988; Scheel et al., 2000) and VA (Nemati et al., 2009). Finally, higher PSV and EDV were observed in right than in the left ICA (Zbornikova and Lassvik, 1986).

There were no differences between left and right SMR, whereas the statistical differences in PSVR (higher values on the right side) would be clinically negligible (Supplementary Material 1: Table S11). This is in agreement with Kochanowicz et al. (Kochanowicz et al., 2009). In a population of healthy subjects (*n* = 343, mean age/SD: 43.5/18.3 years, range: 18–86 years) the authors did not find significant differences between left and right carotid ratios. Then, they proposed using the average of data from both hemibodies to define the reference limits for ICA/CCA ratios (Kochanowicz et al., 2009). Zbornikova and Lassvik did not find differences in PSVR between left and right sides (right: 1.00 ± 0.34, left: 0.96 ± 0.36), but they found SMR was higher in the right than in the left (3.93 ± 1.47 vs. 3.23 ± 1.29) which was explained by higher right ICA PSV (Zbornikova and Lassvik, 1986).

### Sex-Related Differences

We identified the following indexes: (i) that did not require sex-specific RIs (e.g., CCA EDV), (ii) that did not require age and sex-specific RIs (e.g., BA EDV), (iii) that required sex-specific RIs regardless of age (e.g., CCA PSV), and (iv) that required sex-specific RIs only from a certain age (e.g., ICA EDV) (Supplementary Material 1: Table S11). Then, there was not a uniform behavior enabling to standardize the need for sex or age-related RIs, but it varied depending on the BFV index considered.

The finding of age-dependent differences between men and women (“age × sex” interaction), could contribute to explain the dissimilar or controversial findings. Scheel et al. reported non-significant sex-related differences in flow velocity- and waveform-derived parameters from extra-cranial vessels (Scheel et al., 2000). Zbosnikovaand Lassvick found (small) differences between women and men BFV (e.g., higher ICA PSV and EDV in women on the right-side) (Zbornikova and Lassvik, 1986). In turn, no sex-related differences were described for intracranial arteries (middle, anterior, and posterior cerebral arteries) BFV (PSV, EDV, Vm) (Hashimoto and Ito, 2010). It is to note that similar to our work, Kochanowicz et al. reported data suggesting “age × sex” interaction (Kochanowicz et al., 2009). The authors found higher PSVR in women than in men for the age groups I (>40 years) and II (40–60 years), whereas similar ratios were observed for men and women in the age group III (>60 years). Then, sex-related differences would be moderated by age. Similarly, in this work p50th for PSVR in women and men showed differences in young or middle-aged adults, whereas data overlapped in older subjects (Figure 11B).

At least, in theory, the differences in BFV indexes age-related profiles observed between men and women could be explained by different age-related changes in CCA and ICA structure, and in the configuration of CCA bifurcation. In this regard, in women, ICA and CCA showed similar age-related dilatation, whereas, in men, CCA dilatation was greater and earlier than that of the ICA (Kochanowicz et al., 2009).

### Percentiles vs. Fixed Cut-Off Points Indicative of Arterial Stenosis

The fixed threshold proposed for CCA PSV (150 cm/s) would not be useful for subjects <30 years, whose CCA PSV levels are usually above that value (Figure 7A). On the other hand, the fixed cut-points (150 and 182 cm/s) are far above p97.5th for adults aged 50, 60, and 70 years (114, 105, and 97 cm/s, respectively). Similar considerations apply to ICA PSV (120 and 140 cm/s) and ECA PSV (150 cm/s) thresholds (Figures 8A, 9A). Although proposed thresholds would show high specificity for detecting significant stenosis and/or PSV deviations from expected values, they would not allow for early detection of vascular changes associated with disease and/or increased risk.

On the contrary, the threshold proposed for ICA EDV (40 cm/s) was below or overlapped the p97.5th obtained for children, adolescents, and adults (in several studies, including this one) (Figure 8B). Then, the use of the described cut-off point could be associated with over-diagnosis or over-estimation of the stenosis in subjects with atherosclerotic plaques.

Thresholds for VA PSV and EDV (85 and 27 cm/s) were below frequent values for children and adolescents, whereas they were near the p97.5th for adults (like in carotid arteries) (Figures 10A,B). Cut-off points for PSVR (2.0) and SMR (8.0) were far from the maximum values expected for any age (Oates et al., 2009). Therefore, they would not allow for detecting small and/or gradual departures from the “expected-for-age.” (Figures 11B,C)

Common femoral artery PSV percentiles reported by Hodgkiss-Harlow and Bandyk (2013) were below or above the p97.5th obtained in this study for subjects younger or older than 45–50 years. On the other hand, fixed percentiles should not be used in CFA since PSV would not stabilize at any age. Finally, approximately after 45 years, CFA PI values ≤ 4 overlapped p2.5th values. Then, values above the threshold would be frequently found in healthy subjects (Hodgkiss-Harlow and Bandyk, 2013).

### Clinical and Physiological Implications

Atherosclerosis evolves asymptomatically over time. Timely preventive strategies would limit disease progression and reduce the risk of complications (e.g., cardiovascular events) (Naghavi et al., 2006). US B-mode evaluation and Doppler-derived data are commonly used for the following: (i) to differentiate normal from diseased vessels, (ii) to define disease stages, (iii) to assess collateral circulation (e.g., cerebral), doing so safely and cost-effectively. A primary aim of evaluations is to identify subjects with vascular conditions/disease associated with increased risk of cardiovascular events (e.g., stroke, vertebro-basilar ischemia), who would require specific treatments, and/or who would (particularly) benefit from preventive strategies. Another important aim is to document progressive or recurrent disease in patients already known to be at risk (Gerhard-Herman et al., 2006). In addition, it has been recently proposed that US studies could be used to identify subjects at increased risk, without the advanced disease (e.g., non-significant arterial narrowing) (Kochanowicz et al., 2009). In this sense, the deviation of BFV indexes of a particular subject from expected values could help to diagnose early stages of vascular disease and to determine individual risk. In this context, this work provides RIs for PSV, EDV, PRV, RI, PI, SDI, PSVR, and SMR data from different arterial segments (CCA, ICA, ECA, VA, CFA, and BA) obtained (in a single instance) from US studies performed in a large population of healthy children, adolescents, and adults.

Aiming at contributing with other groups and/or researchers, body side- sex- and age-related equations for MV, SD, and percentiles values were included in text and spreadsheet formats (Supplementary Materials). Thus, expected values for a given subject (and *z*-scores) could be calculated for clinical and/or research purposes.

Finally, it is to note that this work adds to the knowledge of the physiological variations in BFV levels and profiles that would be expected during growth and aging, analyzing at the same time (and comparatively) the behavior of different arteries. Furthermore, potential differences between left and right hemibodies as well as between men and women were considered and analyzed.

### Strengths and Limitations

Our results should be understood within the context of the strengths and limitations that could be ascribed to the present work. First, as the work is a cross-sectional one, it provides no data on longitudinal age-related changes in BFV related indexes. Second, since no outcome data were considered, cut-off points (e.g., p75th, p90th, p95th) could not be defined based on CVR, but on values distribution in the studied group. It is not known whether the reference values should be used as cut-off values for diagnosis and treatment. In any case, if reference data were to be used in clinical practice it would be valuable to have a clear understanding of the factors that could modify the indexes levels and profiles. In this regard, further works would be necessary to define the impact that the exposure to traditional and emergent (new) CRFs, known to affect arterial vessels during aging, could have on BFV levels and indexes and their age-related variations. The analysis should be done taking into account factors influencing the findings (e.g., time of exposure, severity, the tendency of CRFs to aggregate and interact). Third, for any age, hemodynamic, structural, and functional vascular parameters can be acutely and temporarily modified by variations in the vascular smooth muscle tone (Bia et al., 2003, 2008). Systematization of recording conditions is necessary for the evaluation of arterial properties considering the modulating role of the smooth muscle tone. In this study, to systematize the records and as a way to minimize the impact of the referred source of variability, BFV levels were assessed and determined at rest under stable hemodynamic state conditions.

A major strength of the work is that BFV parameters were obtained in a large population sample (including children, adolescents, and adults), which enabled to define trends in mean values and percentiles for almost the whole range of life expectancy. The obtained data contribute to characterize the behavior of BFV (levels and parameters) in different arterial segments throughout life, providing information useful in clinical practice and physiological research. In this context, it is worth recalling that subjects with atherosclerotic plaques as well as those with ABI <0.9 were excluded from this study. This reinforces the value of the obtained data in terms of contribution to characterizing hemodynamic vascular parameters in physiological conditions.

## Conclusion

This study adds to the knowledge of the physiological variations in BFV that would be expected during growth and aging, analyzing at the same time (and comparatively) the behavior of different indexes and arteries. Sex- and age-related profiles and RIs (normative data) for BFV levels and indexes obtained from US recordings of brachial, vertebral, carotid (common, internal, and external), and femoral arteries were determined in a large population (comprising children, adolescents, and adults) of asymptomatic subjects non-exposed to CRFs. Data (percentile curves) were compared with fixed thresholds recommended for use in clinical practice. Equations (for mean and SD values; spreadsheet formats) were given to enable researchers and/or clinicians to determine expected values and potential deviations.

The presence of atherosclerotic plaques was associated (in asymptomatic subjects non-exposed to CRFs) with BFV levels, intrabeat indexes, and intersegment ratios. Some BFV-derived indexes showed statistical differences between hemibodies but the differences would not be significant in clinical practice. There was no uniform behavior to enable standardize the need for sex-related RIs (normative data), the need for sex-specific BFV RIs relied on the index and/or age considered.

Peak systolic velocity levels were lower at higher ages but there were “regional” differences in the age-related profiles of the arteries. The greatest reduction was observed in childhood/adolescence; however, though CCA, CFA, and BA PSV showed a continuous fall, ICA, ECA, and VA PSV levels stabilized at about 30–35 years. In the first decades of life, RI and PI of carotid and vertebral arteries showed different behaviors (increases or falls), but they subsequently showed a gradual reduction with age. In contrast, there was a continuous age-associated increase in CFA PI.

Currently used fixed thresholds would show high specificity for detecting significant stenosis and/or PSV deviations from expected values, but could result in misdiagnosis and/or would not allow detecting small and/or gradual departures from “expected-for-age,” which could be associated with disease and/or increased risk.

## Data Availability Statement

The original contributions presented in the study are included in the article/Supplementary Material, further inquiries can be directed to the corresponding author.

## Ethics Statement

The studies involving human participants were reviewed and approved by Comité de Ética de Investigación, Centro Hospitalario Pereira-Rossell, ASSE, Universidad de la República. Written informed consent to participate in this study was provided by the participants' legal guardian/next of kin.

## Author Contributions

YZ and DB contributed to conception and design of the study, performed the cardiovascular non-invasive recordings, constructed and organized the database, performed the statistical analysis, wrote the first draft and final version of the manuscript, contributed to manuscript revision, read, and approved the submitted version. Both authors contributed to the article and approved the submitted version.

## Conflict of Interest

The authors declare that the research was conducted in the absence of any commercial or financial relationships that could be construed as a potential conflict of interest.

## Publisher's Note

All claims expressed in this article are solely those of the authors and do not necessarily represent those of their affiliated organizations, or those of the publisher, the editors and the reviewers. Any product that may be evaluated in this article, or claim that may be made by its manufacturer, is not guaranteed or endorsed by the publisher.

## Abbreviations

ABI: ankle brachial index
BA: brachial artery
bDBP: brachial diastolic blood pressure
BFV: blood flow velocity
BH: body height
bMBP: brachial mean blood pressure
BMI: body mass index
BP: blood pressure
bPP: brachial pulse pressure
bSBP: brachial systolic blood pressure
BW: body weight
CCA: common carotid artery
CCC: concordance correlation coefficient
CFA: common femoral artery
CRFs: cardiovascular riskfactors
CVD: cardiovascular disease
CVR: cardiovascular risk
ECA: external carotid artery
EDV: end-diastolic blood flow velocity
ICA: internal carotid artery
MV: mean value
PI: pulsate index
PRV: peak reversal velocity
PSV: peak systolic blood flow velocity
PSVR: peak systolic velocity ratio
RI: resistive index
RIs: reference intervals
SD: standard deviation
SDI: systo-diastolic index
SMR: St Mary's ratio
taDBP: tibial artery diastolic blood pressure
taSBP: tibial artery systolic blood pressure
US: ultrasound or ultrasonographic
VA: vertebral artery
Vm: mean blood flowvelocity.

## References

[B1] Albayrak R. Degirmenci B. Acar M. Haktanir A. Colbay M. Yaman M. (2007). Doppler sono-graphy evaluation of flow velocity and volume of the extracranial internal carotid and vertebral arteries in healthy adults. J. Clin. Ultrasound. 35, 27–33. 10.1002/jcu.20301 17149761

[B2] Bellera C. A. Hanley J. A. (2007). A method is presented to plan the required sample size when estimating regression-based reference limits. J. Clin. Epidemiol. 60, 610–615. 10.1016/j.jclinepi.2006.09.004 17493520

[B3] Bia D. Zócalo Y. (2021). Physiological age- and sex-related profiles for local (aortic) and regional (carotid-femoral, carotid-radial) pulse wave velocity and center-to-periphery stiffness gradient, with and without blood pressure adjustments: reference intervals and agreement between methods in healthy subjects (3–84 years). J. Cardiovasc. Dev. Dis. 8:3. 10.3390/jcdd8010003 33445548 PMC7827252

[B4] Bia D. Zócalo Y. Armentano R. Camus J. Forteza E. Cabrera-Fischer E. (2008). Increased reversal and oscillatory shear stress cause smooth muscle contraction-dependent changes in sheep aortic dynamics: role in aortic balloon pump circulatory support. Acta Physiol. 192, 487–503. 10.1111/j.1748-1716.2007.01765.x 17973954

[B5] Bia D. Zócalo Y. Farro I. Torrado J. Farro F. Florio L.. (2011). Integrated evaluation of age-related changes in structural and functional vascular parameters used to assess arterial aging, subclinical atherosclerosis, and cardiovascular risk in uruguayan adults: CUiiDARTE Project. Int. J. Hypertens. 2011:587303. 10.4061/2011/587303 22187622 PMC3235479

[B6] Bia D.;, Armentano, R. L. Grignola J. C. Craiem D. Zócalo Y. A. Ginés F. F.. (2003). The vascular smooth muscle of great arteries: local control site of arterial buffering function? Rev. Esp. Cardiol. 56, 1202–1209. 10.1016/S0300-8932(03)77039-0 14670273

[B7] Bossuyt J. Engelen L. Ferreira I. Stehouwer C. D. Boutouyrie P. Laurent S.. (2015). Reference values for arterial measurements collaboration. Reference values for local arterial stiffness. Part B: Femoral artery. J. Hypertens. 33, 1997–2009. 10.1097/HJH.0000000000000655 26431186

[B8] Bruno R. M. Nilsson P. M. Engström G. Wadström B. N. Empana J. P. Boutouyrie P.. (2020). Early and supernormal vascular aging: clinical characteristics and association with incident cardiovascular events. Hypertension 76, 1616–1624. 10.1161/HYPERTENSIONAHA.120.14971 32895017

[B9] Castro J. M. García-Espinosa V. Zinoveev A. Marin M. Severi C. Chiesa P.. (2019). Arterial structural and functional characteristics at end of early childhood and beginning of adulthood: impact of body size gain during early, intermediate, late and global growth. J. Cardiovasc. Dev. Dis. 6:33. 10.3390/jcdd6030033 31489955 PMC6787690

[B10] Chuang S. Y. Bai C. H. Chen J. R. Yeh W. T. Chen H. J. Chiu H. C.. (2011). Common carotid end-diastolic velocity and intima-media thickness jointly predict ischemic stroke in Taiwan. Stroke 42, 1338–1344. 10.1161/STROKEAHA.110.605477 21415400

[B11] Chuang S. Y. Bai C. H. Cheng H. M. Chen J. R. Yeh W. T. Hsu P. F.. (2016). Common carotid artery end-diastolic velocity is independently associated with future cardiovascular events. Eur. J. Prev. Cardiol. 23, 116–124. 10.1177/2047487315571888 25691545

[B12] Clifford P. C. Skidmore R. Bird D. R. Woodcock J. P. Baird R. N. (1981). The role of pulsatility index in the clinical assessment of lower limb ischaemia. J. Med. Eng. Technol. 5, 237–241. 10.3109/03091908109018164 6457154

[B13] Demirkaya S. Uluc K. Bek S. Vural O. (2008). Normal blood flow velocities of basal cerebral arteries decrease with advancing age: a transcranial Doppler sonography study. Tohoku J. Exp. Med. 14, 145–149. 10.1620/tjem.214.145 18285672

[B14] Diaz A. Bia D. Zócalo Y. Manterola H. Larrabide I. Lo Vercio L.. (2018). Carotid intima media thickness reference intervals for a healthy argentinean population aged 11–81 years. Int. J. Hypertens. 2018:8086714. 10.1155/2018/8086714 29992052 PMC5832113

[B15] Díaz A. Zócalo Y. Bia D. (2019). Normal percentile curves for left atrial size in healthy children and adolescents. Echocardiography 36, 770–782. 10.1111/echo.14286 30801788

[B16] Díaz A. Zócalo Y. Bia D. (2020). Percentile curves for left ventricle structural, functional and haemodynamic parameters obtained in healthy children and adolescents from echocardiography-derived data. J. Echocardiogr. 18, 16–43. 10.1007/s12574-019-00425-0 30927161

[B17] Donis J. Graf M. Sluga E. (1988). Flussmessungen an den extrakraniellenKarotidenmitHilfe der Duplex-Sonographie. ErgebnissebeiNormalpersonen. Ultraschall Med. 9, 216–222. 10.1055/s-2007-1011629 3051368

[B18] Engelen L. Bossuyt J. Ferreira I. van Bortel L. M. Reesink K. D. Segers P.. (2015). Reference values for arterial measurements collaboration. Reference values for local arterial stiffness. Part A: Carotid artery. J. Hypertens. 33, 1981–1996. 10.1097/HJH.0000000000000654 26431185

[B19] Engelen L. Ferreira I. Stehouwer C. D. Boutouyrie P. Laurent S. (2013). Reference Values for Arterial Measurements Collaboration. Reference intervals for common carotid intima-media thickness measured with echotracking: Relation with risk factors. Eur. Heart J. 34, 2368–2380. 10.1093/eurheartj/ehs380 23186808

[B20] Freire C. M. Alcântara M. Santos S. Amaral S. Veloso O. Porto C. L.. (2015). Recomendação para a quantificação pelo ultrassom da doença aterosclerótica das artérias carótidas e vertebrais: Grupo de trabalho do Departamento de Imagem Cardiovascular da Sociedade Brasileira de Cardiologia-DIC-SBC. Arq Bras. Cardiol. Imagemcardiovasc. 28, e1–64. 10.5935/2318-8219.20150018

[B21] Gerhard-Herman M. Gardin J. M. Jaff M. Mohler E. Roman M. Naqvi T. Z. (2006). Guidelines for noninvasive vascular laboratory testing: a report from the American Society of Echocardiography and the Society for Vascular Medicine and Biology. Vasc Med. 11, 183–200. 10.1177/1358863x06070516 17288127

[B22] Hashimoto J. Ito S. (2010). Pulse pressure amplification, arterial stiffness, and peripheral wave reflection determine pulsatile flow waveform of the femoral artery. Hypertension 56, 926–933. 10.1161/HYPERTENSIONAHA.110.159368 20876451

[B23] Hayes A. F. (2020). Introduction to Mediation, Moderation, and Conditional Process Analysis (2nd Edition). Available online at: http://www.guilford.com/p/hayes3 (accessed on 13th Nov, 2020).

[B24] Hitsumoto T. (2019). Relationships between the cardio-ankle vascular index and pulsatility index of the common carotid artery in patients with cardiovascular risk factors. J. Clin. Med. Res. 11, 593–599. 10.14740/jocmr3914 31413771 PMC6681855

[B25] Hodgkiss-Harlow K. D. Bandyk D. F. (2013). Interpretation of arterial duplex testing of lower-extremity arteries and interventions. SeminVasc. Surg. 26, 95–104. 10.1053/j.semvascsurg.2013.11.002 24636606

[B26] Hwang J. Y. (2017). Doppler ultrasonography of the lower extremity arteries: anatomy and scanning guidelines. Ultrasonography 36, 111–119. 10.14366/usg.16054 28219004 PMC5381852

[B27] Jani B. Rajkumar C. (2006). Ageing and vascular ageing. Postgrad. Med. J. 82, 357–362. 10.1136/pgmj.2005.036053 16754702 PMC2563742

[B28] Kaszczewski P. Elwertowski M. Leszczynski J. Ostrowski T. Galazka Z. (2020). Volumetric carotid flow characteristics in Doppler ultrasonography in healthy population over 65 years old. J. Clin. Med. 9:1375. 10.3390/jcm9051375 32392788 PMC7291321

[B29] Kochanowicz J. Turek G. Rutkowski R. Mariak Z. Szydlik P. Lyson T.. (2009). Normal reference values of ratios of blood flow velocities in internal carotid artery to those in common carotid artery using Doppler sonography. J. Clin. Ultrasound. 37, 208–211. 10.1002/jcu.20502 18561343

[B30] Kuhl V. Tettenborn B. Eicke B. M. Visbeck A. Meckes S. (2000). Color-coded duplex ultrasonography of the origin of the vertebral artery: normal values of flow velocities. J. Neuroimaging 10, 17–21. 10.1111/jon200010117 10666977

[B31] Laclaustra M. Casasnovas J. A. Fernández-Ortiz A. Fuster V. León-Latre M. Jiménez-Borreguero L. J.. (2016). Femoral and carotid subclinical atherosclerosis association with risk factors and coronary calcium: the AWHS Study. J. Am. Coll. Cardiol. 67, 1263–1274. 10.1016/j.jacc.2015.12.056 26988945

[B32] Lau K. K. Pego P. Mazzucco S. Li L. Howard D. P. Küker W.. (2018). Age and sex-specific associations of carotid pulsatility with small vessel disease burden in transient ischemic attack and ischemic stroke. Int. J. Stroke 13, 832–839. 10.1177/1747493018784448 29966494 PMC6424409

[B33] Lumley T. Diehr P. Emerson S. Chen L. (2002). The importance of the normality assumption in large public health data sets. Annu. Rev. Public Health 23, 151–169. 10.1146/annurev.publhealth.23.100901.140546 11910059

[B34] Marin M. Bia D. Zócalo Y. (2020). Carotid and femoral atherosclerotic plaques in asymptomatic and non-treated subjects: cardiovascular risk factors, 10-years risk scores, and lipid ratios' capability to detect plaque presence, burden, fibro-lipid composition and geometry. J. Cardiovasc. Dev. Dis. 7:11. 10.3390/jcdd7010011 32204546 PMC7151111

[B35] Naghavi M. Falk E. Hecht H. S. Jamieson M. J. Kaul S. Berman D.. (2006). From vulnerable plaque to vulnerable patient—Part III: Executive summary of the Screening for Heart Attack Prevention and Education (SHAPE) Task Force report. Am. J. Cardiol. 98, 2H−15H. 10.1016/j.amjcard.2006.03.002 16843744

[B36] Nemati M. Bavil A. S. Taheri N. (2009). Comparison of normal values of Duplex indices of ver-tebral arteries in young and elderly adults. Cardiovasc. Ultrasound. 7:2. 10.1186/1476-7120-7-2 19138434 PMC2640361

[B37] Oates C. P. Naylor A. R. Hartshorne T. Charles S. M. Fail T. Humphries K.. (2009). Joint recommendations for reporting carotid ultrasound investigations in the United Kingdom. Eur. J. Vasc. Endovasc. Surg. 37, 251–261. 10.1016/j.ejvs.2008.10.015 19046904

[B38] Oglat A. A. Matjafri M. Z. Suardi N. Oqlat M. A. Abdelrahman M. A. Oqlat A. A. (2018). A review of medical Doppler ultrasonography of blood flow in general and especially in common carotid artery. J. Med. Ultrasound. 26, 3–13. 10.4103/JMU.JMU_11_17 30065507 PMC6029191

[B39] Royston P. Wright E. (1998). A method for estimating age-specific reference intervals (‘normal ranges’) based on fractional polynomials and exponential transformation. J. R. Stat. Soc. Ser. A Stat. Soc. 161, 79–101. 10.1111/1467-985X.00091

[B40] Santana D. B. Zócalo Y. A. Armentano R. L. (2012a). Integrated e-Health approach based on vascular ultrasound and pulse wave analysis for asymptomatic atherosclerosis detection and cardiovascular risk stratification in the community. IEEE Trans. Inf. Technol. Biomed. 16, 287–294. 10.1109/TITB.2011.2169977 22271835

[B41] Santana D. B. Zócalo Y. A. Ventura I. F. Arrosa J. F. Florio L. Lluberas R.. (2012b). Health informatics design for assisted diagnosis of subclinical atherosclerosis, structural, and functional arterial age calculus and patient-specific cardiovascular risk evaluation. IEEE Trans. Inf. Technol. Biomed. 16, 943–951. 10.1109/TITB.2012.2190990 22434819

[B42] Santos S. N. D. Alcantara M. L. Freire C. M. V. Cantisano A. L. Teodoro J. A. R. Porto C. L. L.. (2019). Vascular ultrasound statement from the department of cardiovascular imaging of the Brazilian Society of Cardiology-−2019. Arq. Bras. Cardiol. 112, 809–849. 10.5935/abc.20190106 31314836 PMC6636370

[B43] Scheel P. Ruge C. Schöning M. (2000). Flow velocity and flow volume measurements in the extracranial carotid and vertebral arteries in healthy adults: reference data and the effects of age. Ultrasound. Med. Biol. 26, 1261–1266. 10.1016/S0301-5629(00)00293-3 11120363

[B44] Schoning M. Hartig B. (1998). The development of hemodynamics in the extracranial carotid and vertebral arteries. Ultrasound. Med. Biol. 24, 655–662. 10.1016/S0301-5629(98)00029-5 9695268

[B45] Seidel E. Eicke B. M. Tettenborn B. Krummenauer F. (1999). Reference values for vertebral artery flow volume by duplex sonography in young and elderly adults. Stroke 30, 2692–2696. 10.1161/01.STR.30.12.2692 10582999

[B46] Shaalan W. E. French-Sherry E. Castilla M. Lozanski L. Bassiouny H. S. (2003). Reliability of common femoral artery hemodynamics in assessing the severity of aortoiliac inflow disease. J. Vasc. Surg. 37, 960–969. 10.1067/mva.2003.282 12756340

[B47] Sosnowski C. Pasierski T. Janeczko-Sosnowska E. Szulczyk A. Dabrowski R. Wozniak J.. (2007). Femoral rather than carotid artery ultrasound imaging predicts extent and severity of coronary artery disease. Kardiol. Pol. 65, 760–766; discussion 767–8. Available online at: https://journals.viamedica.pl/kardiologia_polska/article/view/80839 17694457

[B48] Tokunaga K. Koga M. Yoshimura S. Arihiro S. Suzuki R. Nagatsuka K.. (2016). Optimal peak systolic velocity thresholds for predicting internal carotid artery stenosis greater than or equal to 50%, 60%, 70%, and 80%. J. Stroke Cerebrovasc. Dis. 25, 921–926. 10.1016/j.jstrokecerebrovasdis.2015.12.021 26851210

[B49] Trihan J. E. Perez-Martin A. Guillaumat J. Lanéelle D. (2020). Normative and pathological values of hemodynamic and Doppler ultrasound arterial findings in children. Vasa 49, 264–274. 10.1024/0301-1526/a000860 32323630

[B50] Yazici B. Erdogmuş B. Tugay A. (2005). Cerebral blood flow measurements of the extracranial carotid and vertebral arteries with Doppler ultrasonography in healthy adults. Diagn. Interv. Radiol. 11, 195–198. Available online at: https://www.dirjournal.org/en/cerebral-blood-flow-measurements-of-the-extracranial-carotid-and-vertebral-arteries-with-doppler-ultrasonography-in-healthy-adults-1377 16320223

[B51] Zbornikova V. Lassvik C. (1986). Duplex scanning in presumably normal persons of different ages. Ultrasound. Med. Biol. 12, 371–378. 10.1016/0301-5629(86)90194-8 3521027

[B52] Zócalo Y. Bia D. (2016). Ultrasonografía carotídea para detección de placas de ateroma y medición del espesor íntima-media; índice tobillo-brazo: Evaluación no invasivaen la práctica clínica: Importancia clínica y análisis de las bases metodológicas para su evaluación. Rev. Urug. Cardiol. 31, 47–60. Available online at: http://www.scielo.edu.uy/scielo.php?script=sci_arttext&pid=S1688-04202016000100012

[B53] Zócalo Y. Bia D. (2021). Age- and sex-related profiles for macro, macro/micro and microvascular reactivity indexes: association between indexes and normative data from 2609 healthy subjects (3–85 years). PloS ONE 16:e0254869. 10.1371/journal.pone.0254869 34280235 PMC8289111

[B54] Zócalo Y. Curcio S. García-Espinosa V. Chiesa P. Giachetto G. Bia D. (2017). Comparative analysis of arterial parameters variations associated with inter-individual variations in peripheral and aortic blood pressure: cross-sectional study in healthy subjects aged 2–84 years. High Blood Press Cardiovasc. Prev. 24, 437–451. 10.1007/s40292-017-0231-2 28948506

[B55] Zócalo Y. García-Espinosa V. Castro J. M. Zinoveev A. Marin M. Chiesa P.. (2020). Stroke volume and cardiac output non-invasive monitoring based on brachial oscillometry-derived pulse contour analysis: explanatory variables and reference intervals throughout life (3–88 years). Cardiol. J. 10.5603/CJ.a2020.0031 32207845 PMC8747806

