# Supplementary material for: Sex- and Age-Related Physiological Profiles for Brachial, Vertebral, Carotid, and Femoral Arteries Blood Flow Velocity Parameters During Growth and Aging (4–76 Years): Comparison With Clinical Cut-Off Levels
Source: Front Physiol. 2021 Aug 26;12:729309. doi: 10.3389/fphys.2021.729309 (PMC8427671; doi:10.3389/fphys.2021.729309)
Supplement: Supplementary file 2 [file Table_2.DOCX]

**Supplementary Figures: Common Carotid Artery (CCA)**


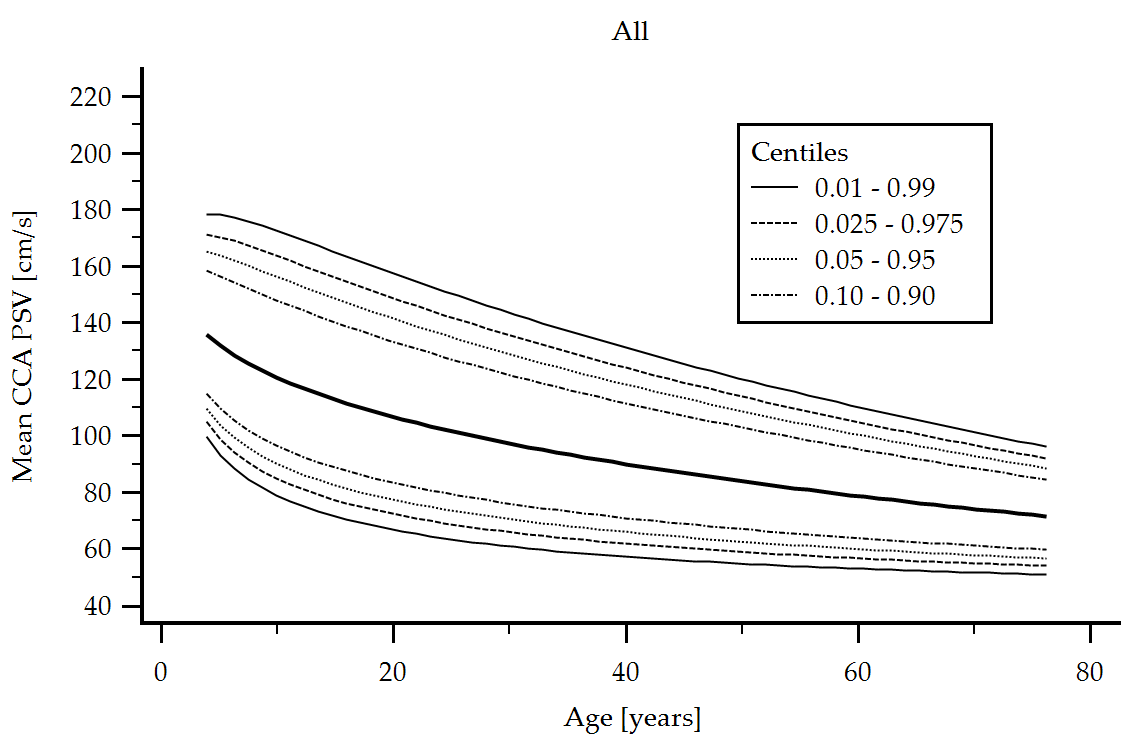


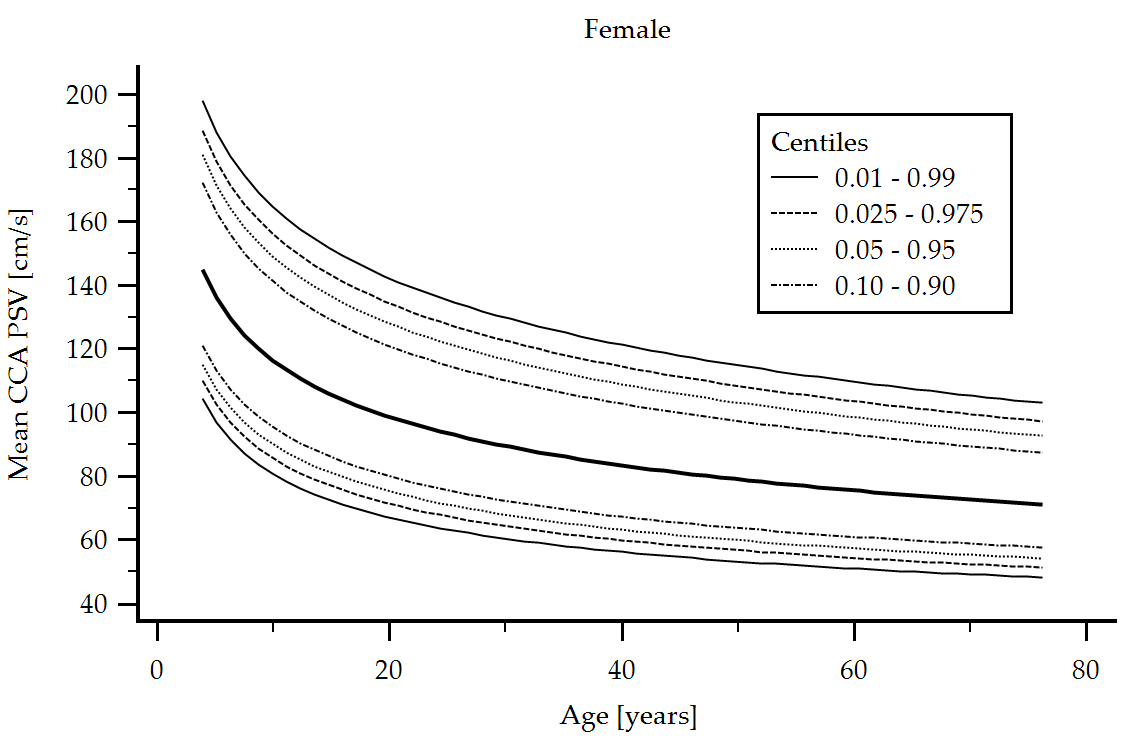

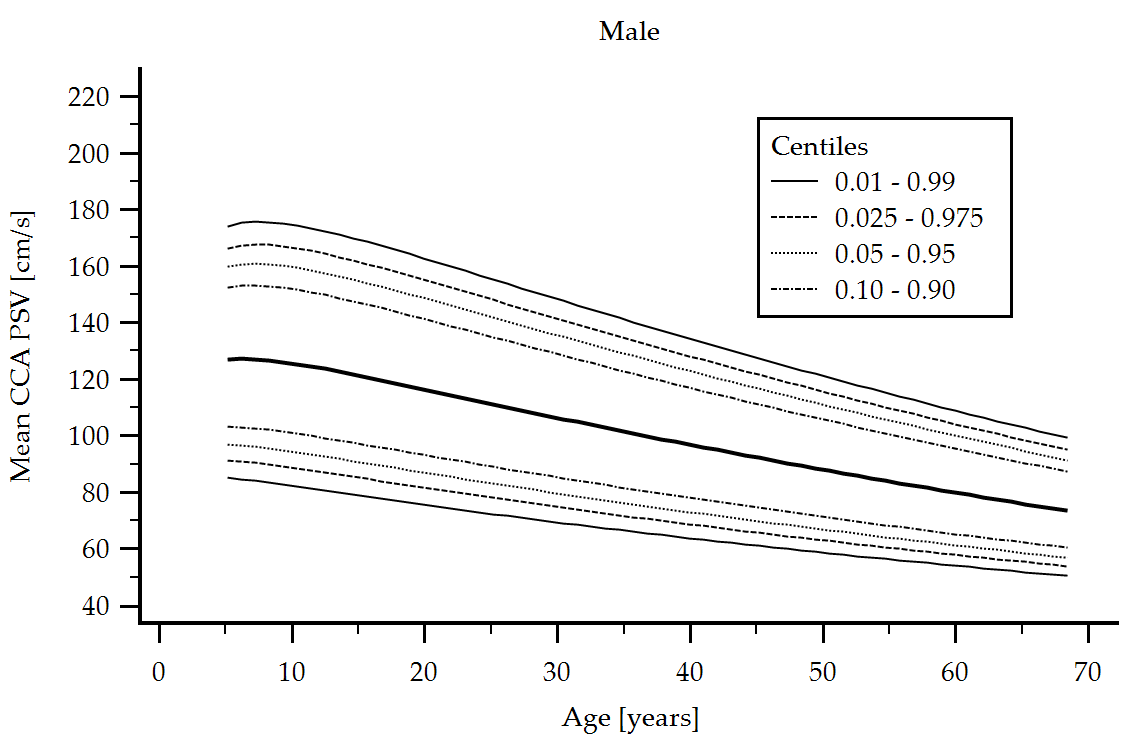


Supplementary Figure 1. Common Carotid Artery (CCA) blood flow velocity percentile curves for all, females and males. PSV: peak systolic velocity. Mean value for left and right CCA.


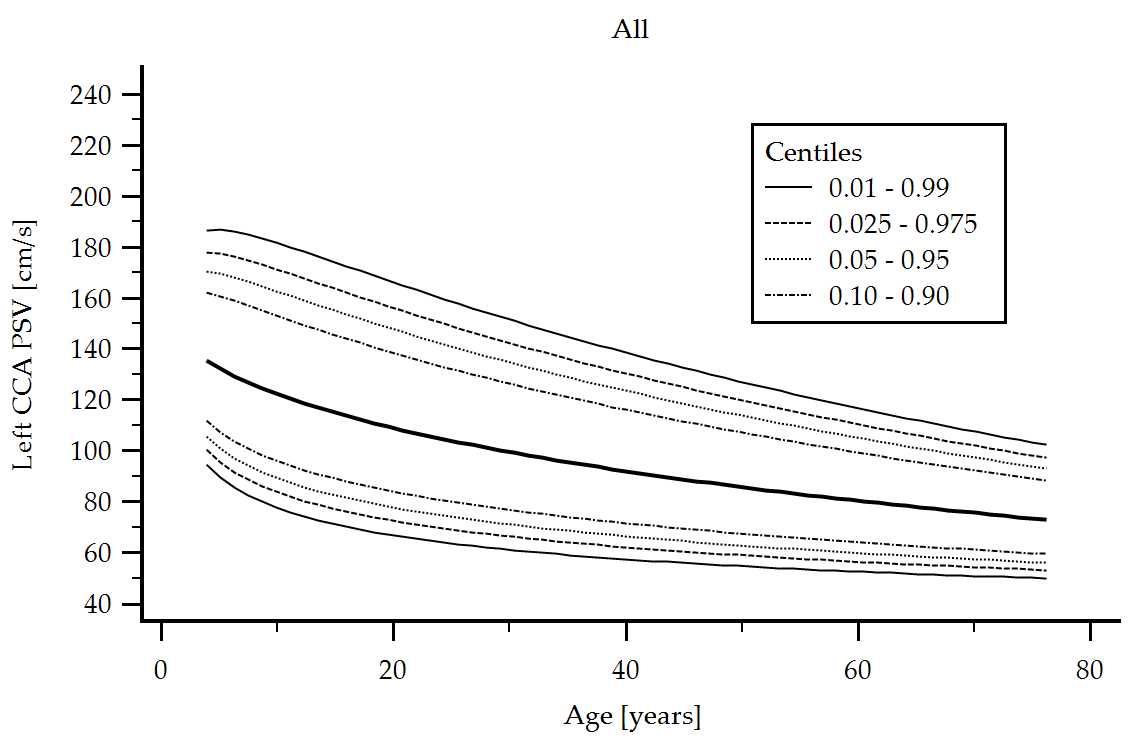


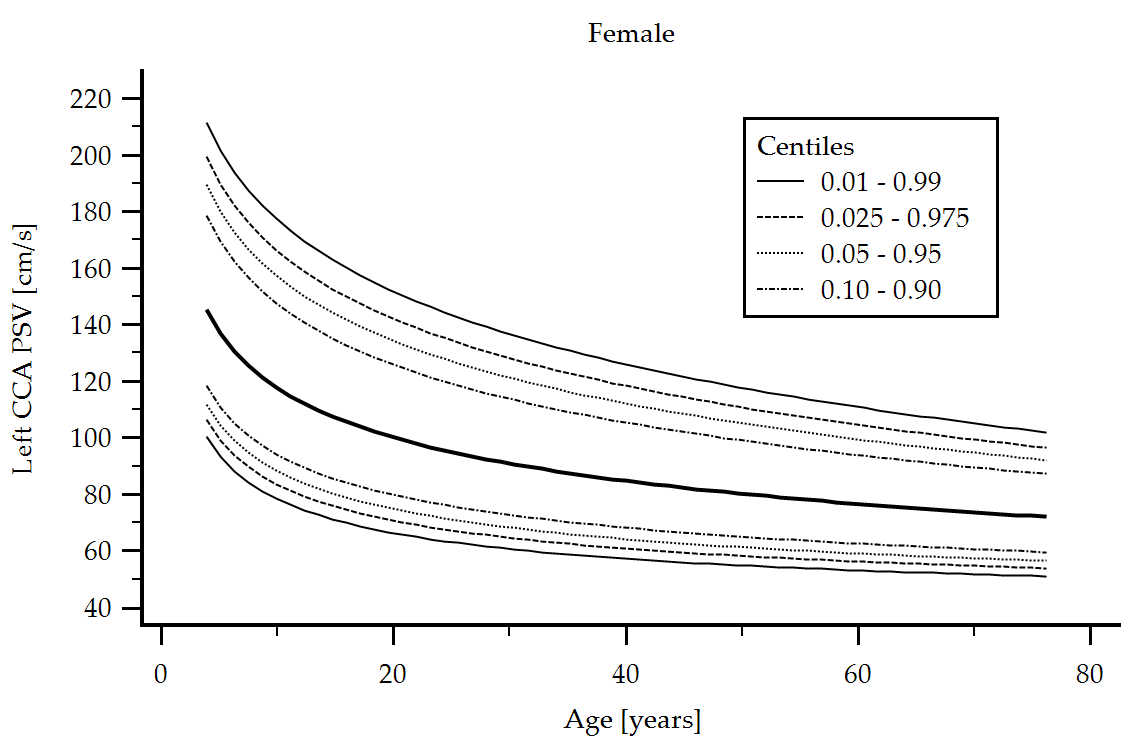


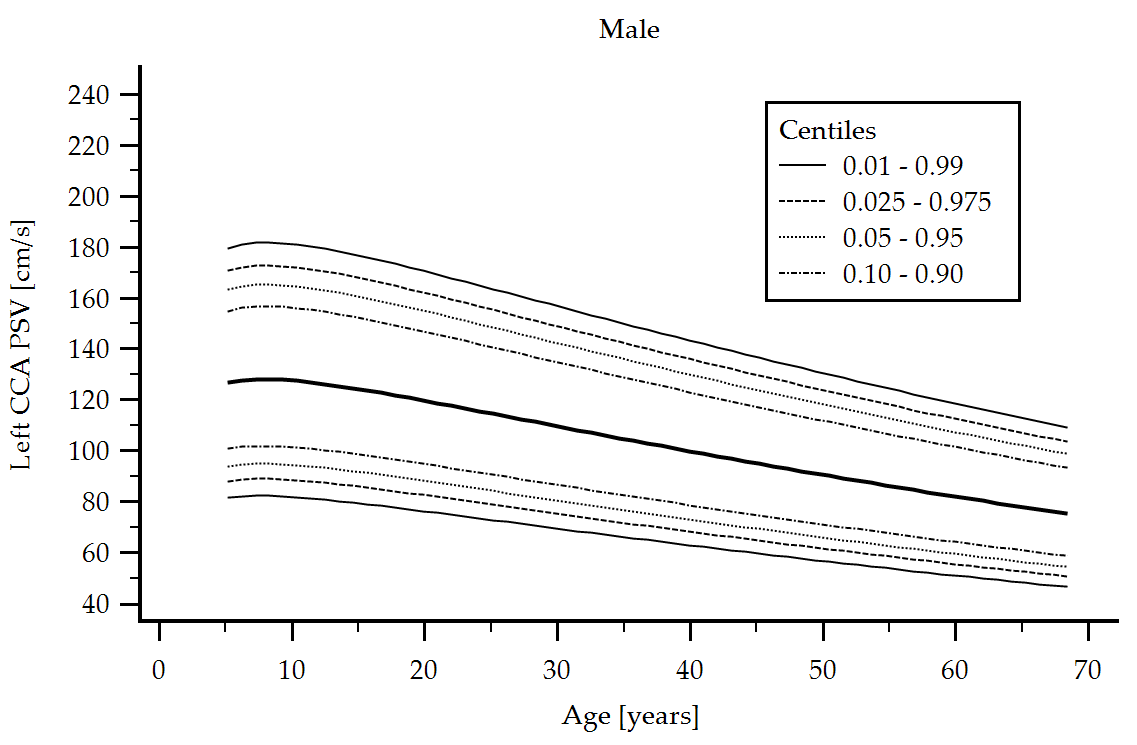


Supplementary Figure 2. Lef Common Carotid Artery (CCA) blood flow velocity percentile curves for all, females and males. PSV: peak systolic velocity.


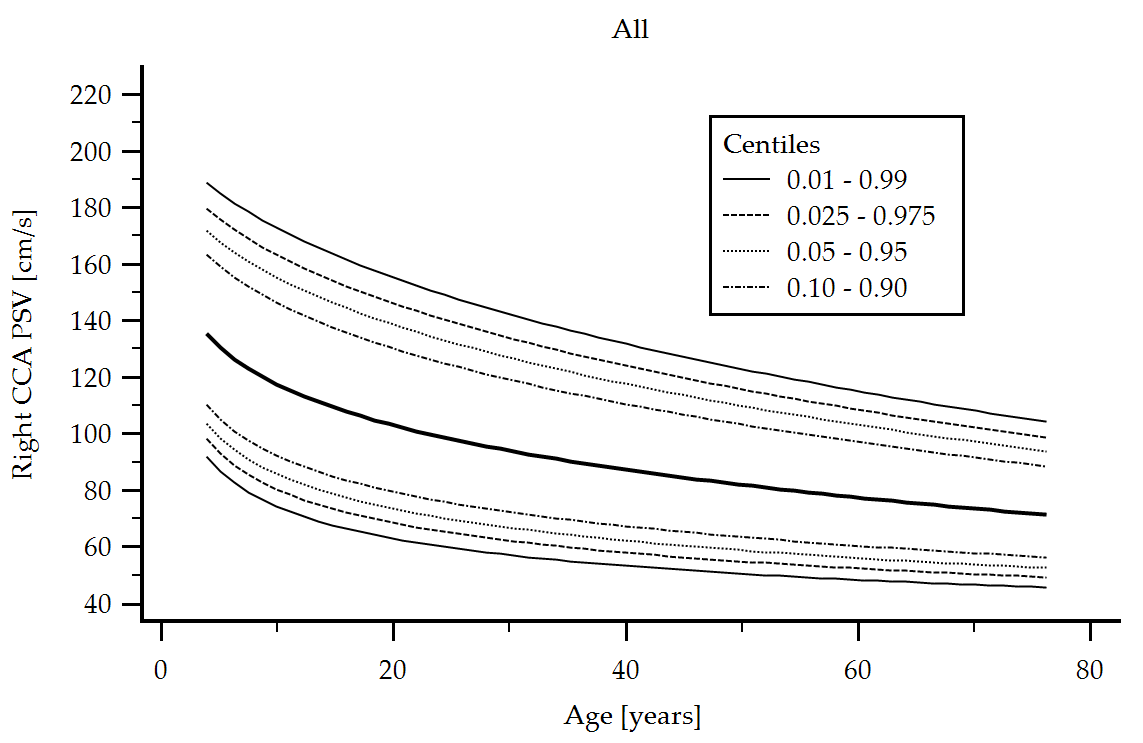


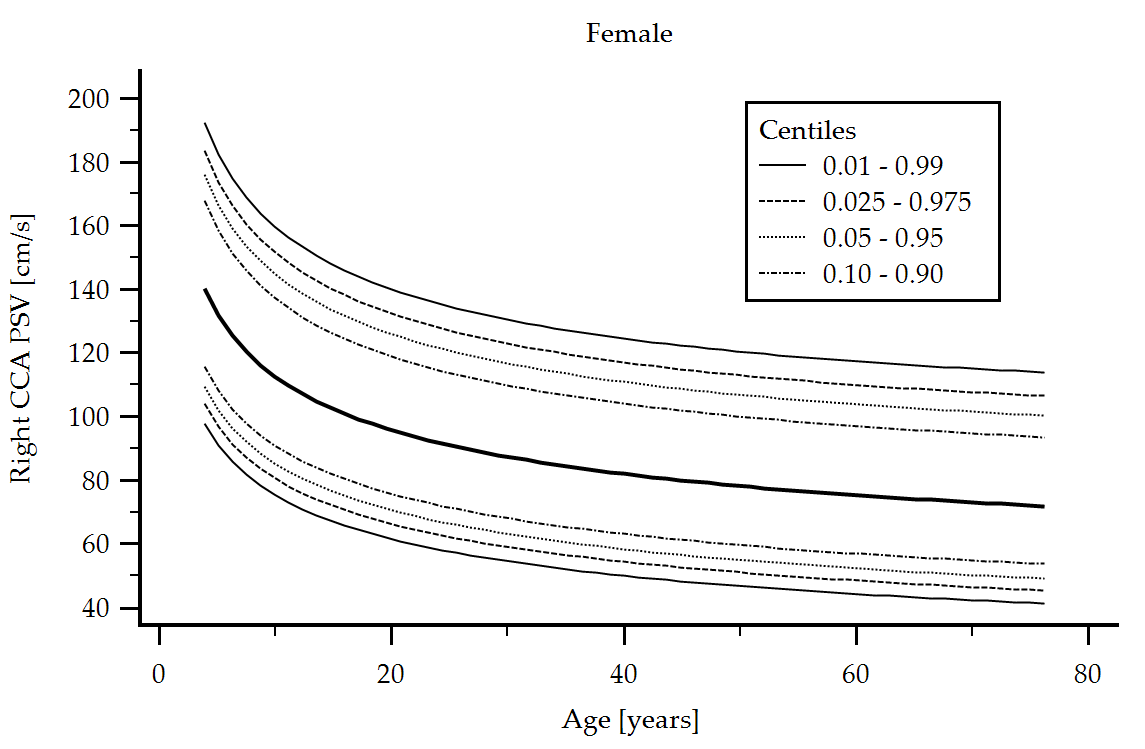

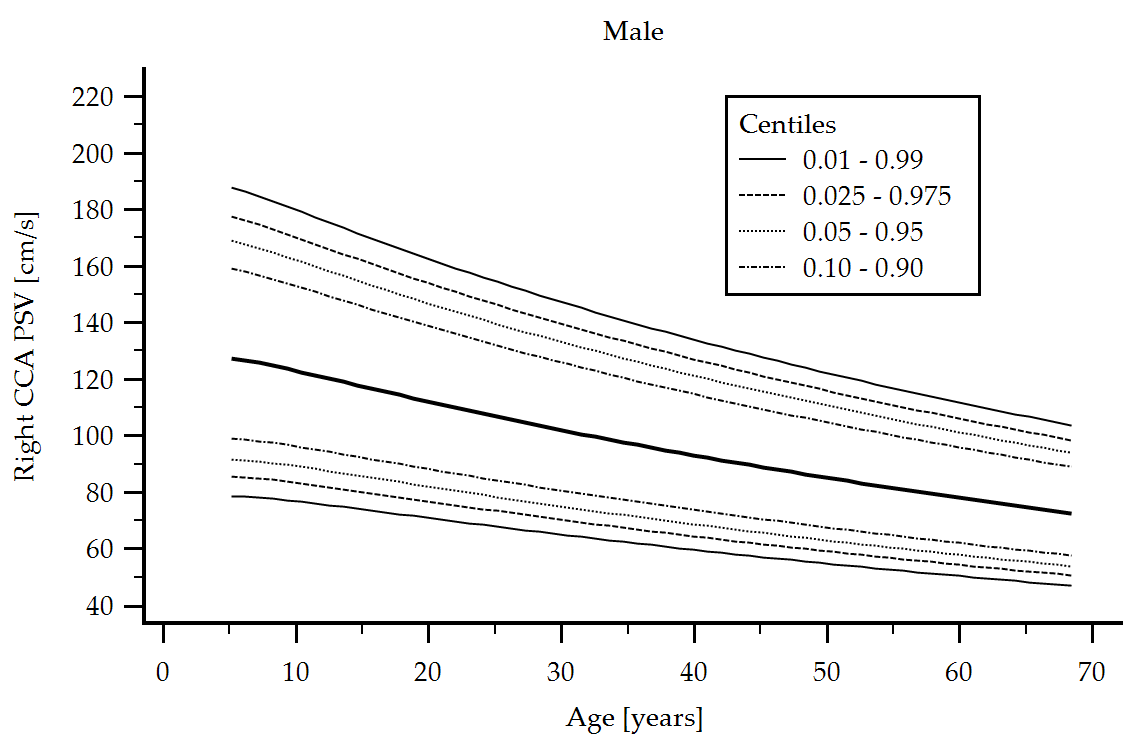


Supplementary Figure 3. Right Common Carotid Artery (CCA) blood flow velocity percentile curves for all, females and males. PSV: peak systolic velocity.


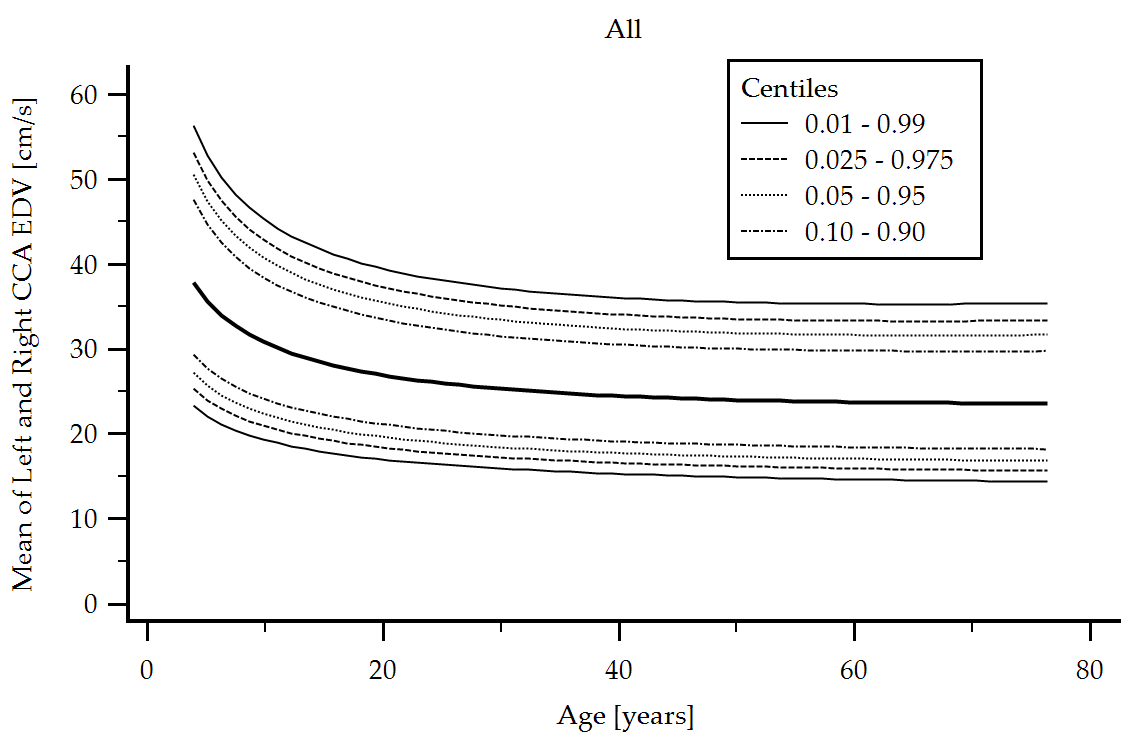

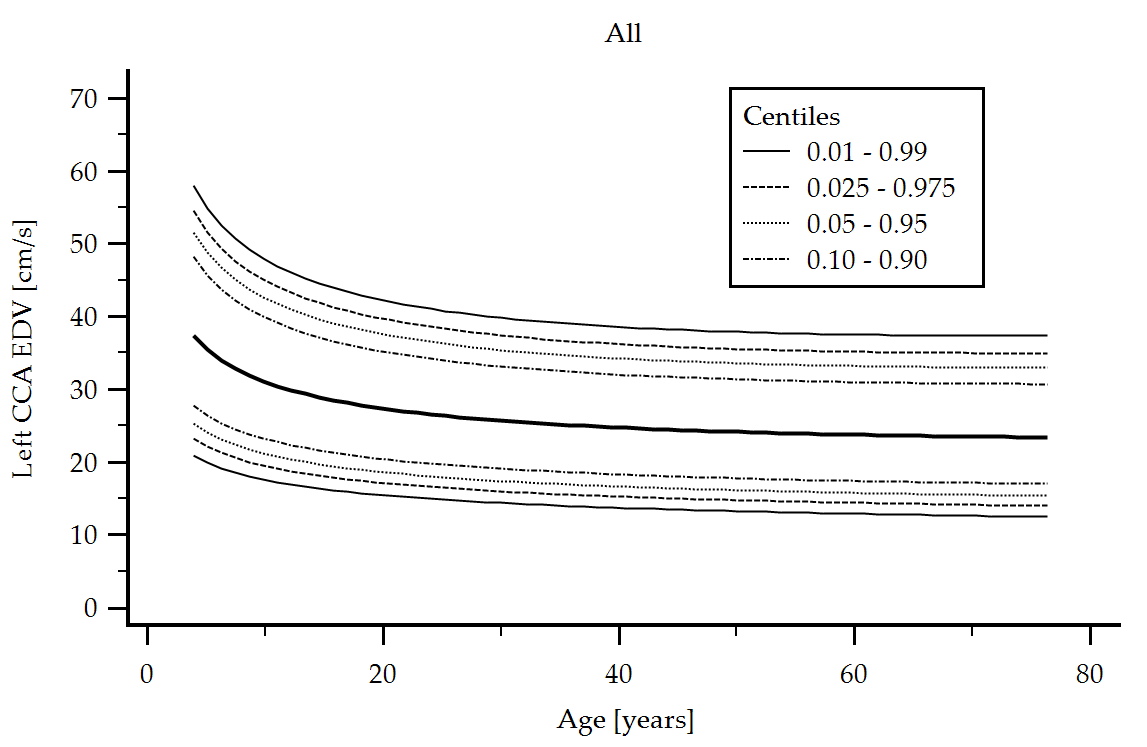

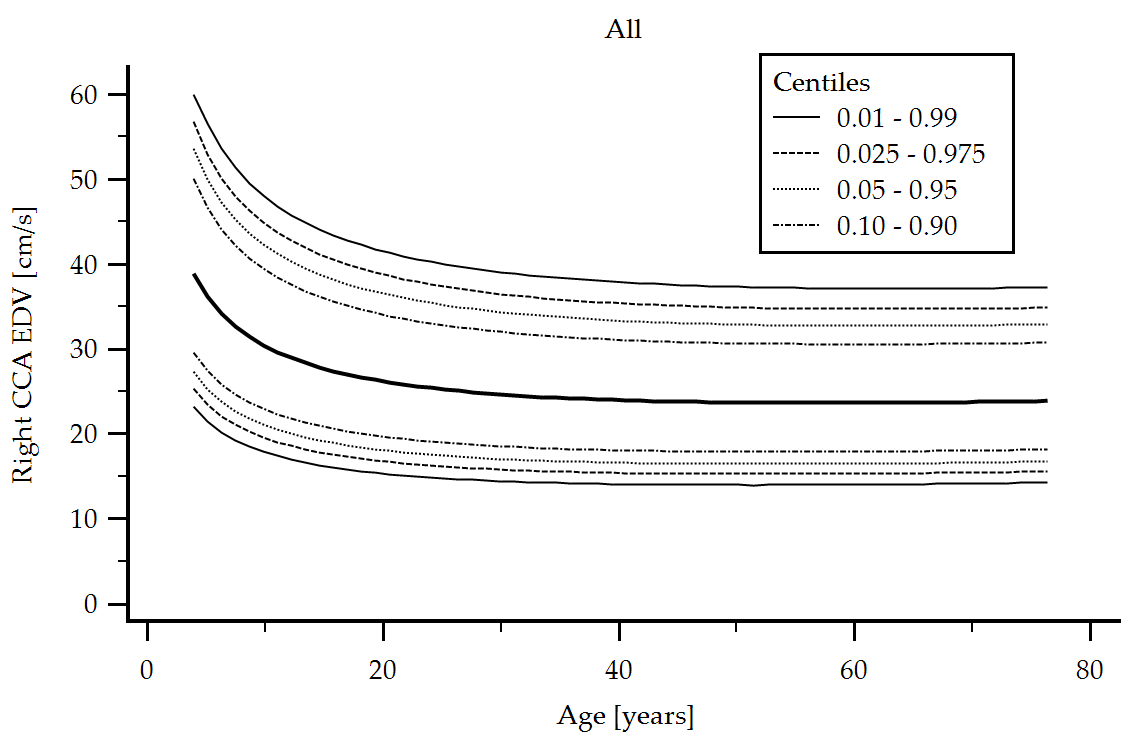


Supplementary Figure 4. Common Carotid Artery (CCA) blood flow velocity percentile curves for all, females and males. EDV: end-diastolic velocity.


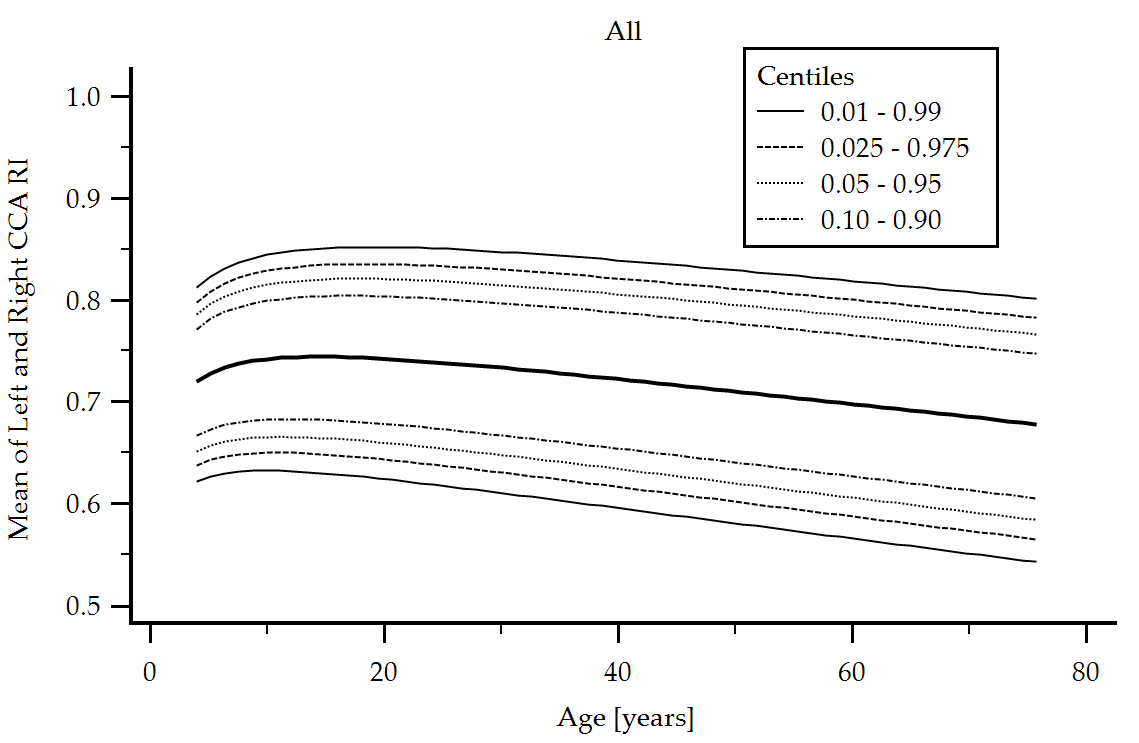

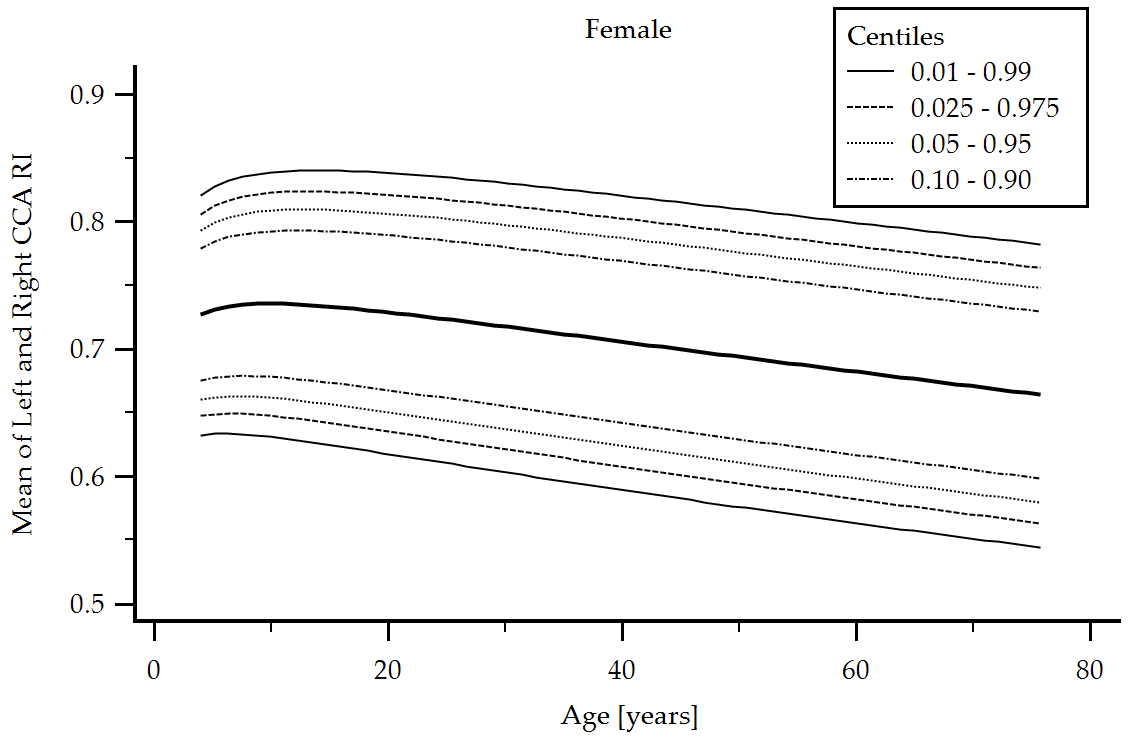

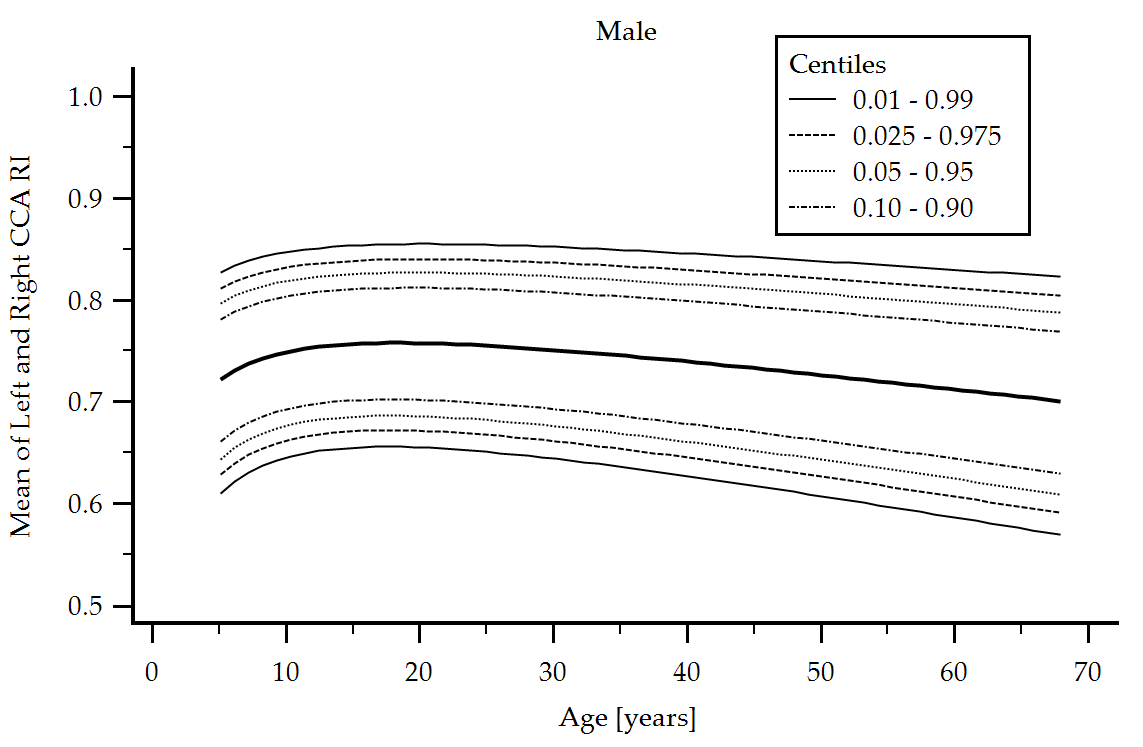


Supplementary Figure 5. Common Carotid Artery (CCA) blood flow velocity percentile curves for all, females and males. RI: resistive index.


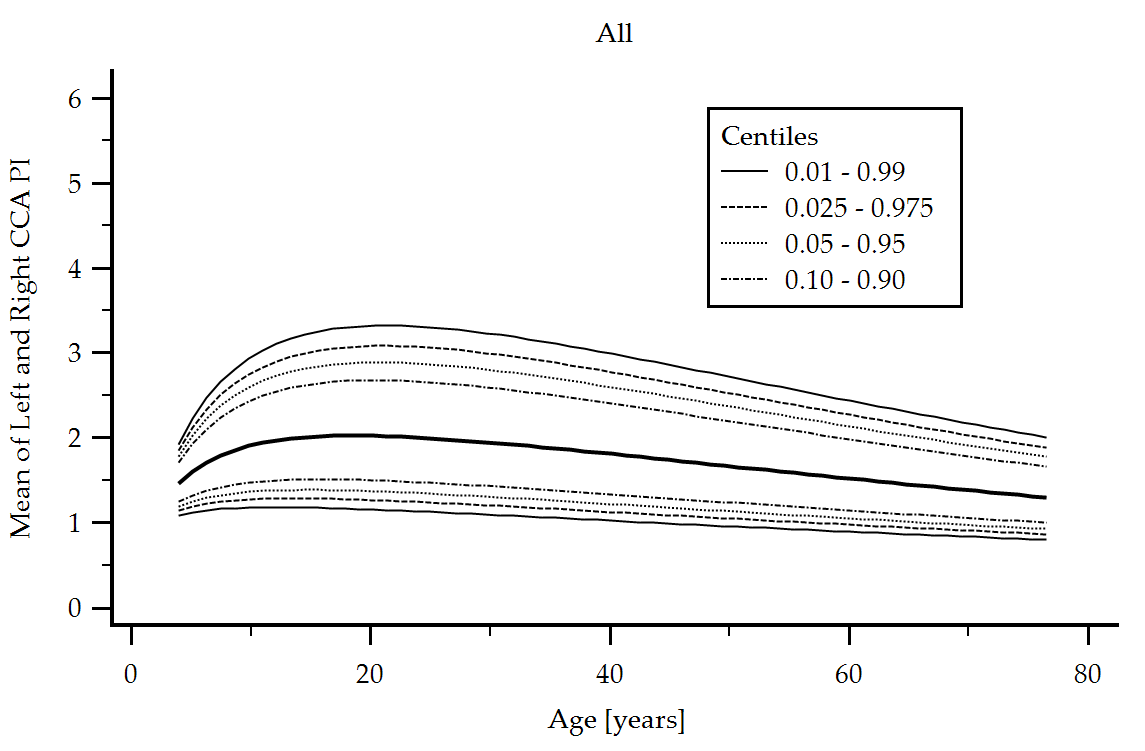

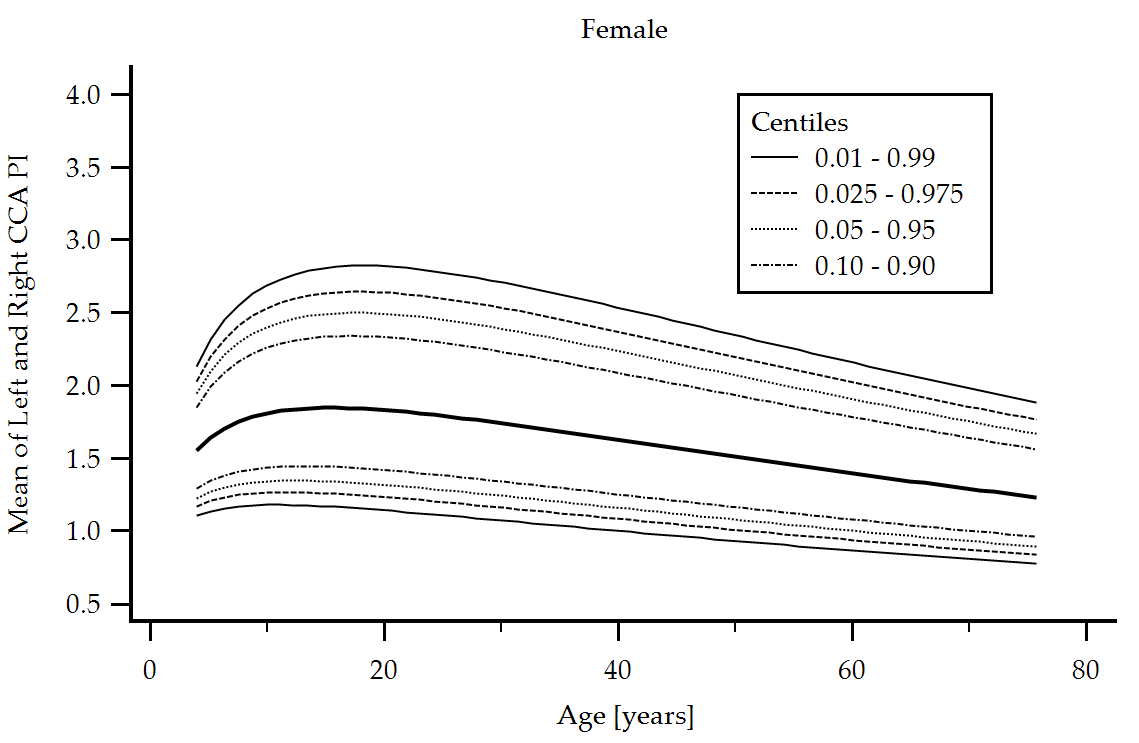

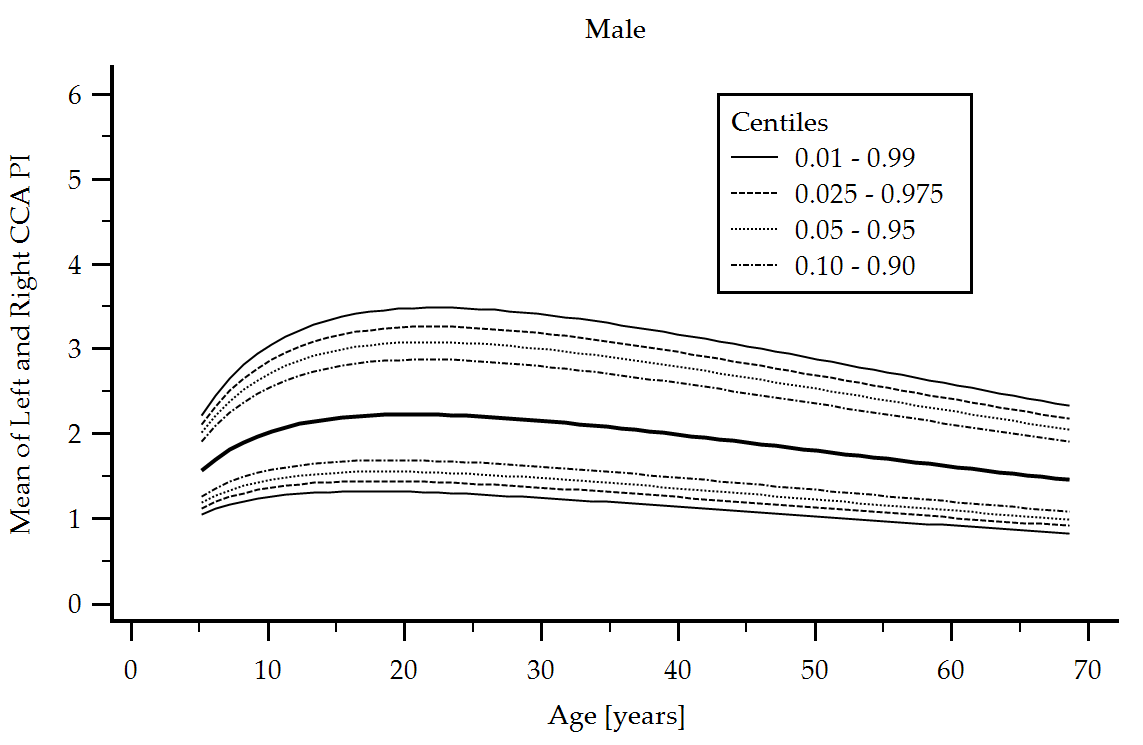


Supplementary Figure 6. Common Carotid Artery (CCA) blood flow velocity percentile curves for all, females and males. PI: pulsatile index.


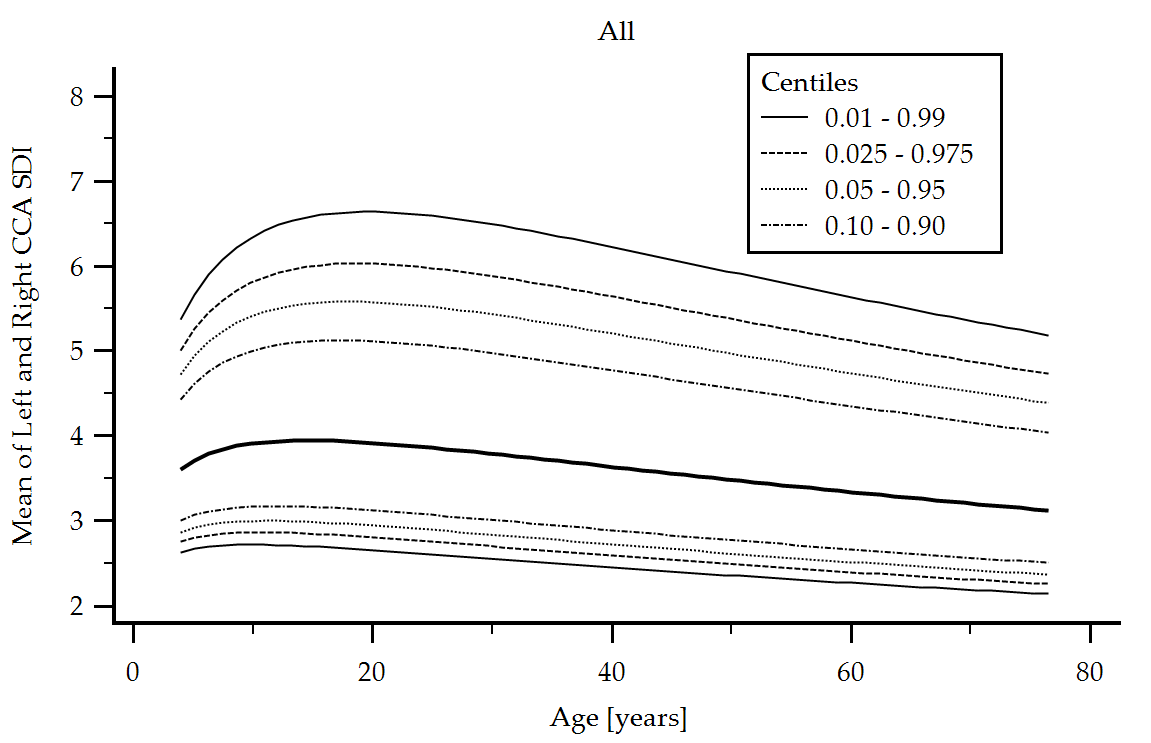

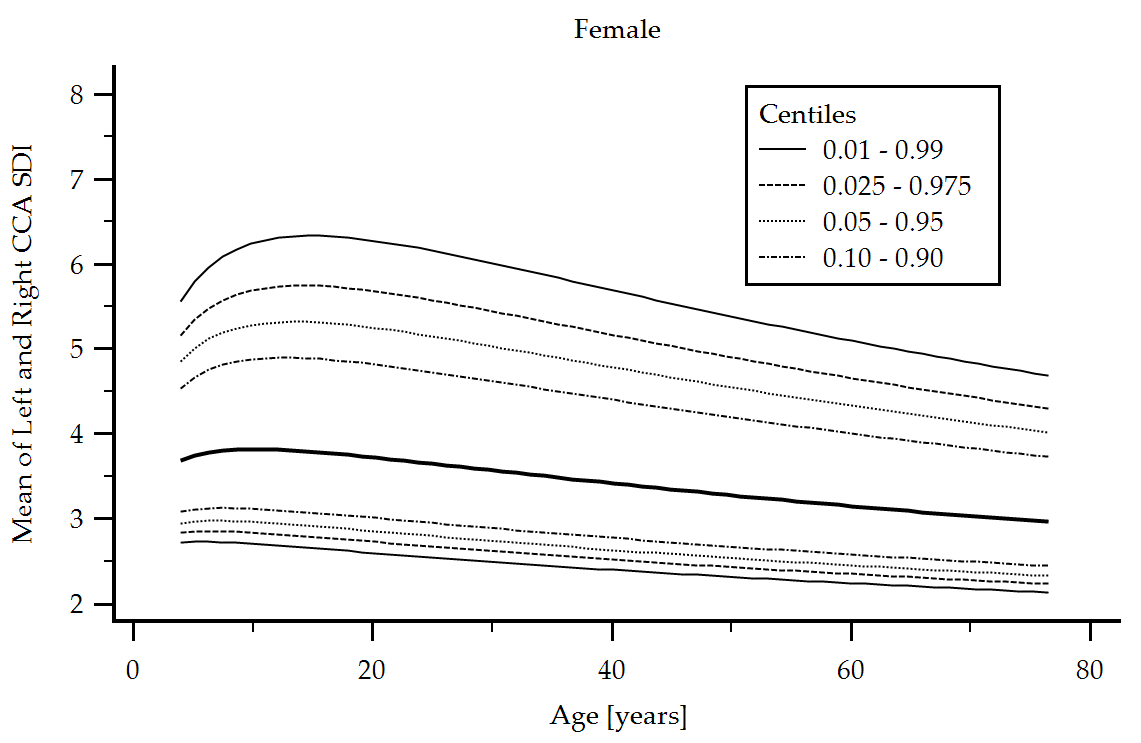

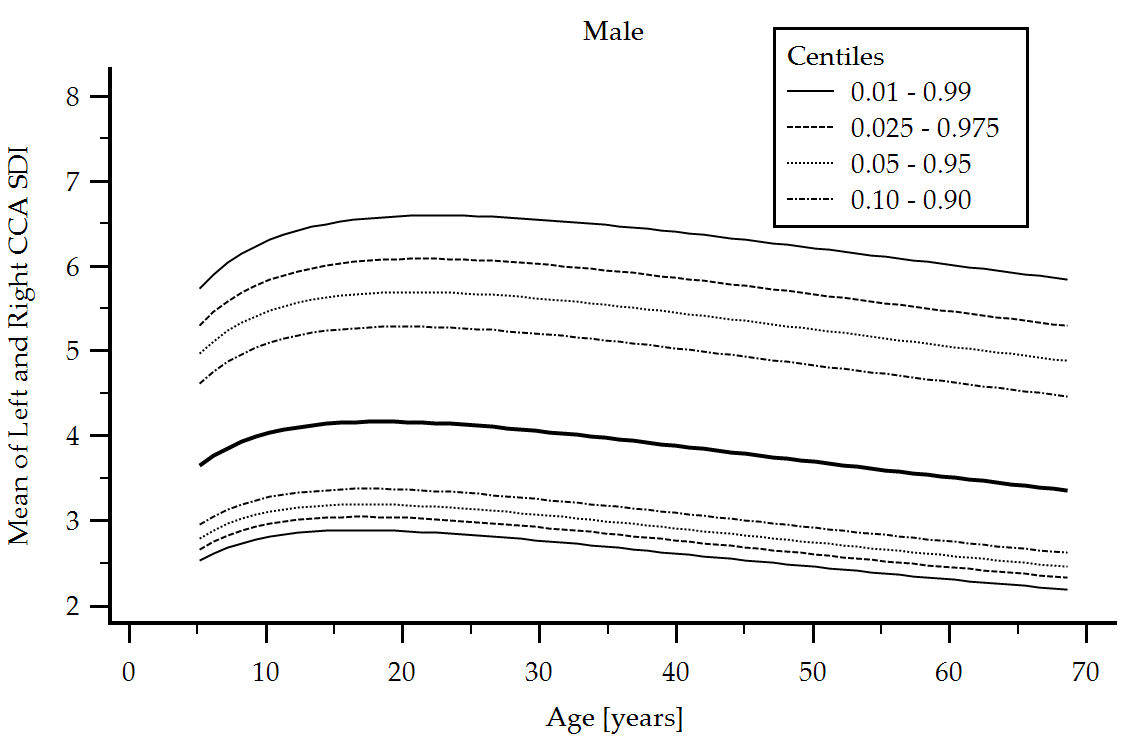


Supplementary Figure 7. Common Carotid Artery (CCA) blood flow velocity percentile curves for all, females and males. SDI: systo-diastolic index.

**Supplementary Figures: Internal Carotid Artery (ICA)**

**
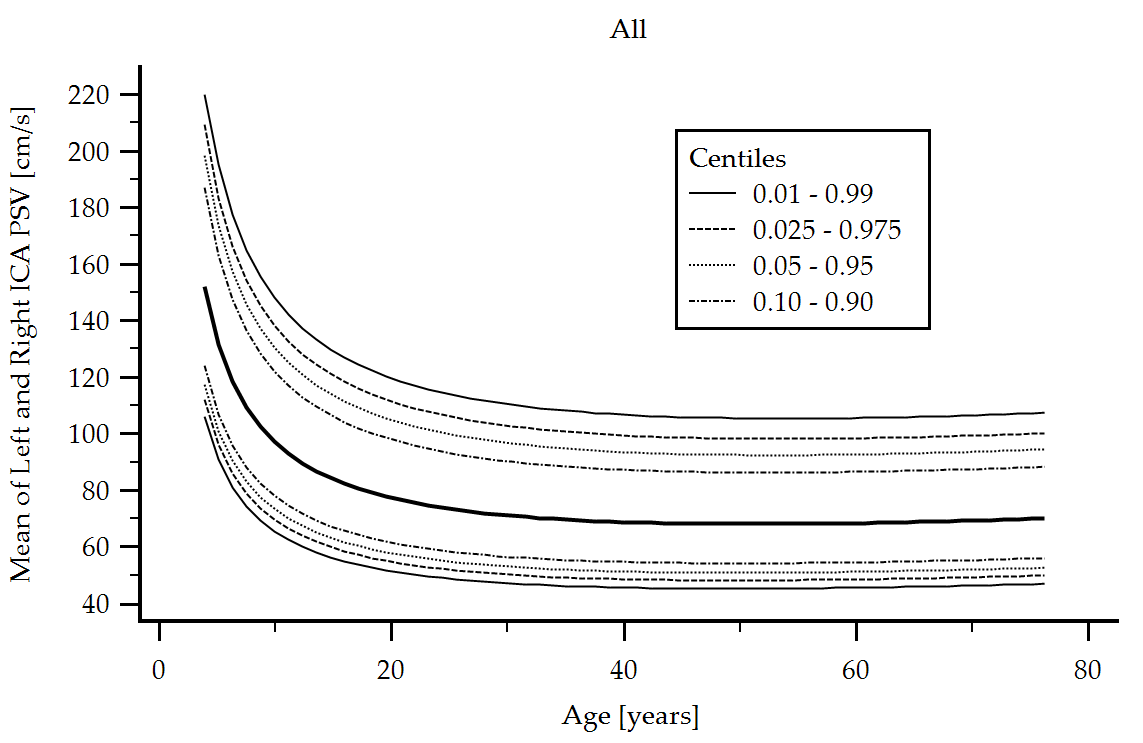

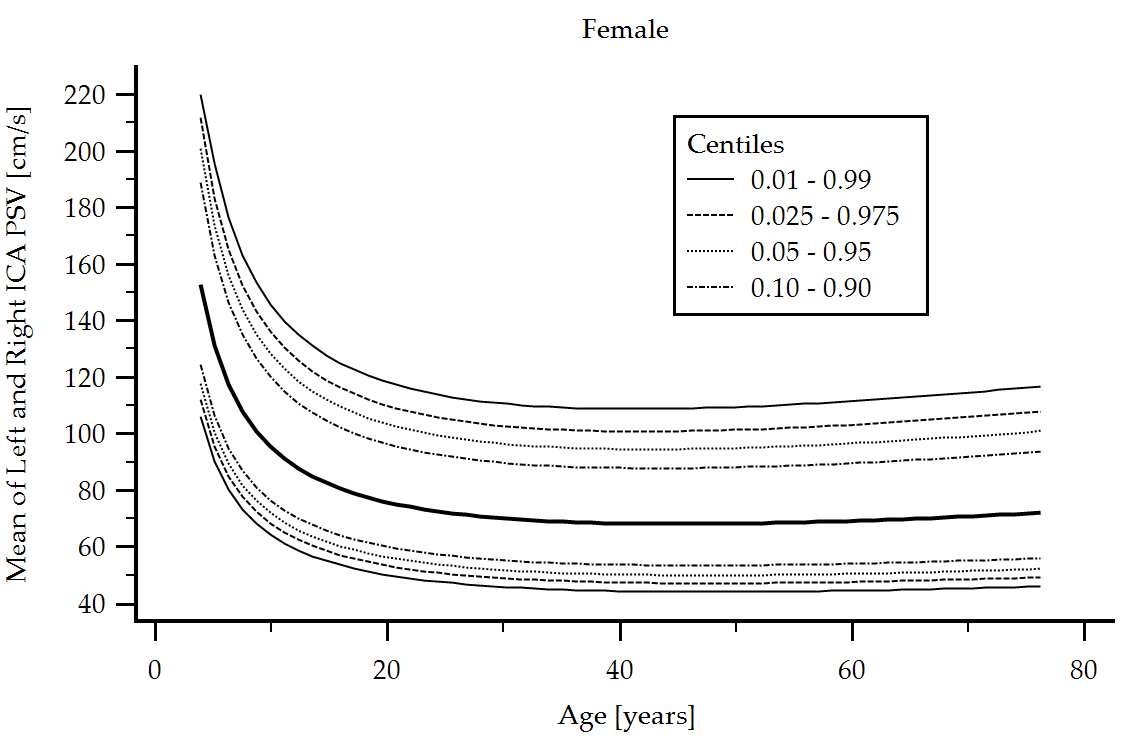

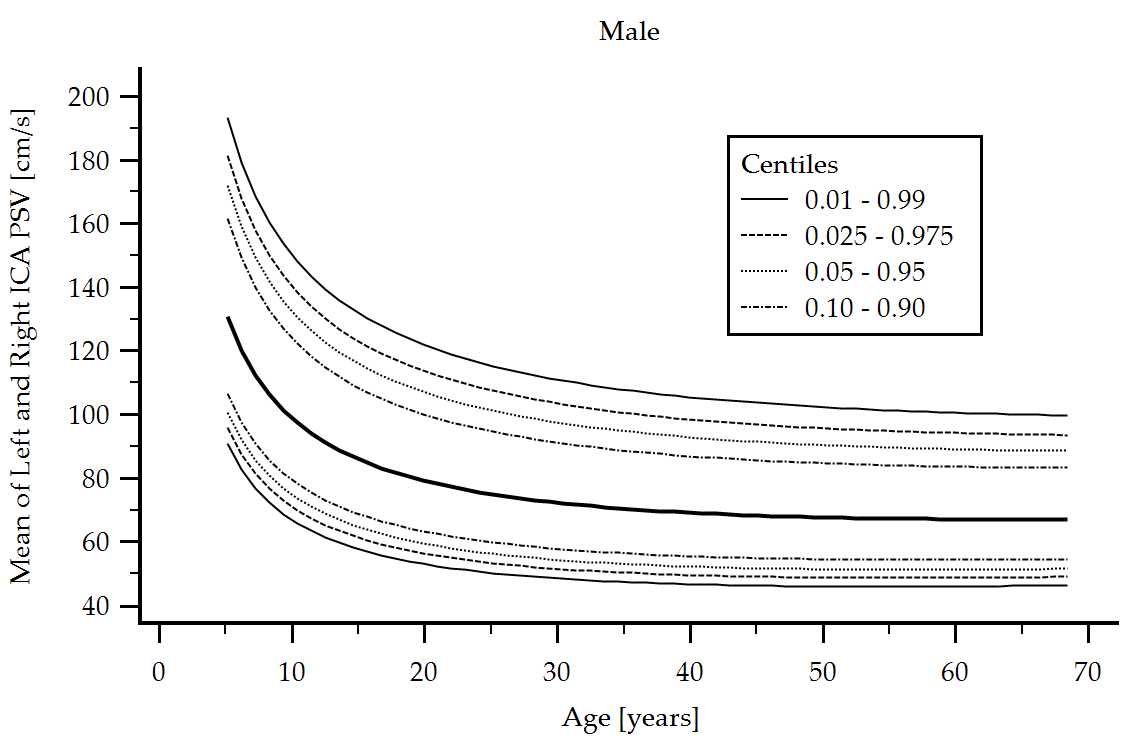
**

Supplementary Figure 8. Internal Carotid Artery (ICA) blood flow velocity percentile curves for all, females and males. PSV: peak systolic velocity.


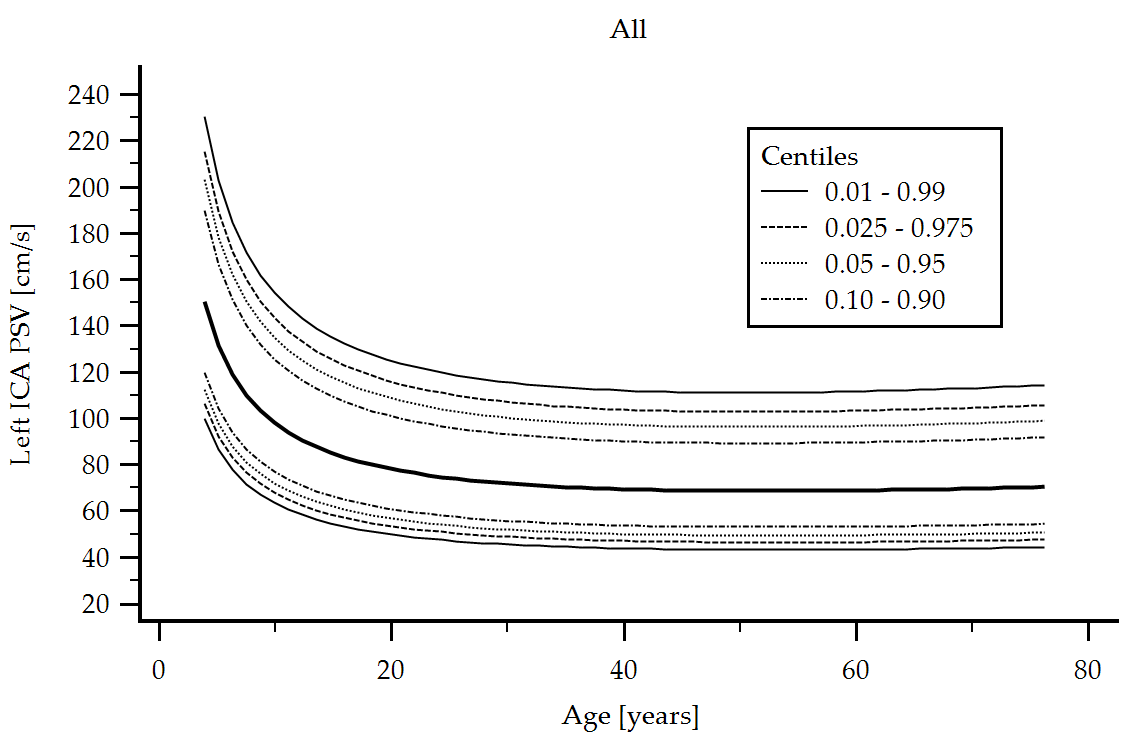

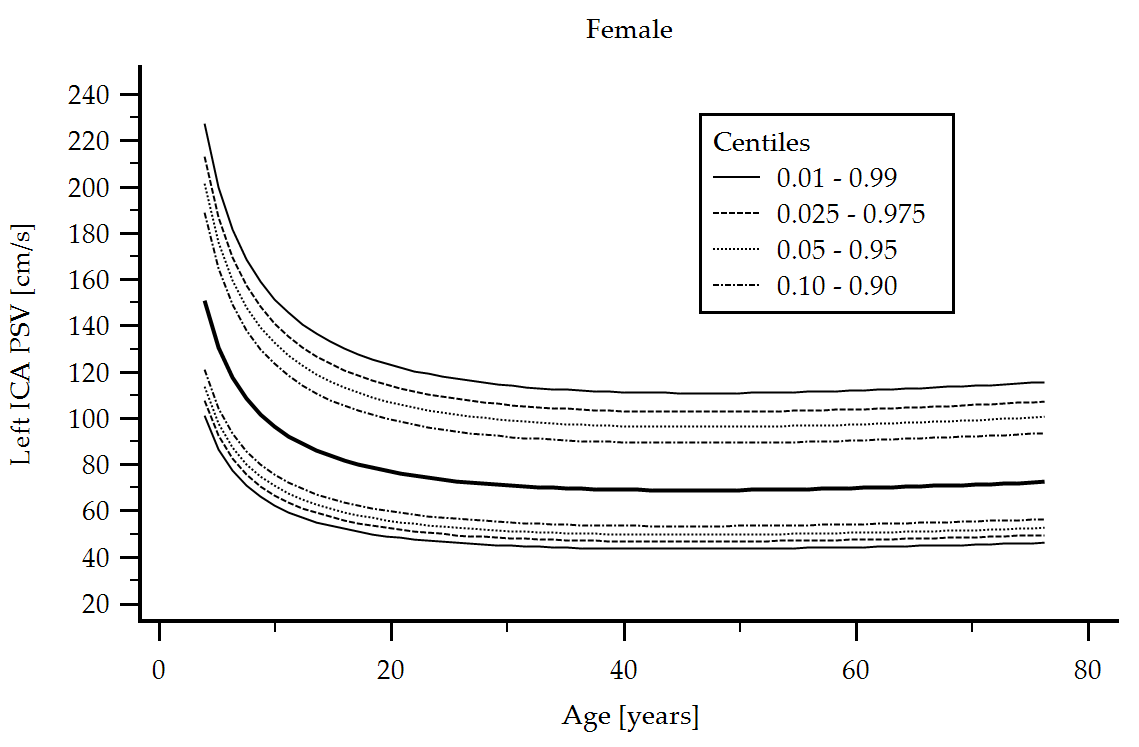

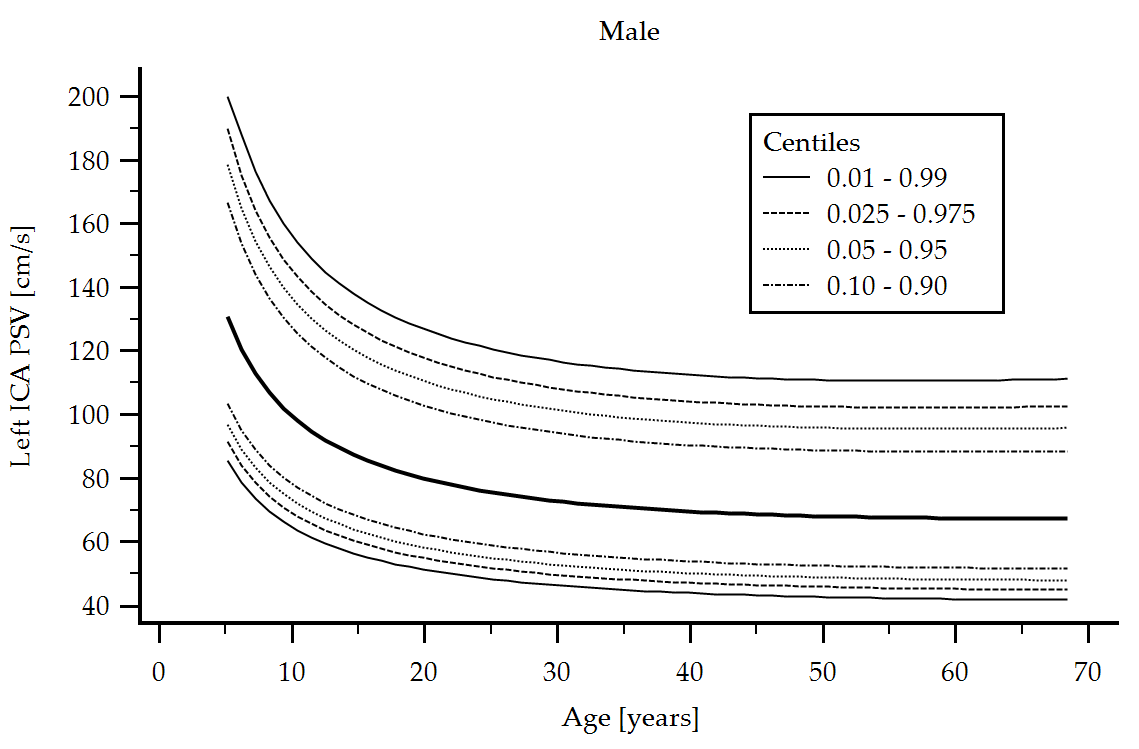


Supplementary Figure 9. Left Internal Carotid Artery (ICA) blood flow velocity percentile curves for all, females and males. PSV: peak systolic velocity.


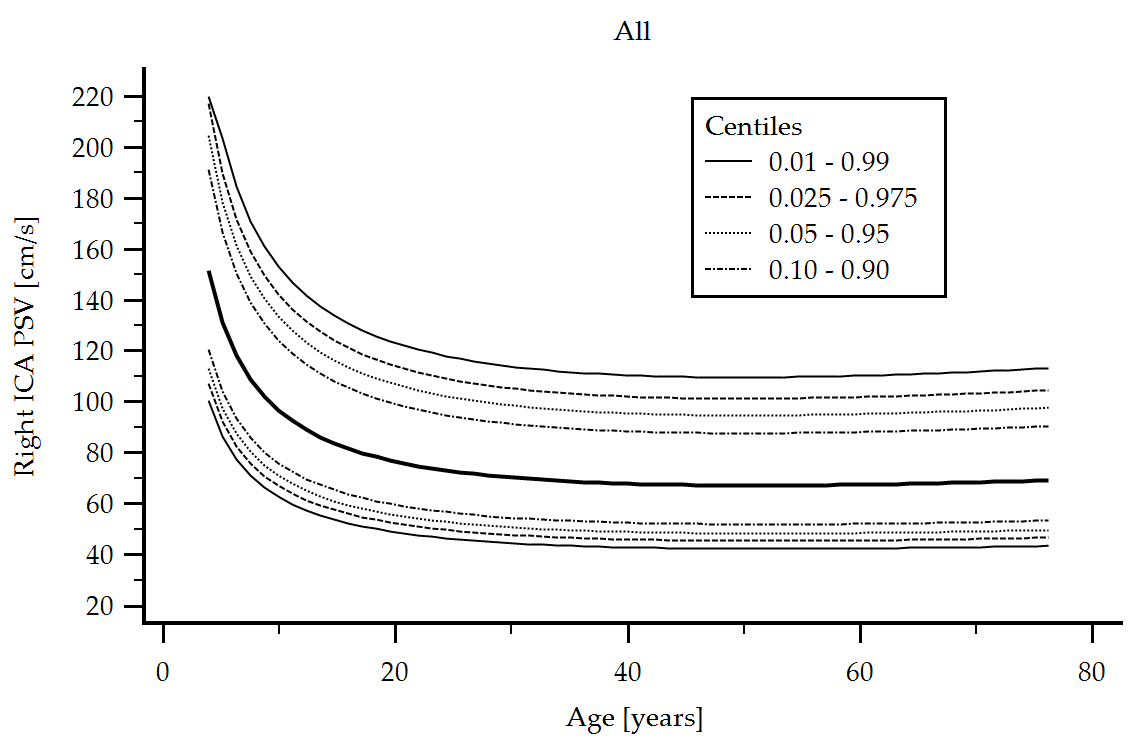

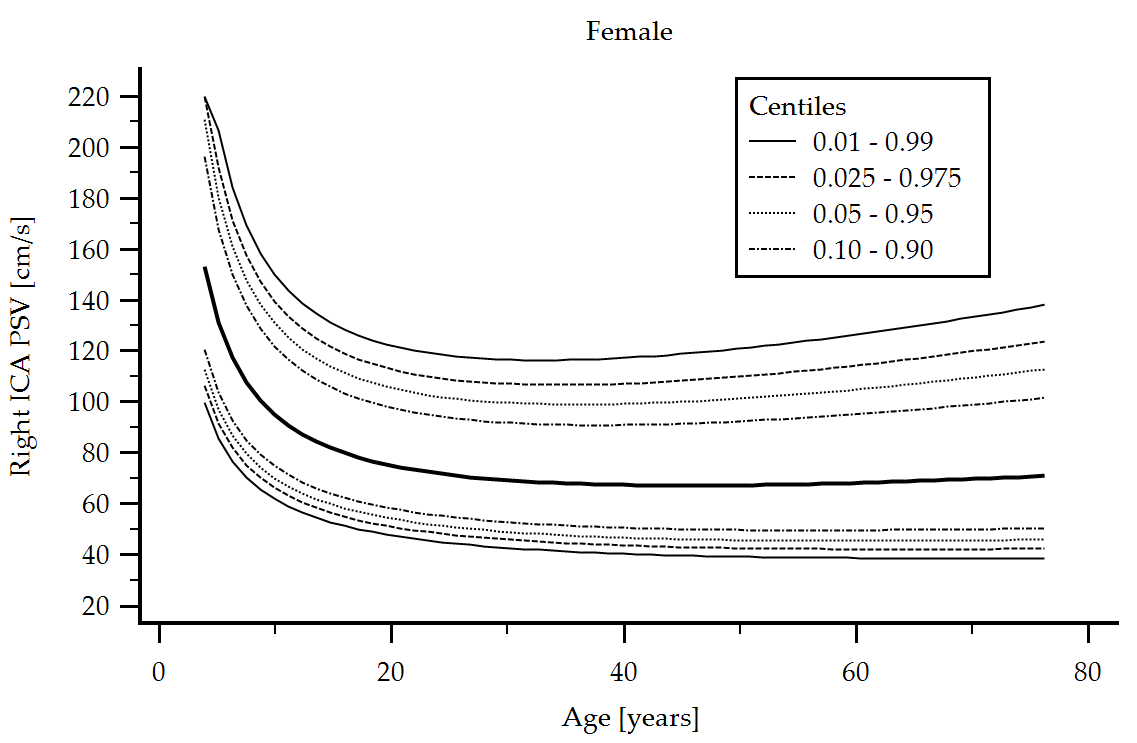

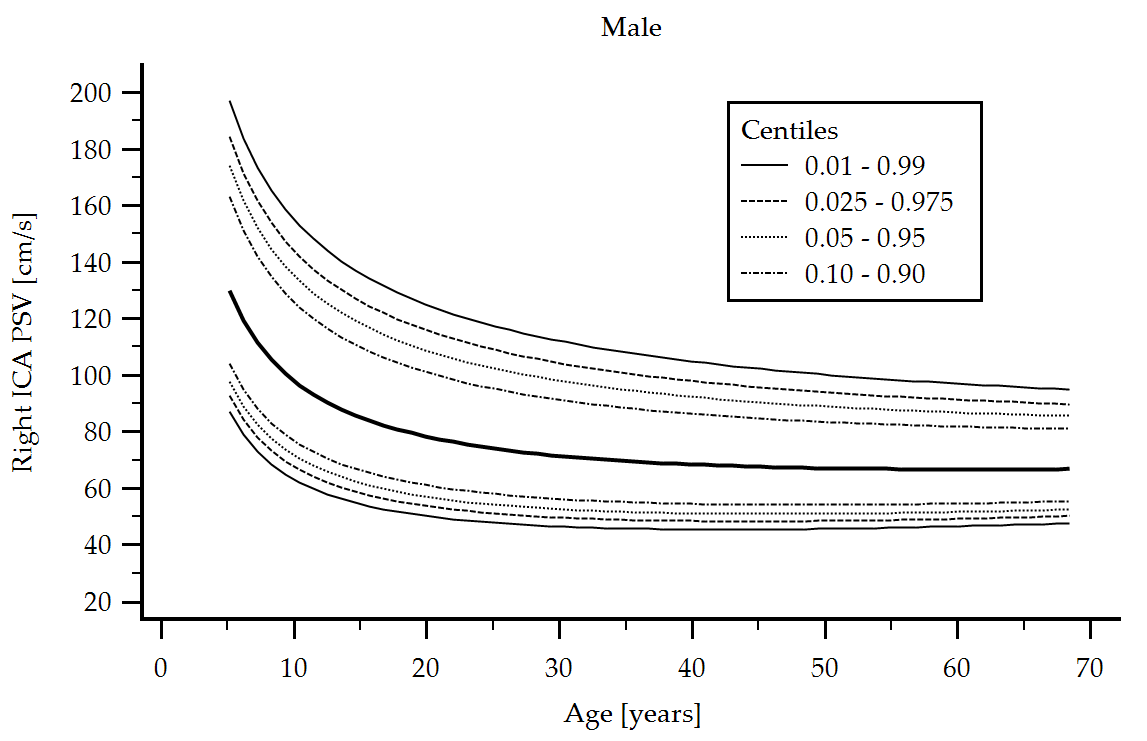


Supplementary Figure 10. Right Internal Carotid Artery (ICA) blood flow velocity percentile curves for all, females and males. PSV: peak systolic velocity.


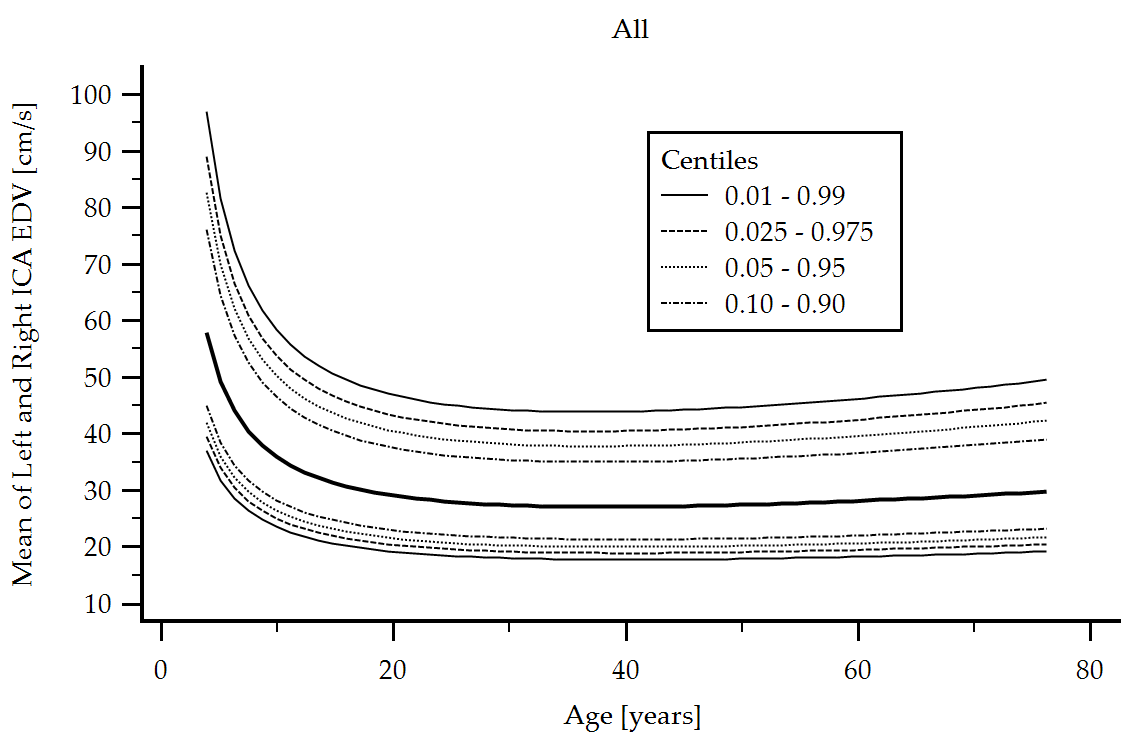


Supplementary Figure 11. Internal Carotid Artery (ICA) blood flow velocity percentile curves. EDV: end-diastolic velocity.


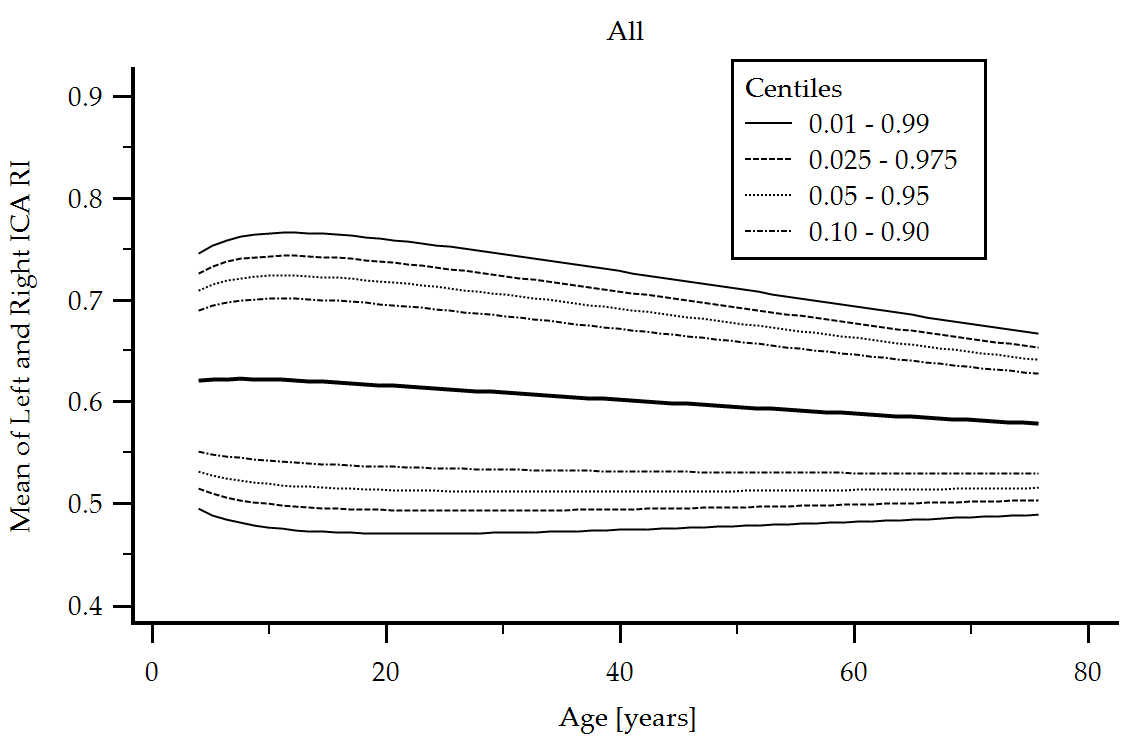

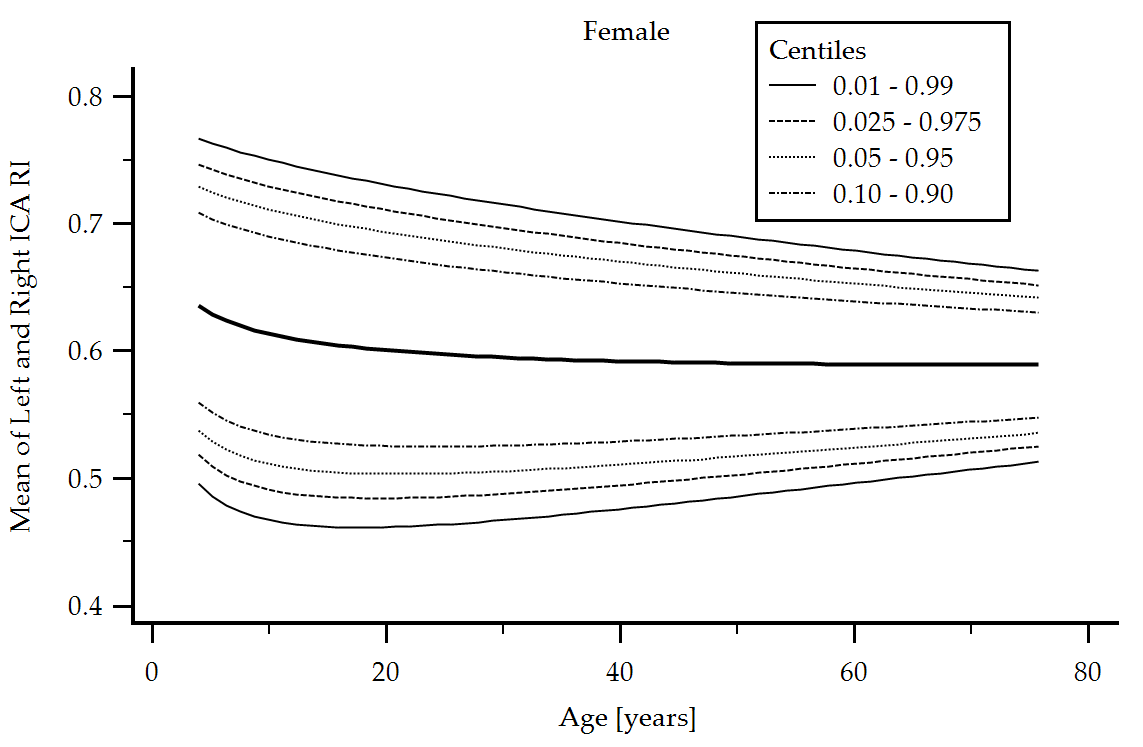

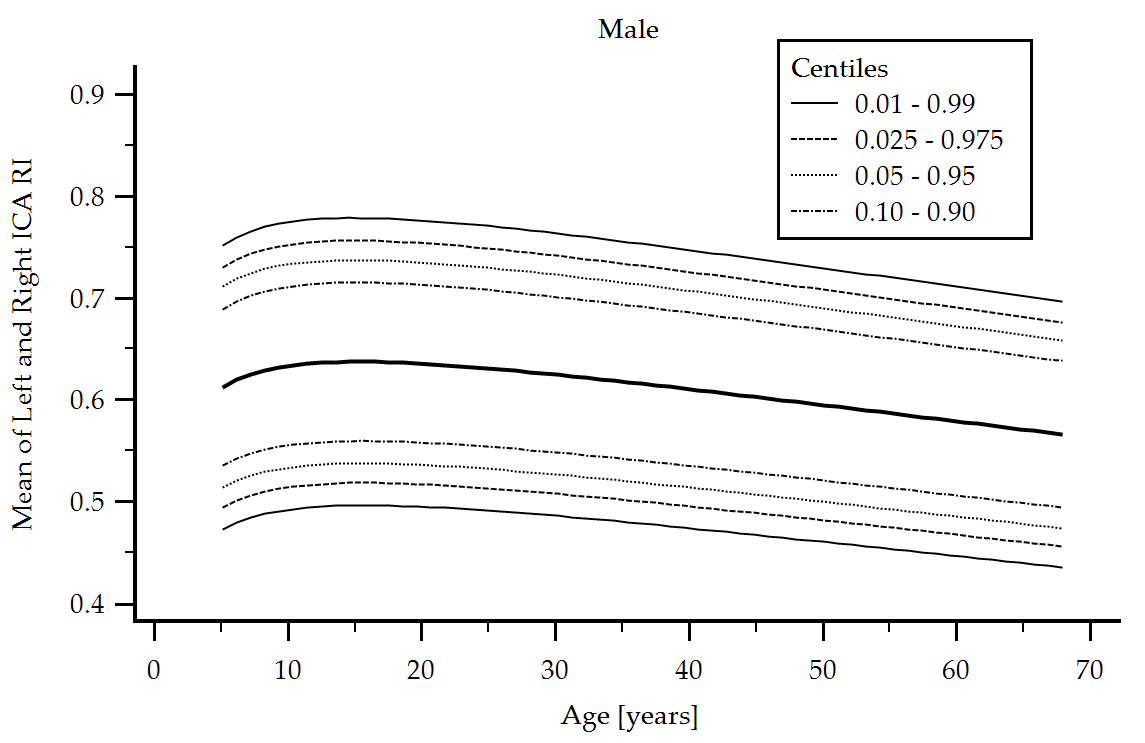


Supplementary Figure 12. Internal Carotid Artery (ICA) blood flow velocity percentile curves for all, females and males. RI: resistive index.


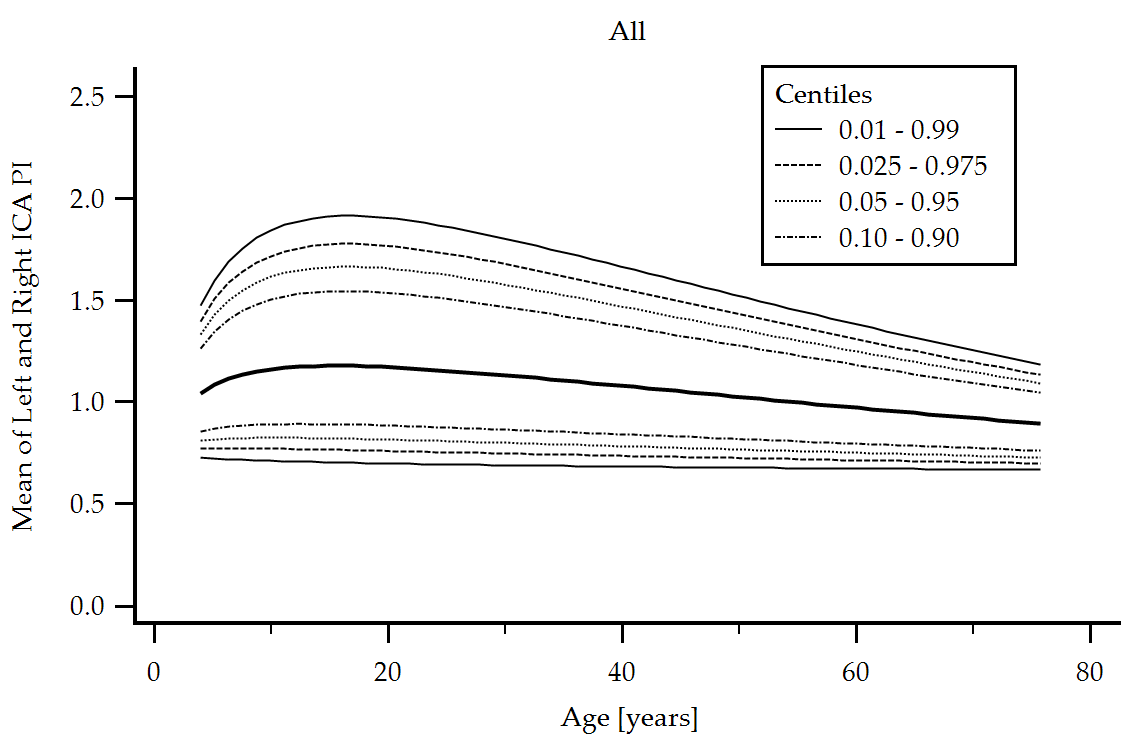

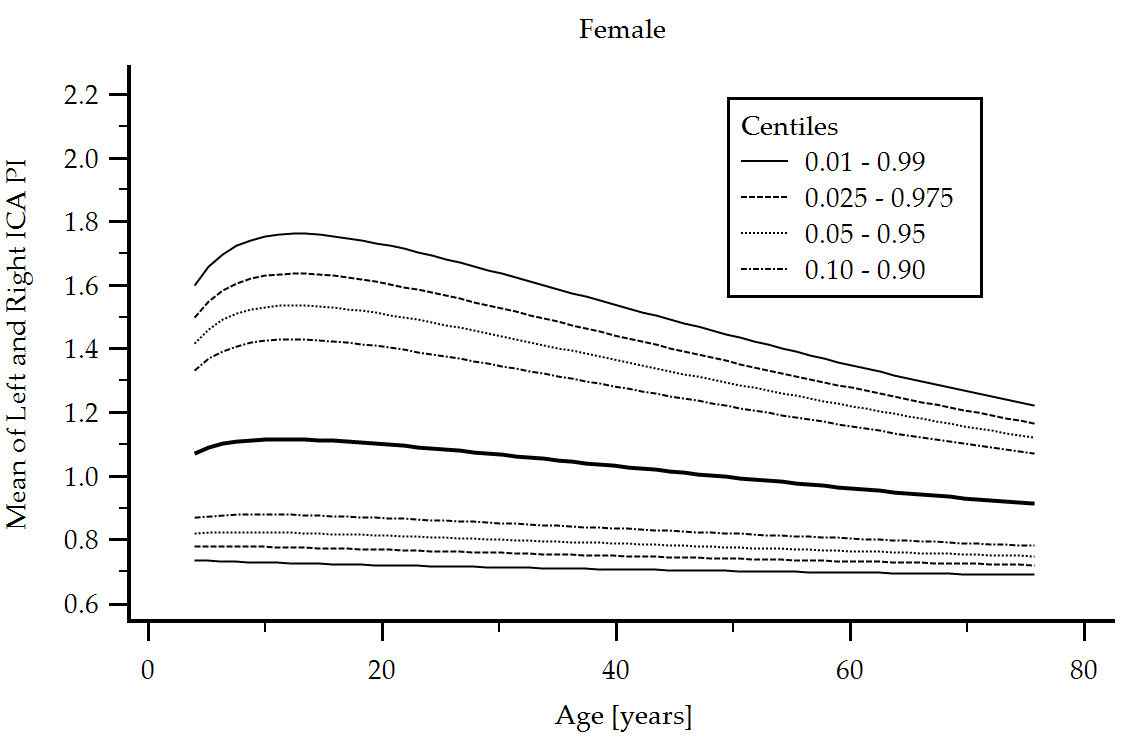

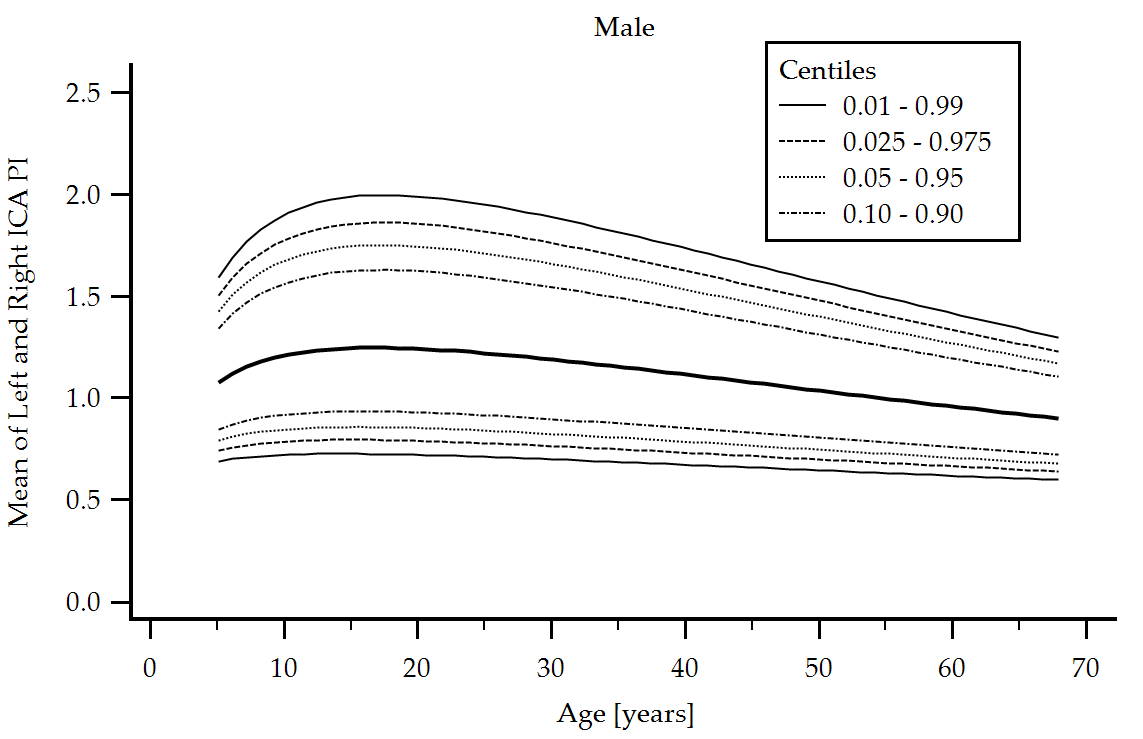


Supplementary Figure 13. Internal Carotid Artery (ICA) blood flow velocity percentile curves for all, females and males. PI: pulsatile index.


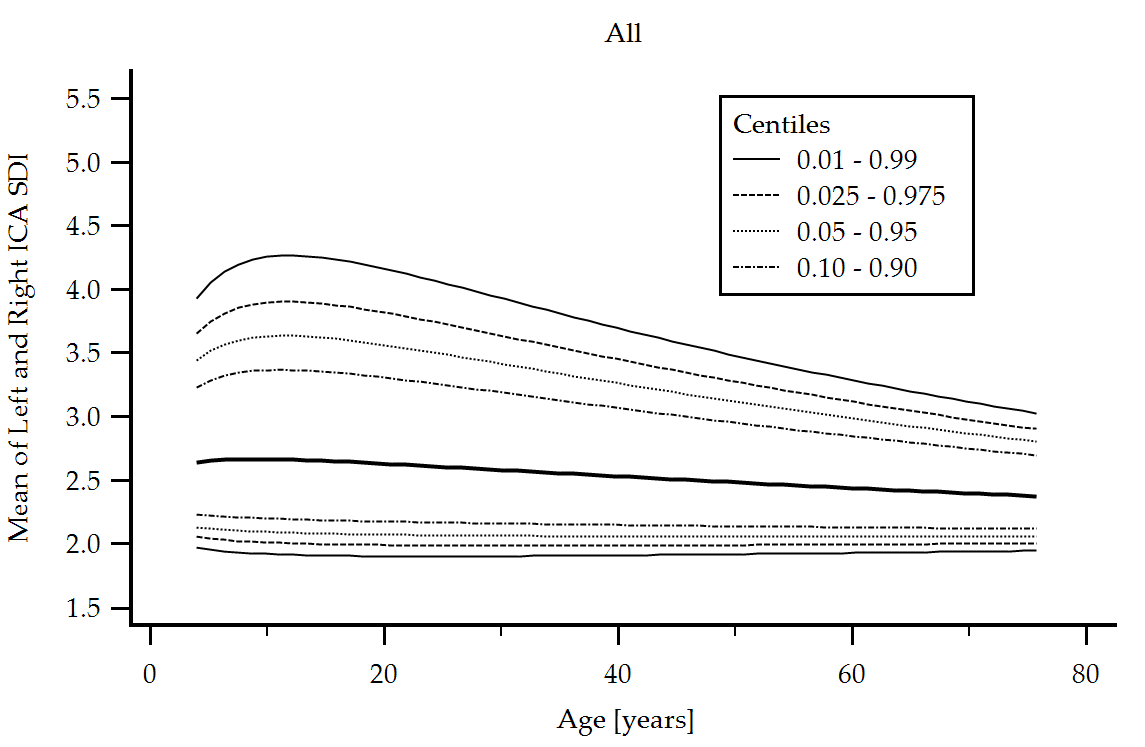

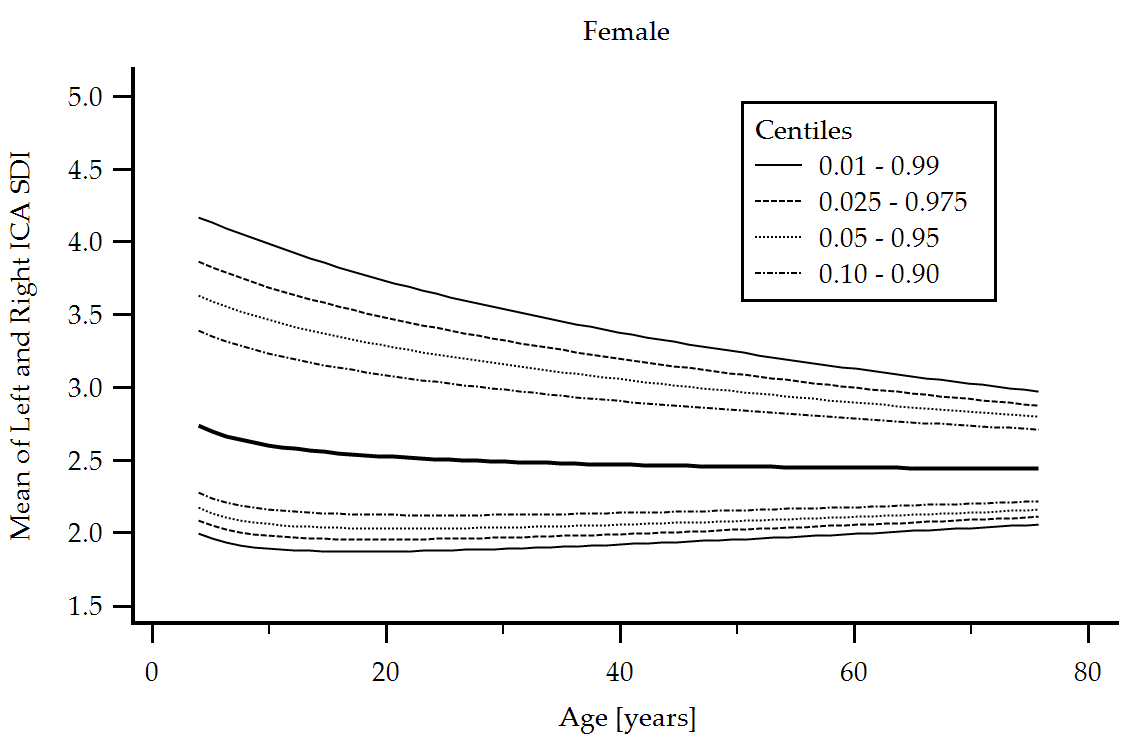

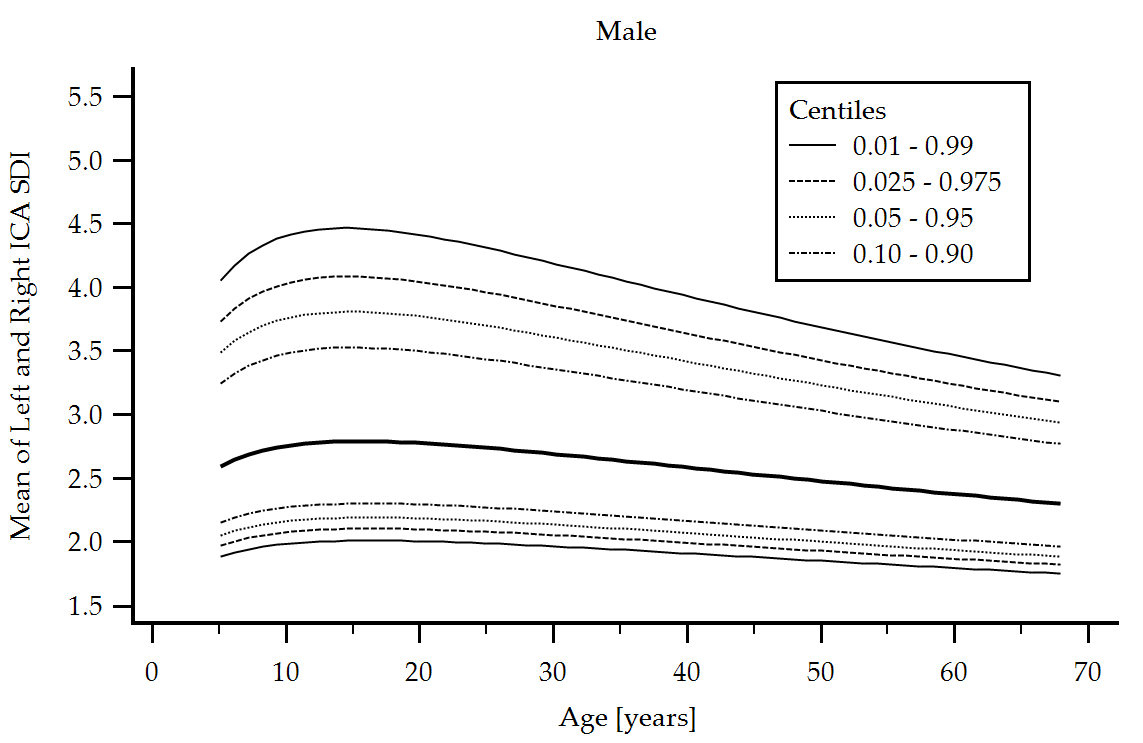


Supplementary Figure 14. Internal Carotid Artery (ICA) blood flow velocity percentile curves for all, females and males. SDI: systo-diastolic index.

**Supplementary Figures: External Carotid Artery (ECA)**

**
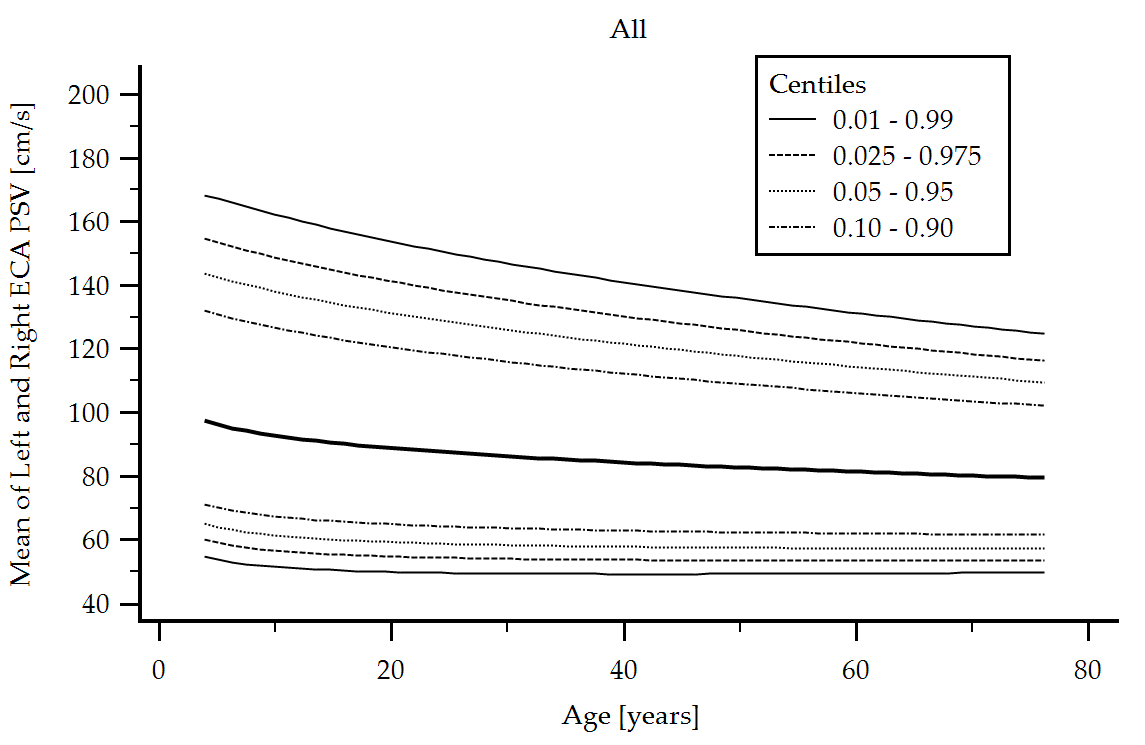

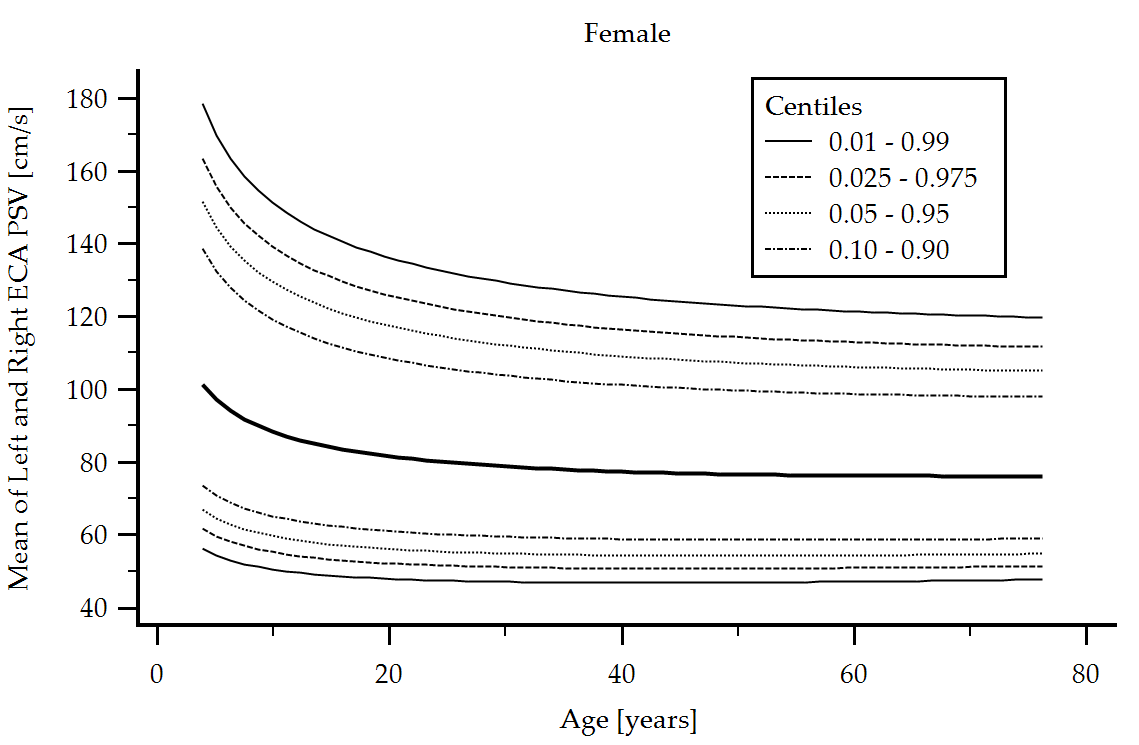

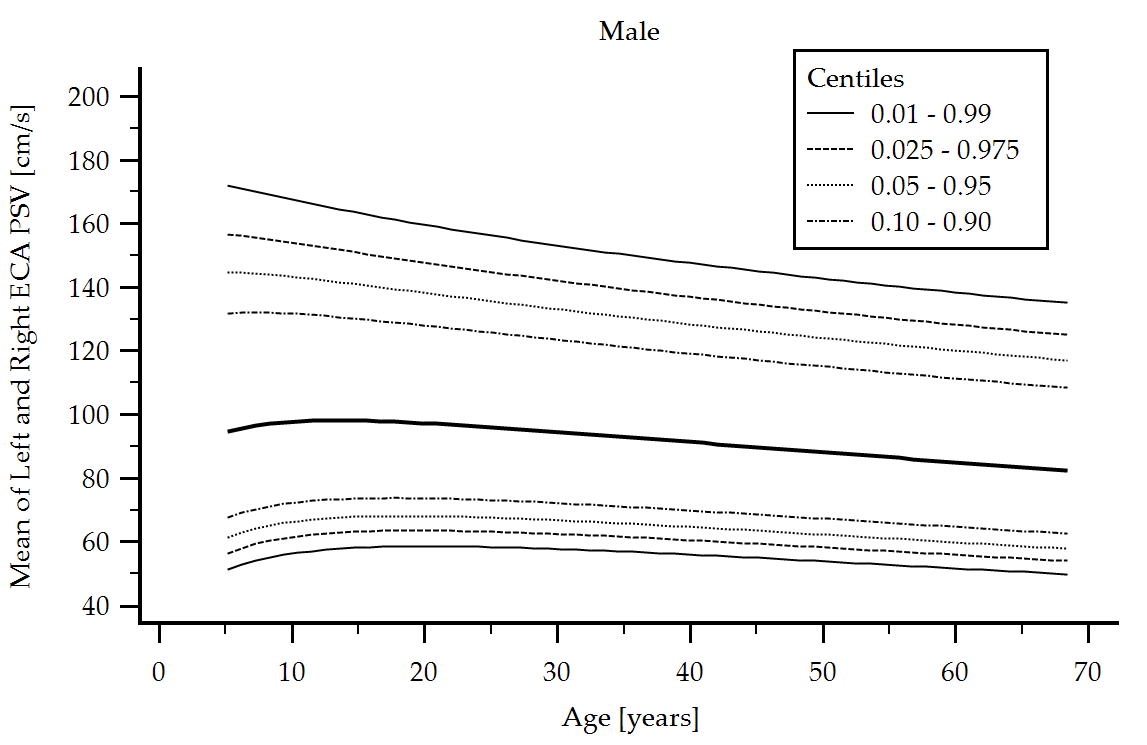
**

Supplementary Figure 15. External Carotid Artery (ECA) blood flow velocity percentile curves for all, females and males. PSV: peak systolic velocity.


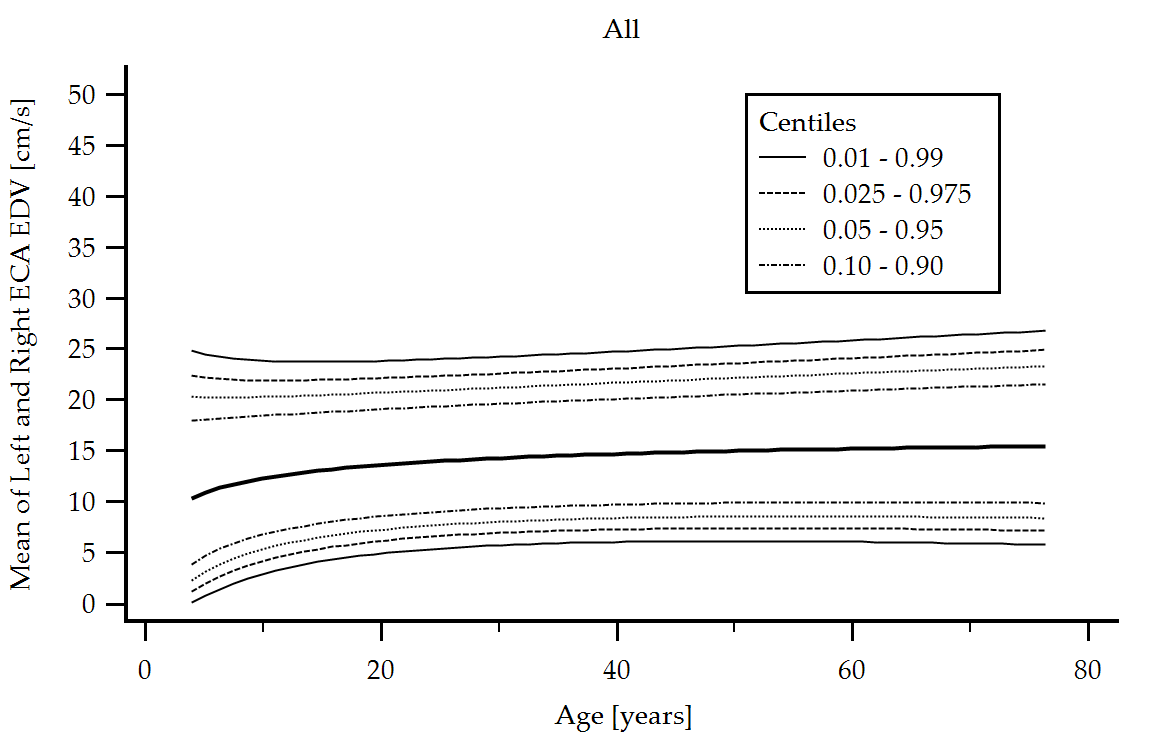

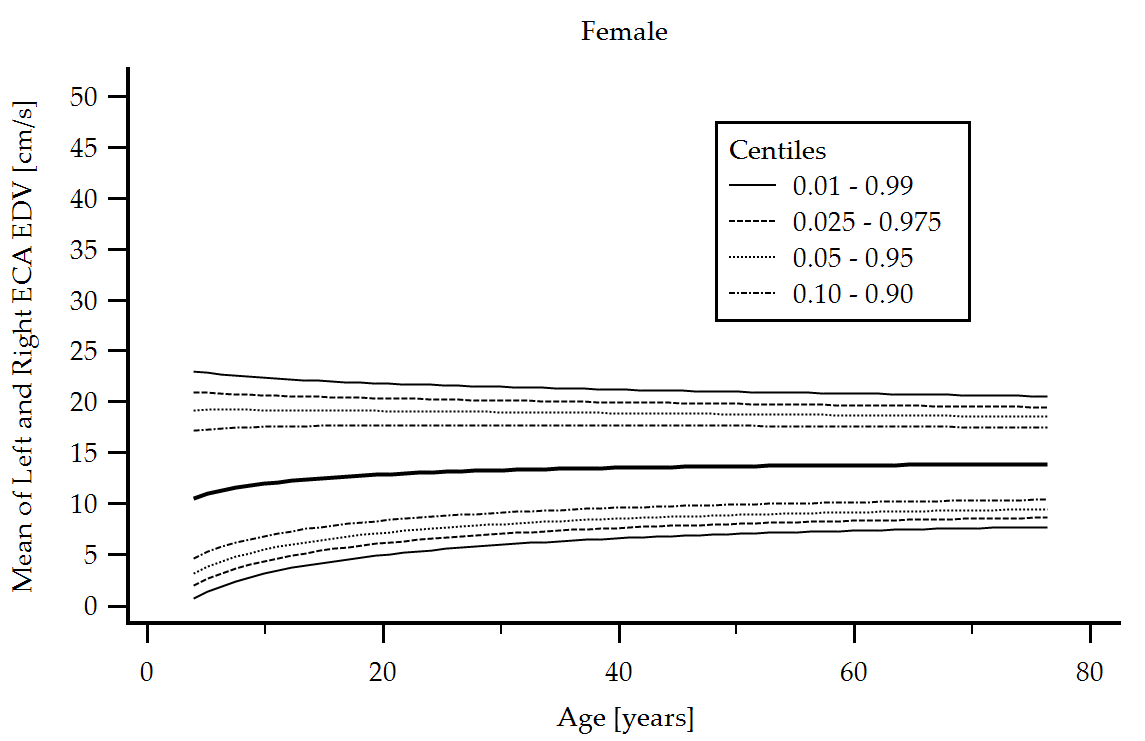

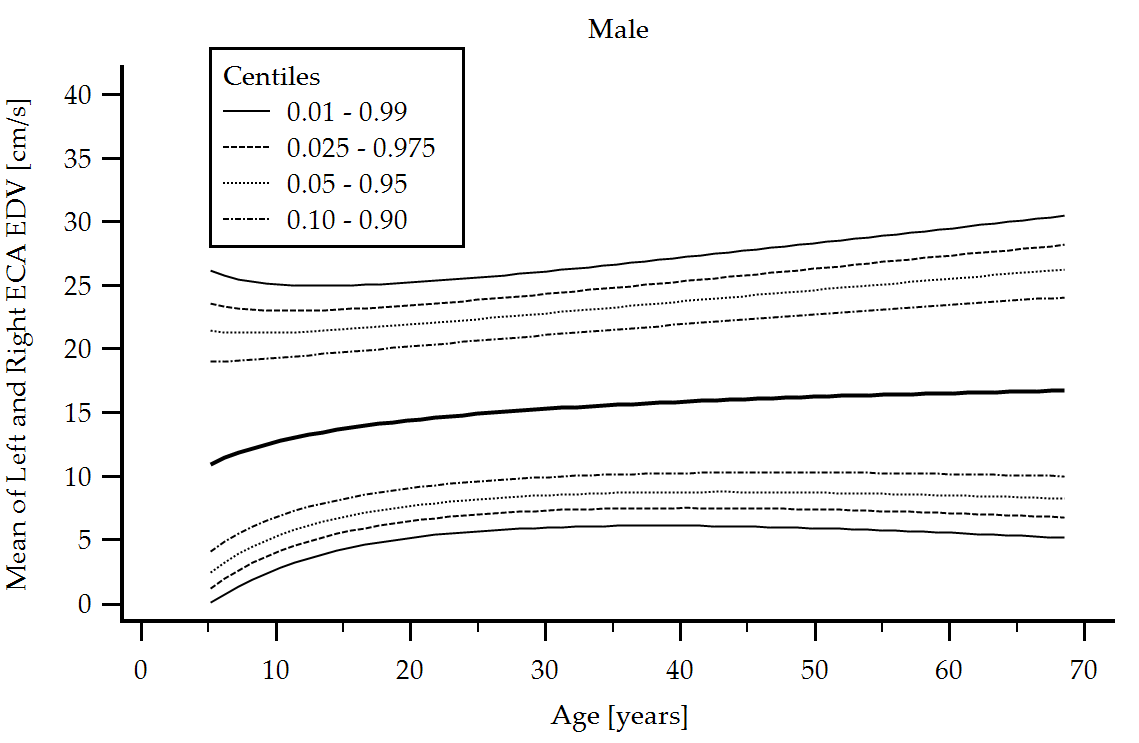


Supplementary Figure 16. External Carotid Artery (ECA) blood flow velocity percentile curves for all, females and males. EDV: end-diastolic velocity.


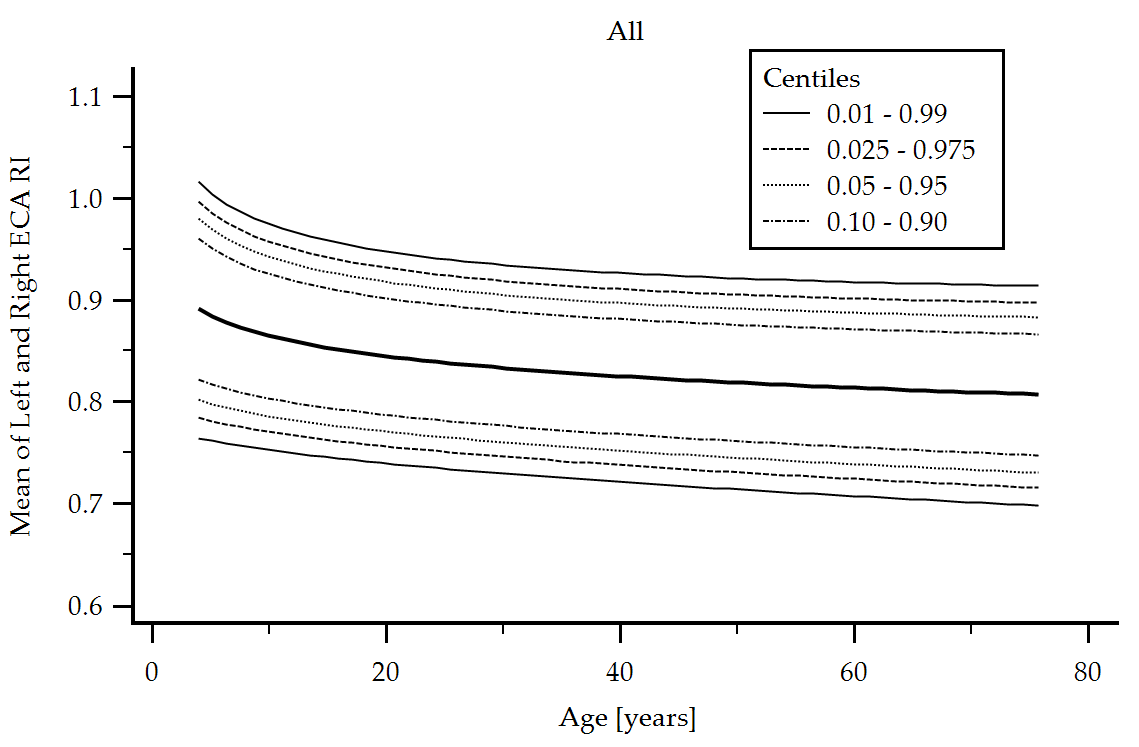


Supplementary Figure 17. External Carotid Artery (ECA) blood flow velocity percentile curves. RI: resistive index.


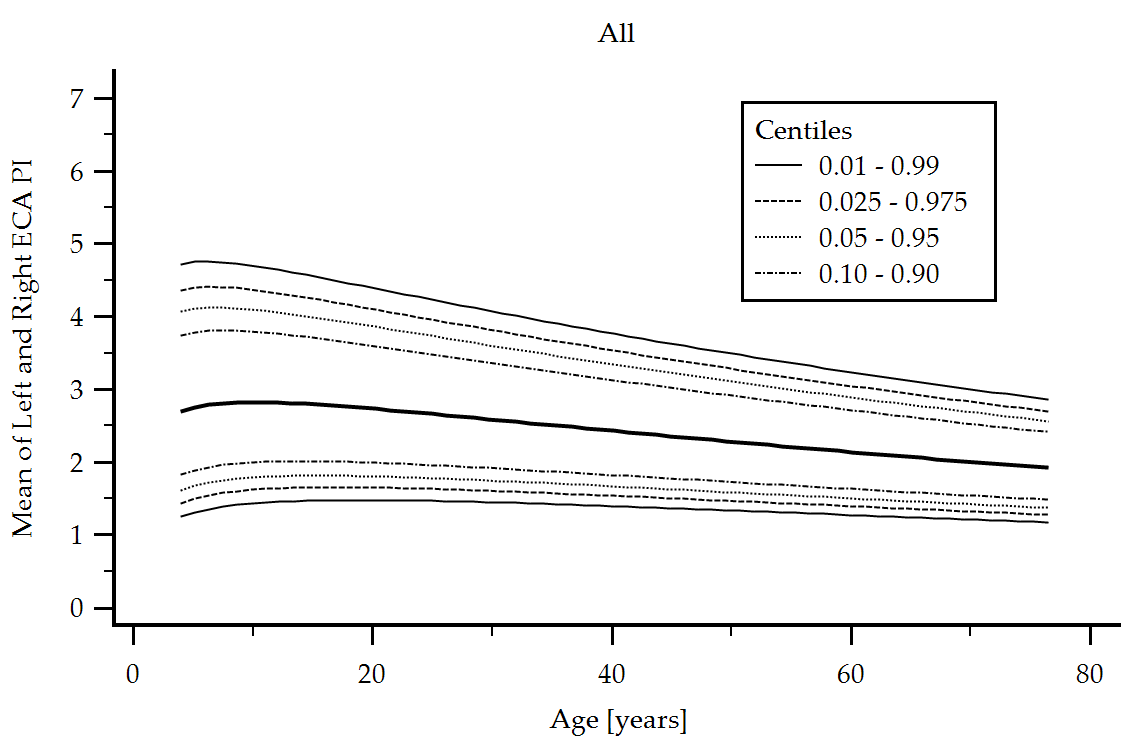

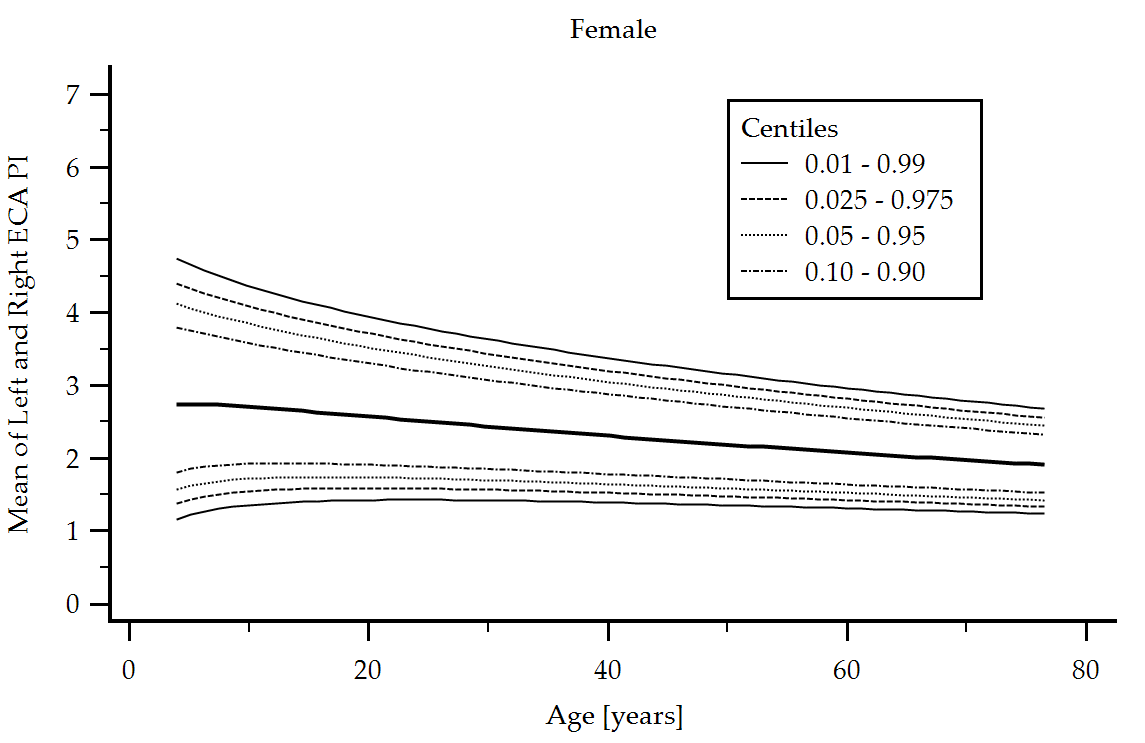

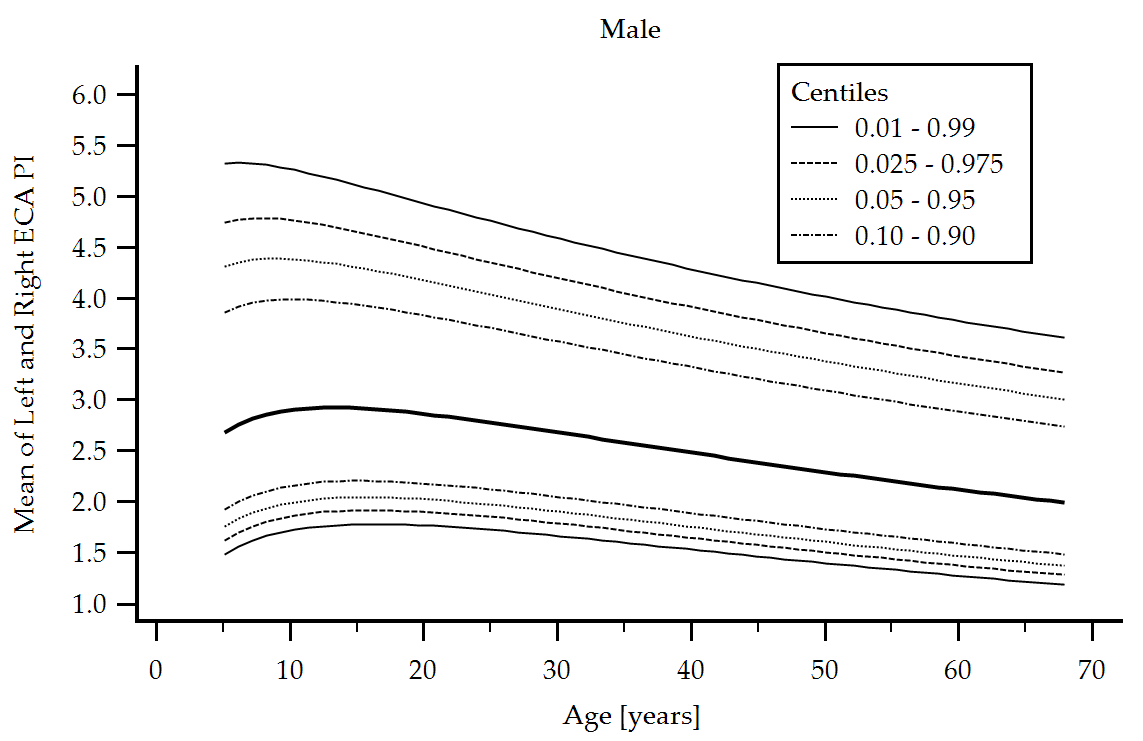


Supplementary Figure 18. External Carotid Artery (ECA) blood flow velocity percentile curves for all, females and males. PI: pulsatile index.


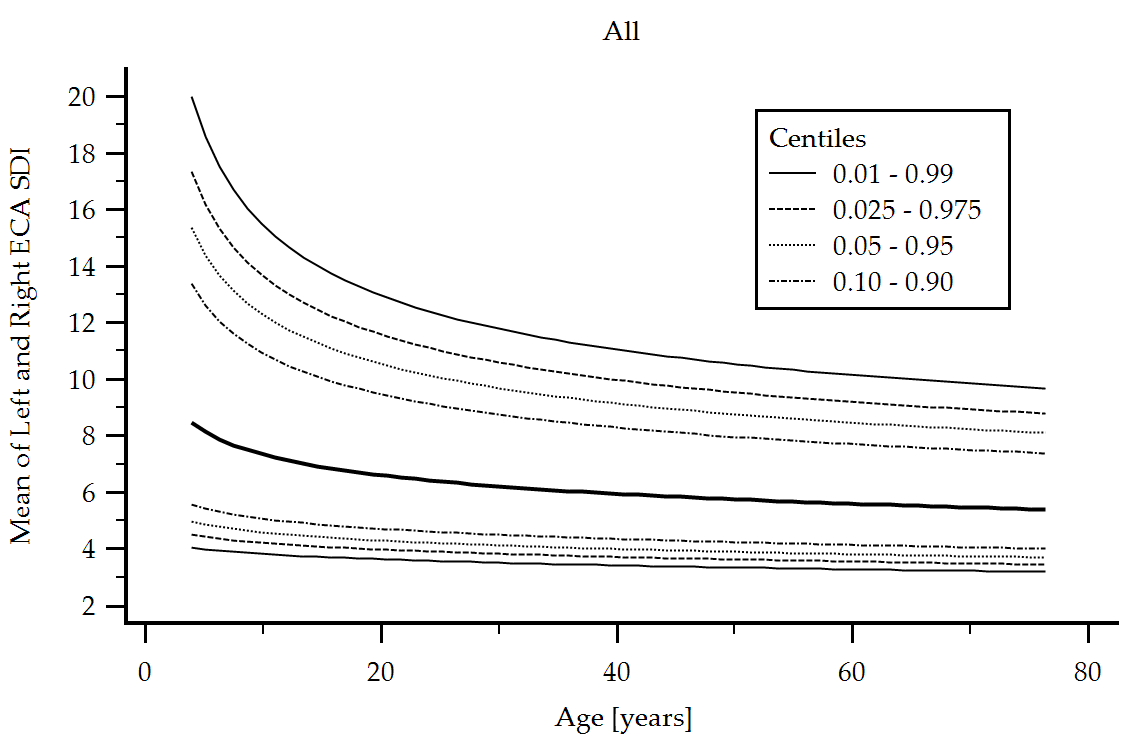


Supplementary Figure 19. External Carotid Artery (ECA) blood flow velocity percentile curves. SDI: systo-diastolic index.

**Supplementary Figures: Peak Systolic Velocity Ratio (PSVR)**


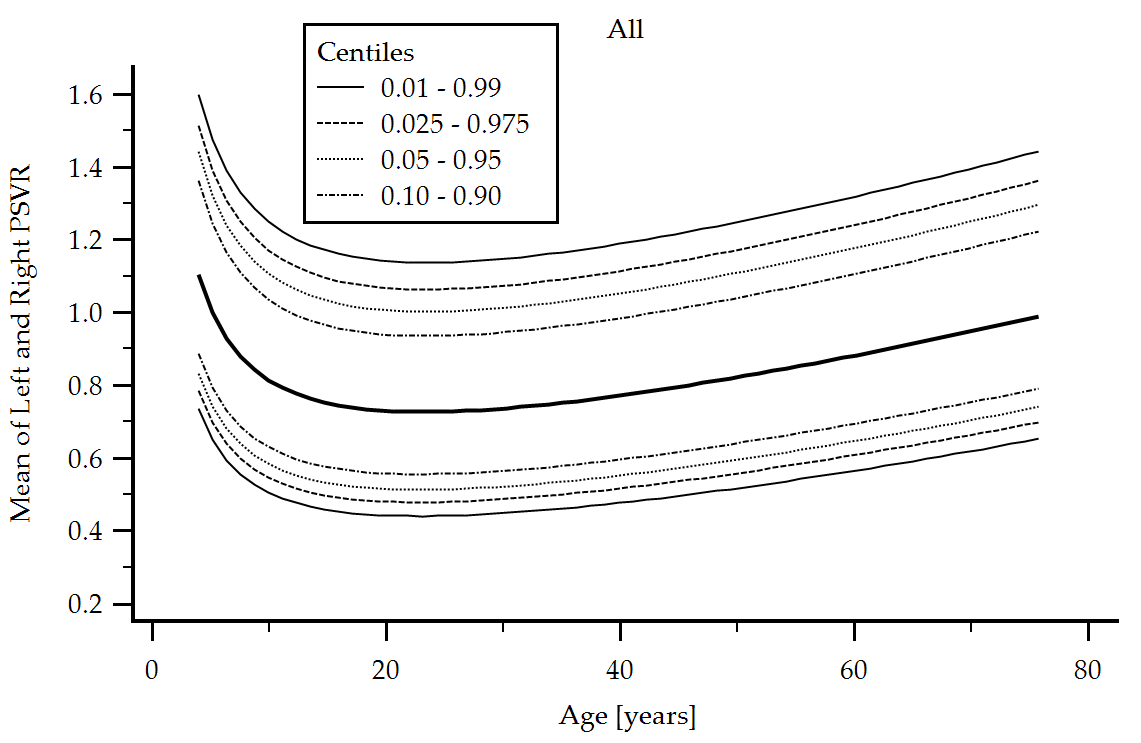

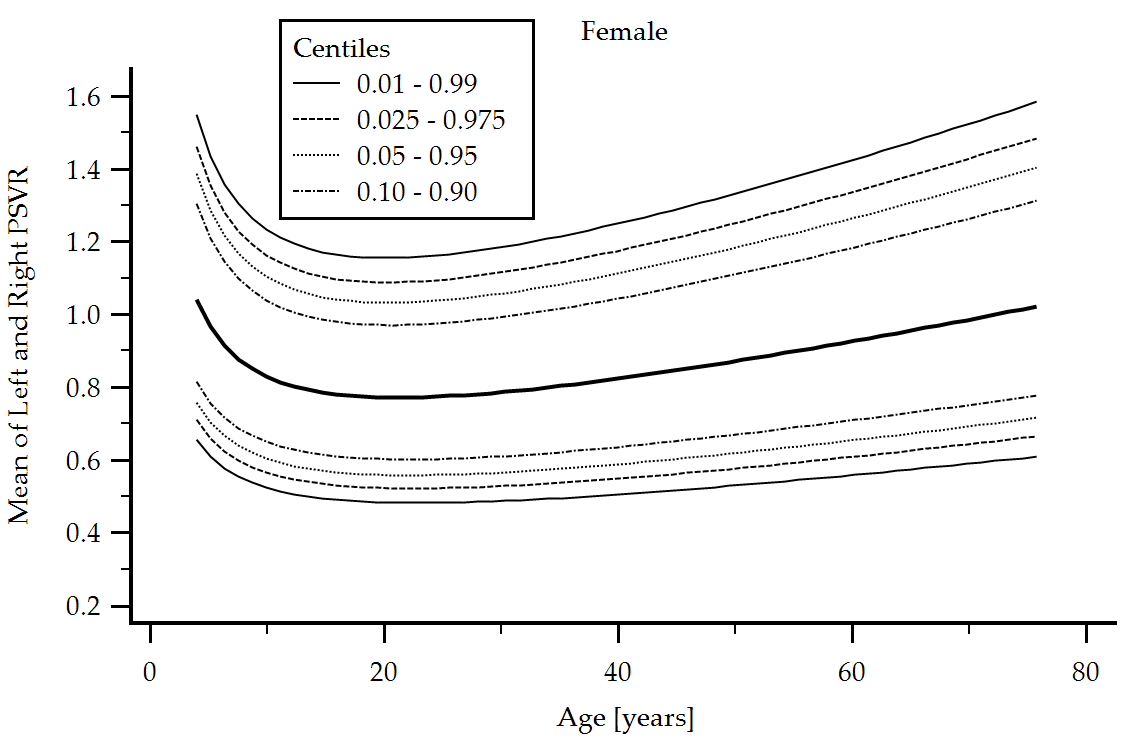

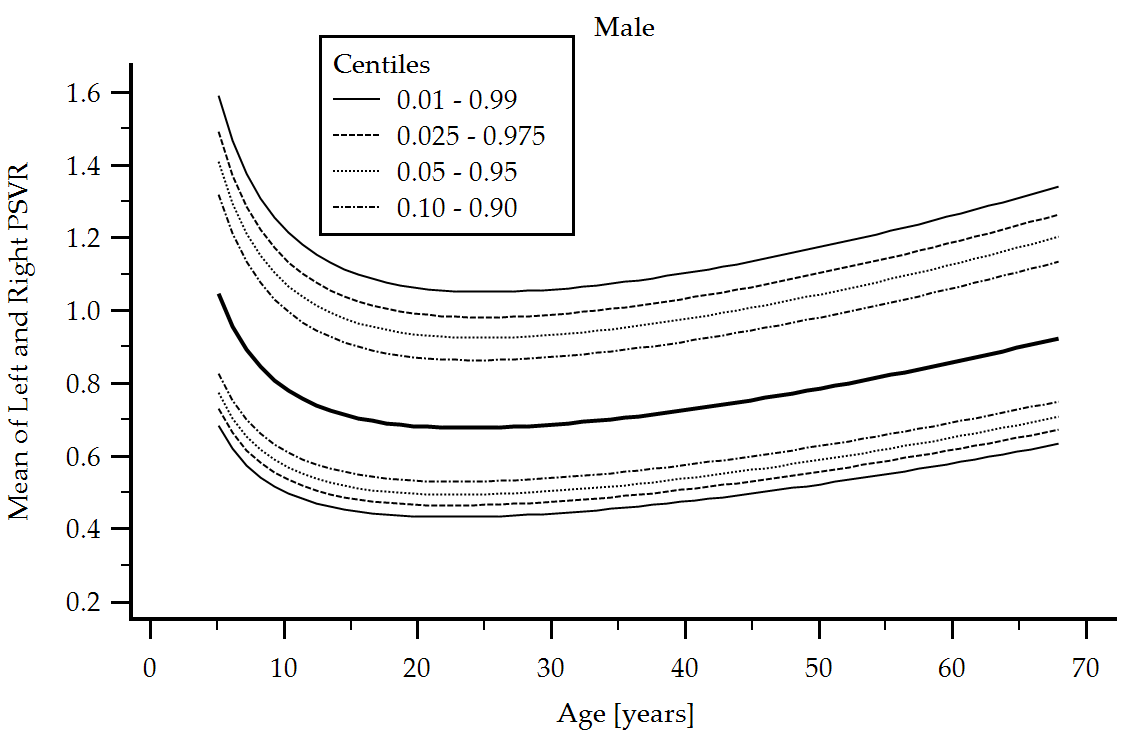


Supplementary Figure 20. Peak systolic velocity ratio (PSVR) percentile curves for all, females and males.

**Supplementary Figure: St´Mary Ratio (SMR)**


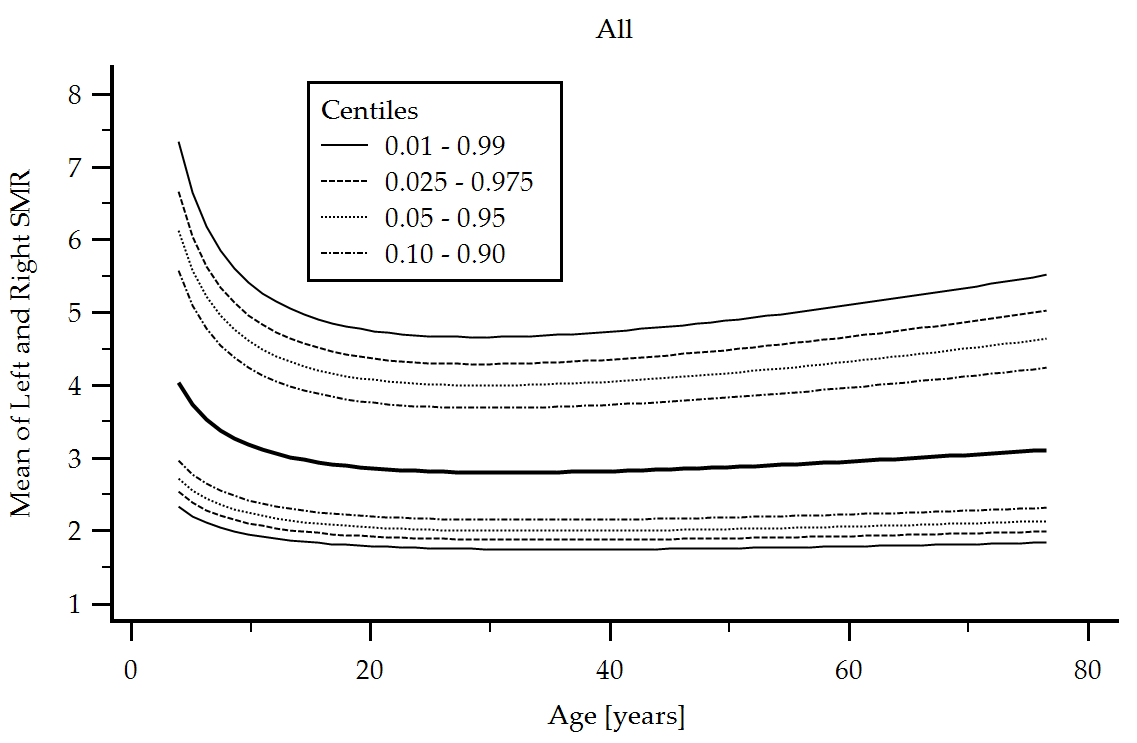


Supplementary Figure 21. St´mary Ratio (SMR) percentile curves.

**Supplementary Figures: Vertebral Artery (VA)**


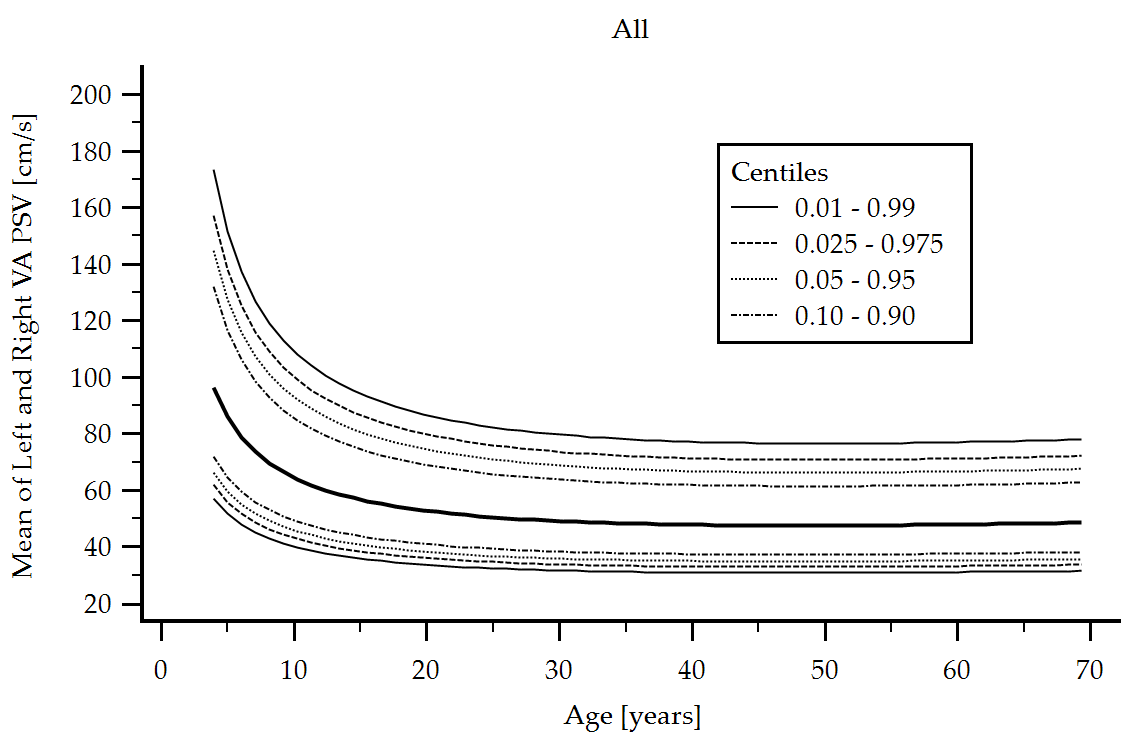

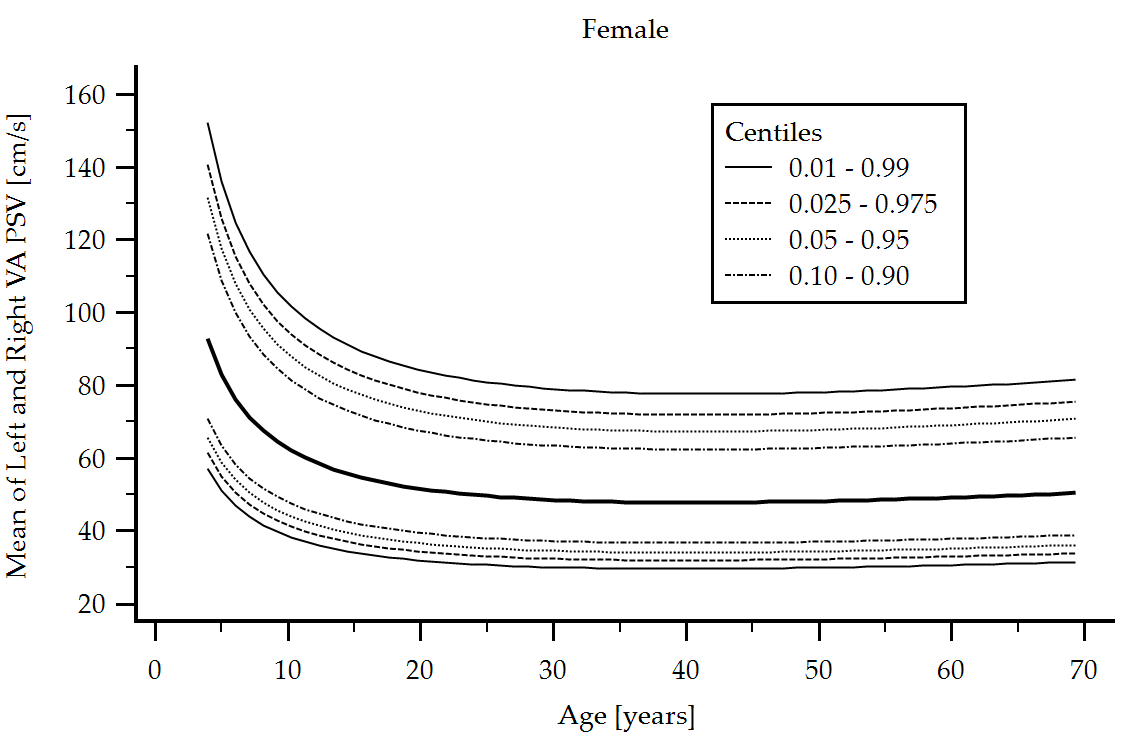

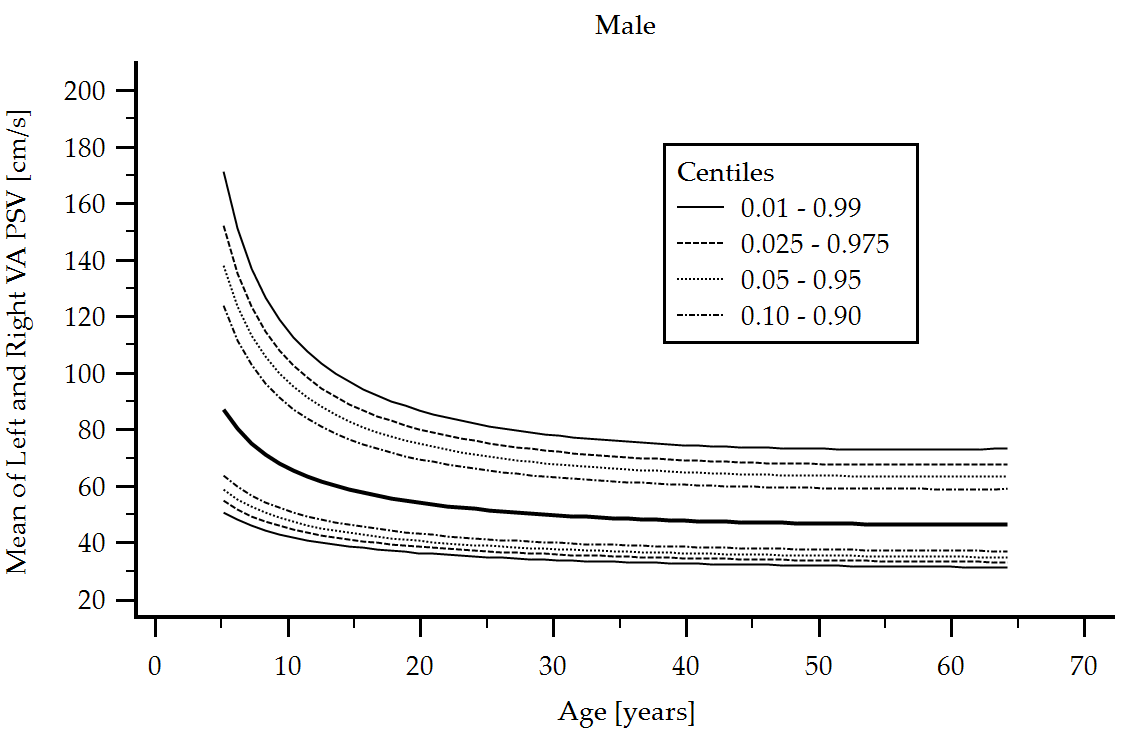


Supplementary Figure 22. Vertebral Artery (VA) blood flow velocity percentile curves for all, females and males. PSV: peak systolic velocity.


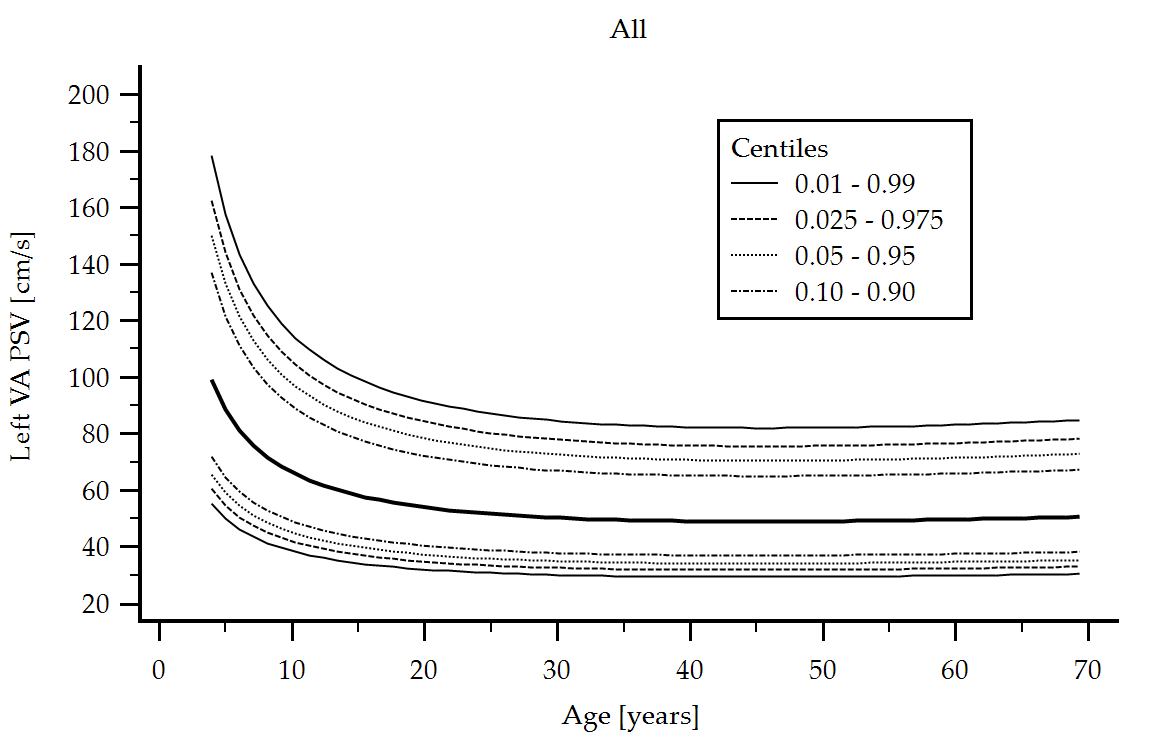

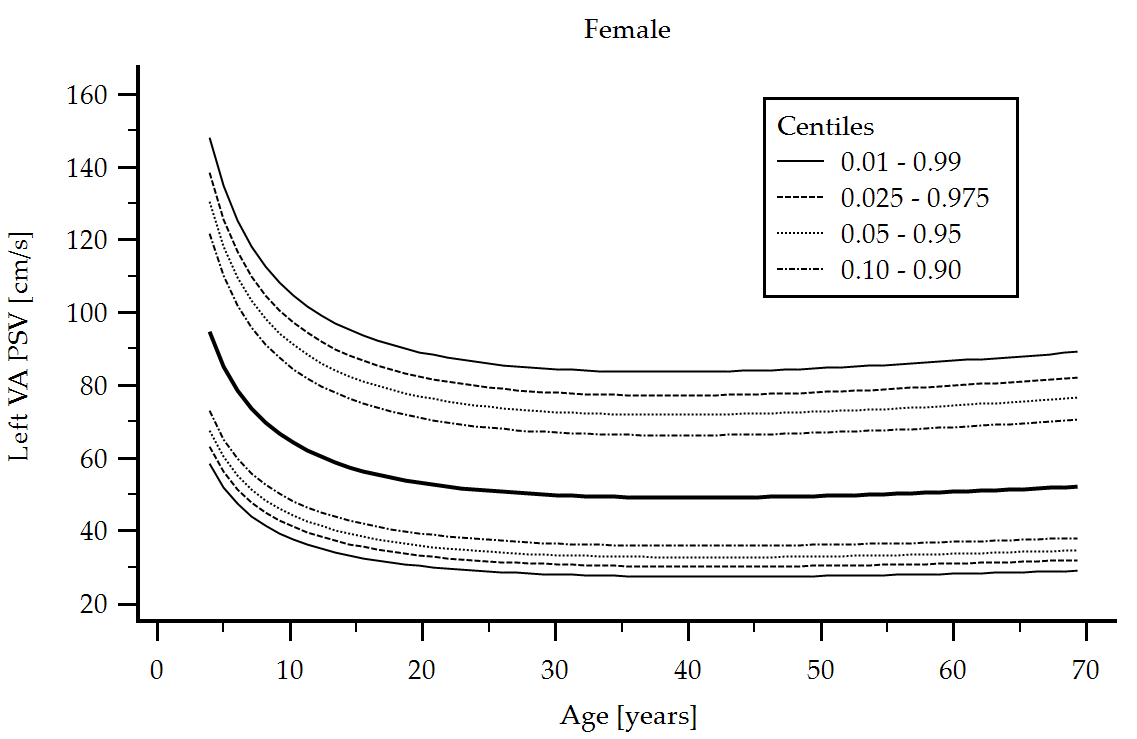

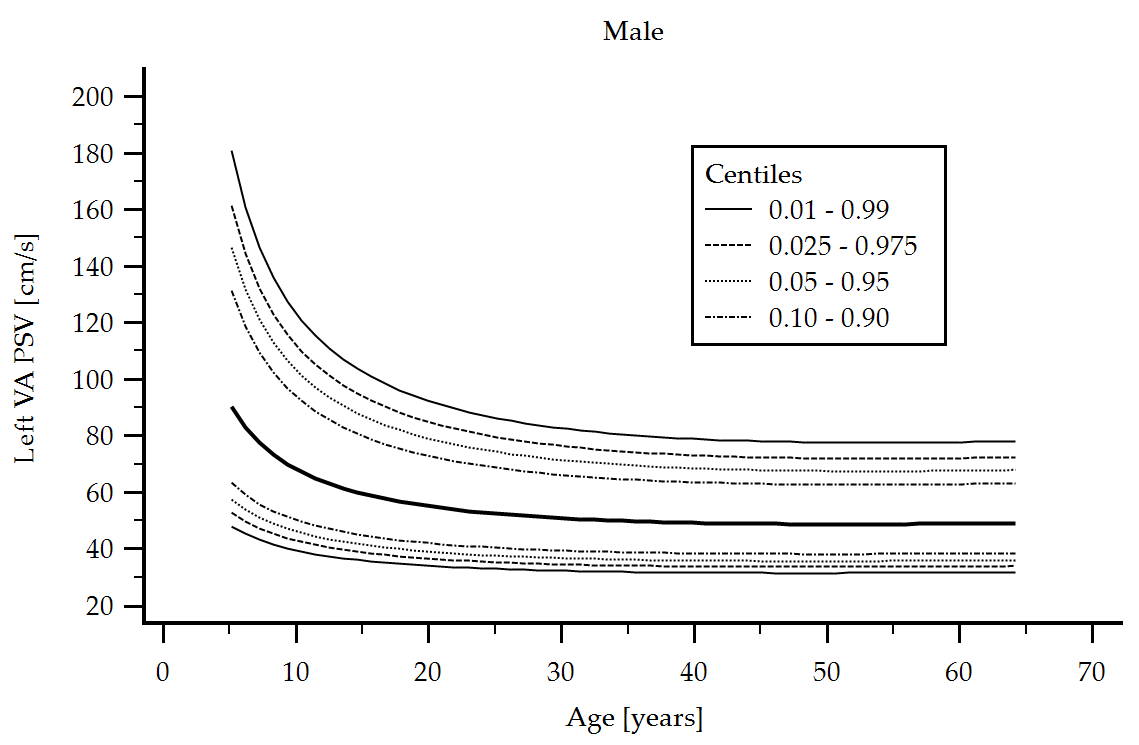


Supplementary Figure 23. Left Vertebral Artery (VA) blood flow velocity percentile curves for all, females and males. PSV: peak systolic velocity.


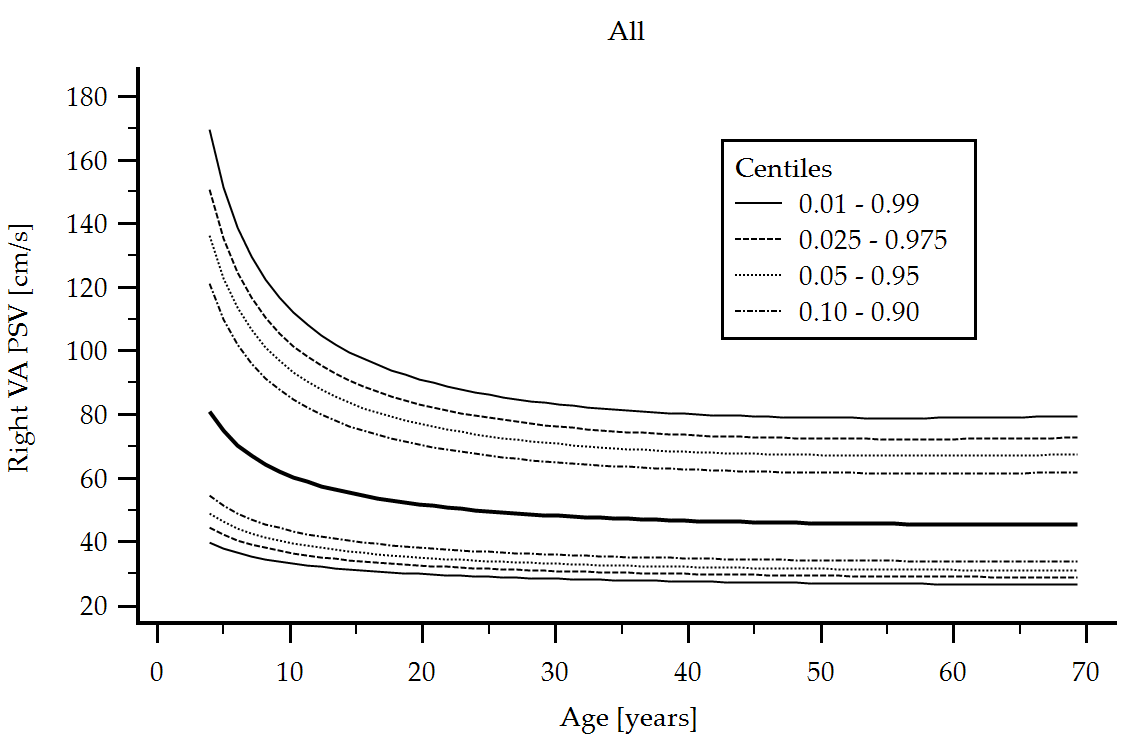

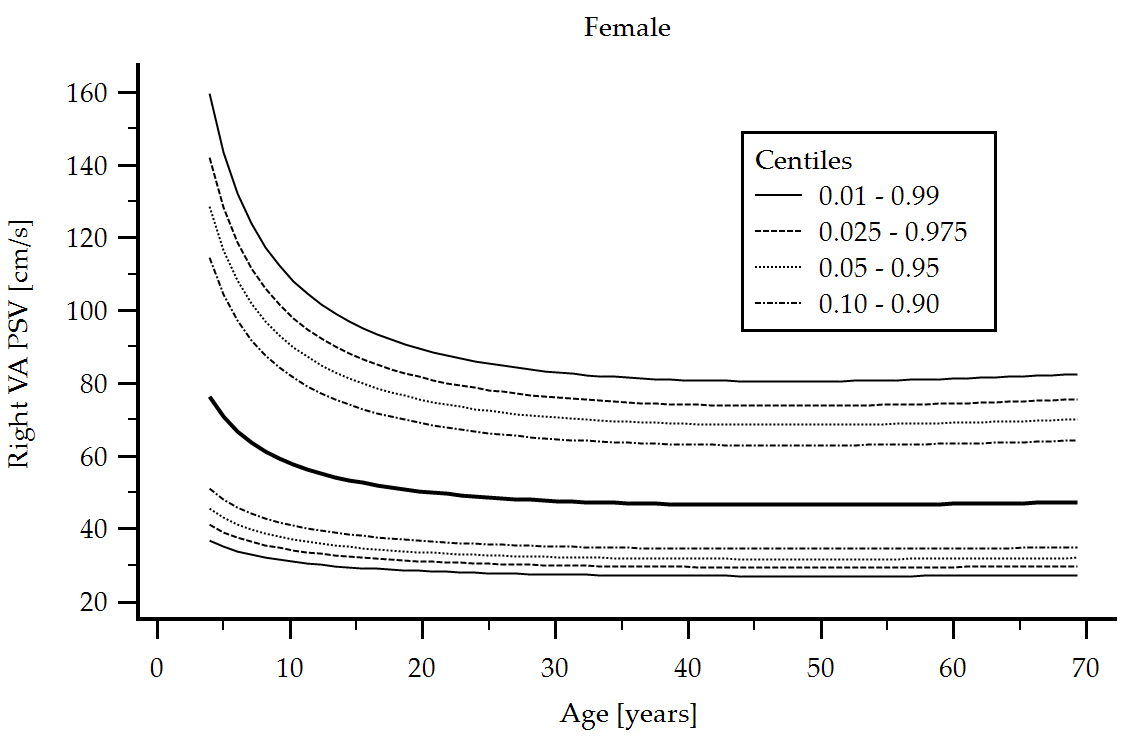

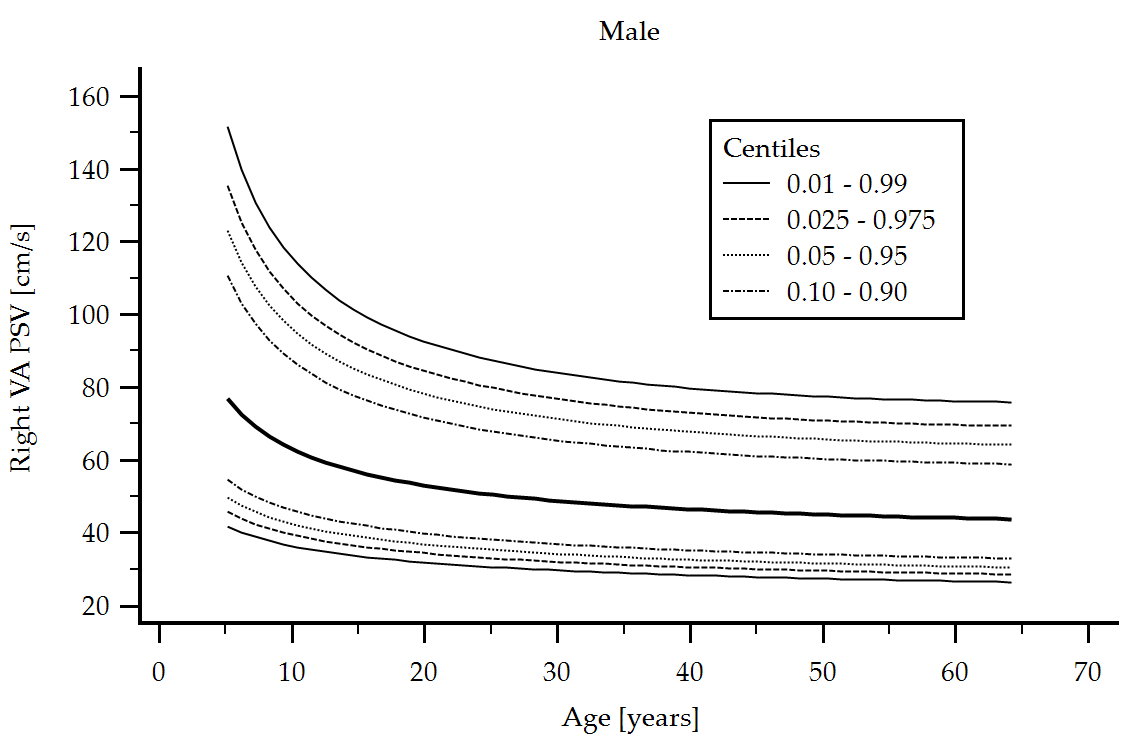


Supplementary Figure 24. Right Vertebral Artery (VA) blood flow velocity percentile curves for all, females and males. PSV: peak systolic velocity.


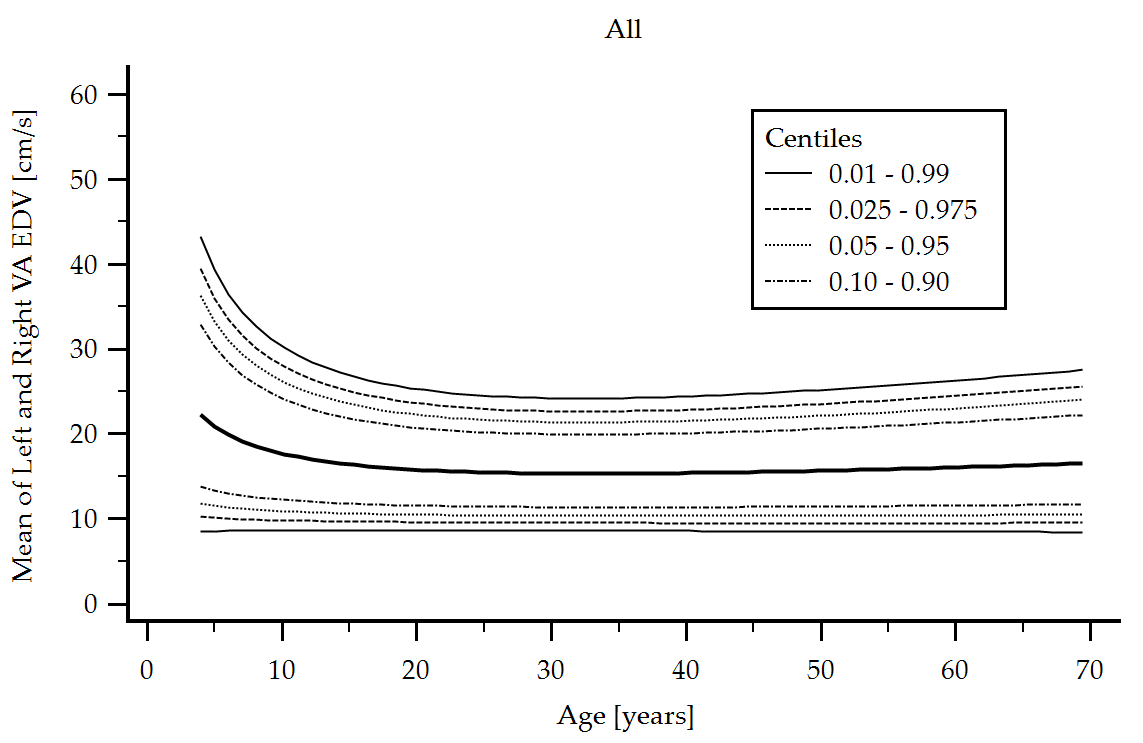

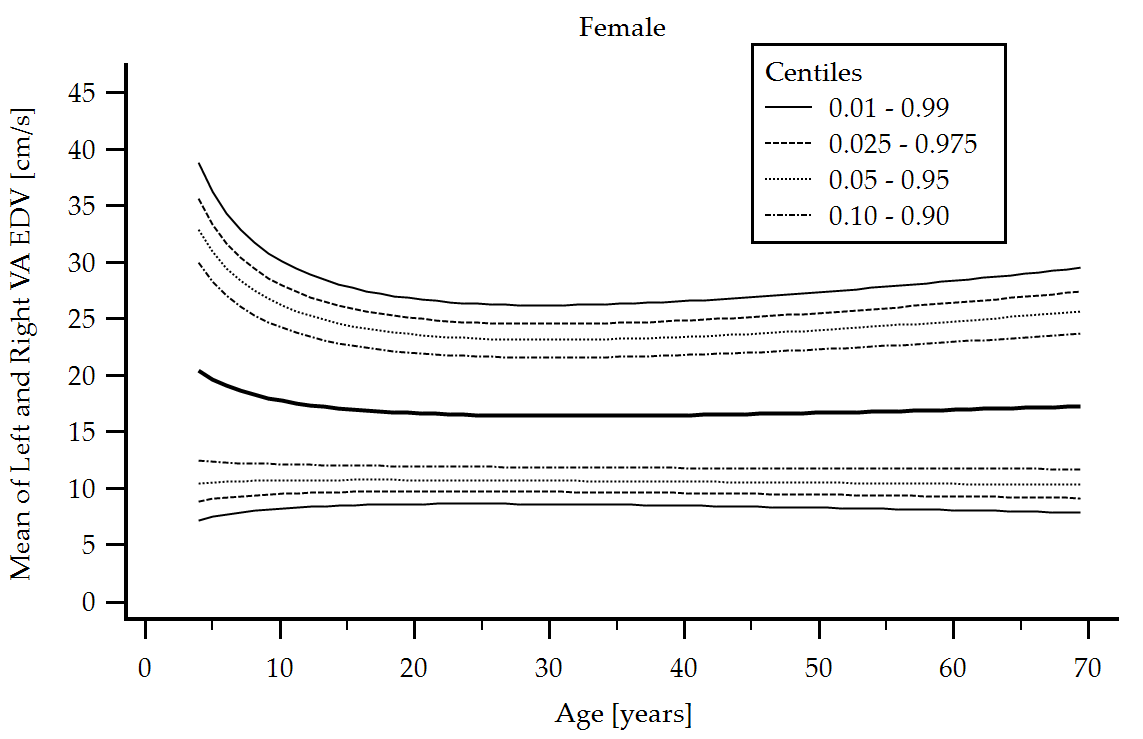

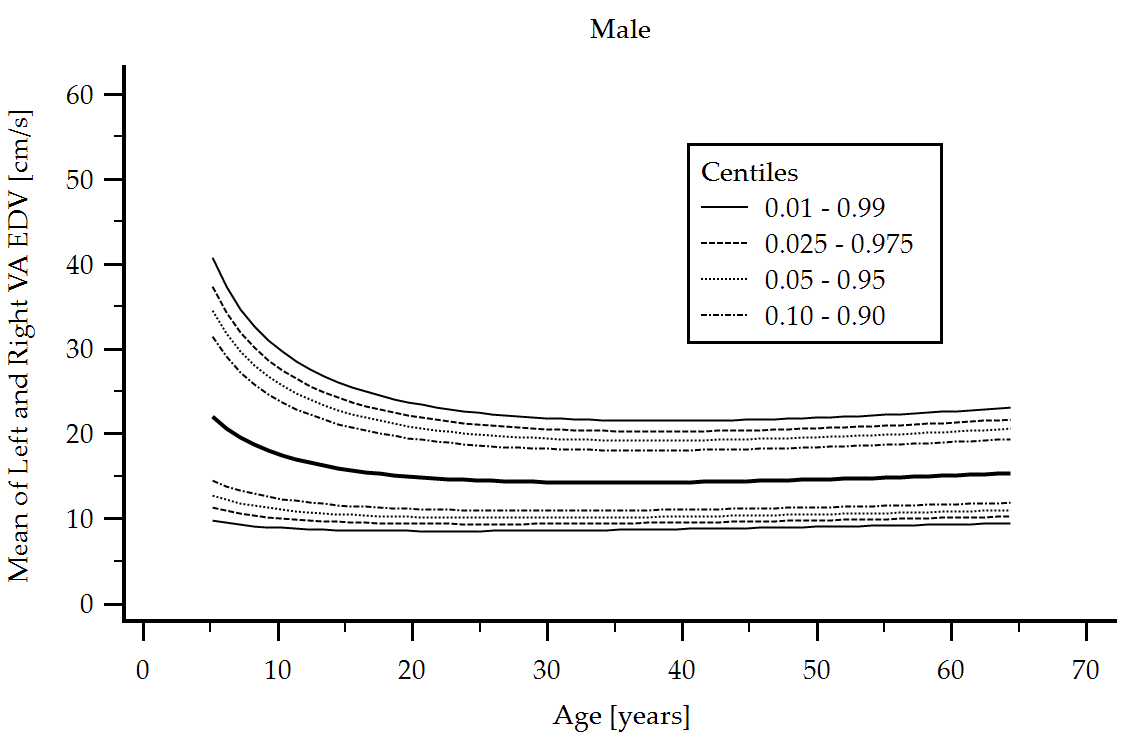


Supplementary Figure 25. Vertebral Artery (VA) blood flow velocity percentile curves for all, females and males. EDV: end-diastolic velocity.


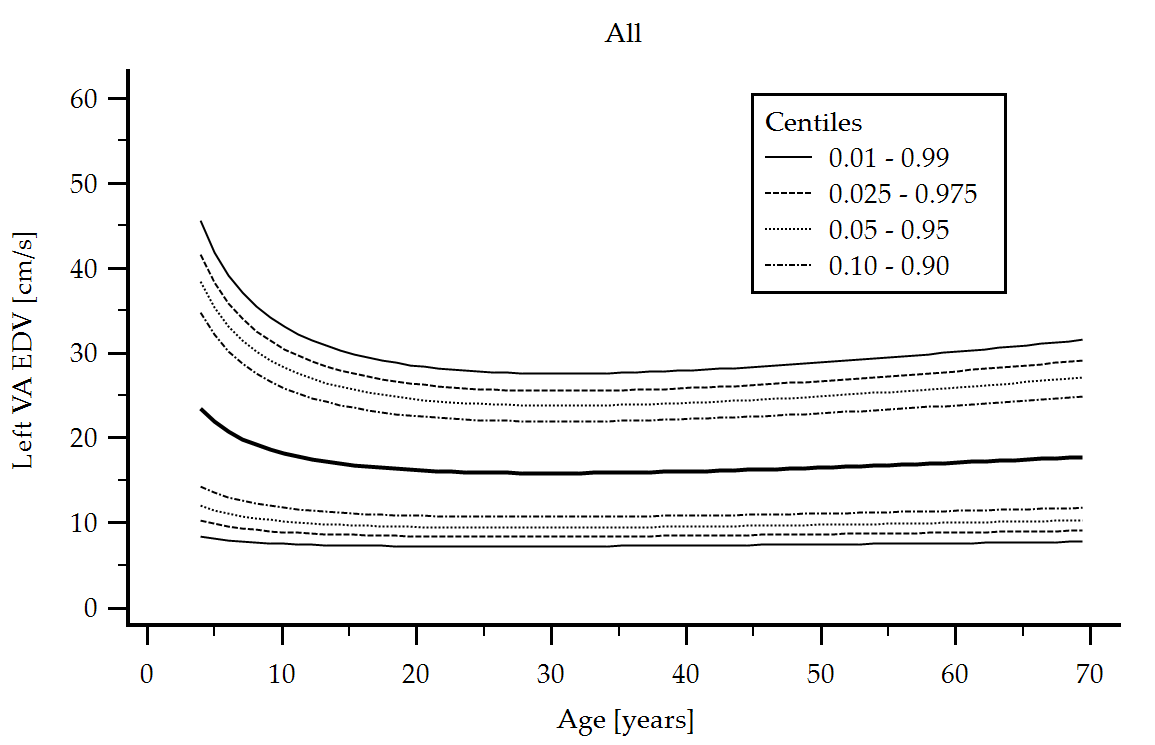

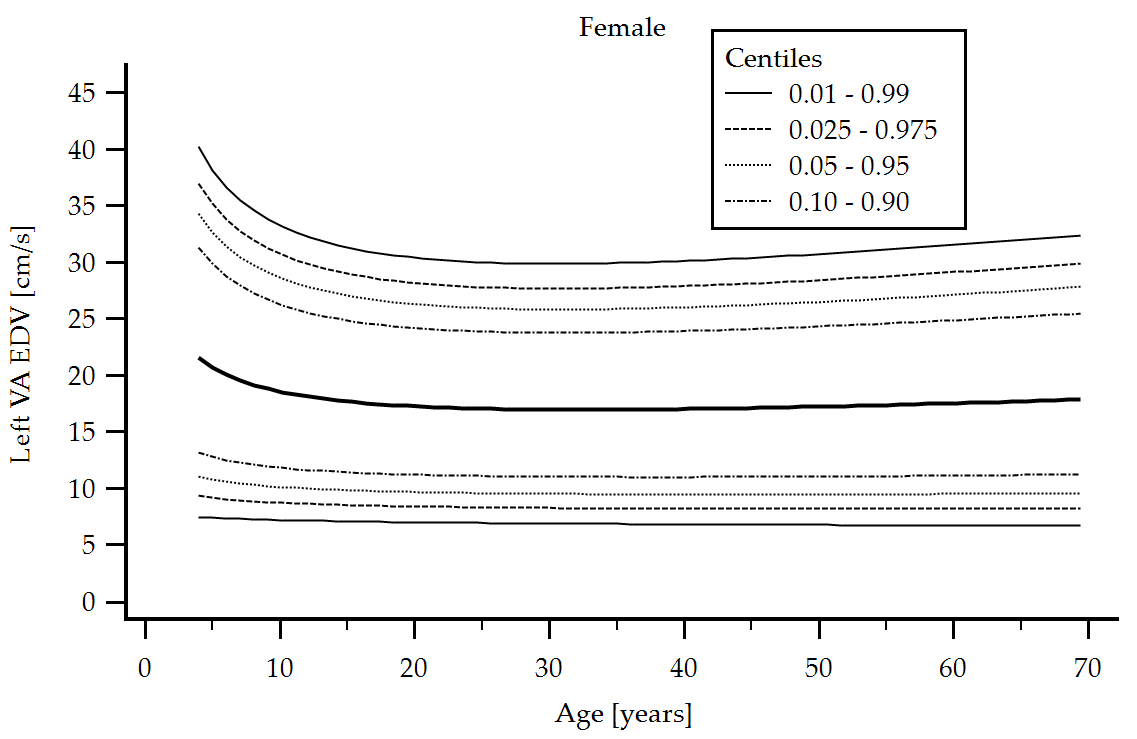

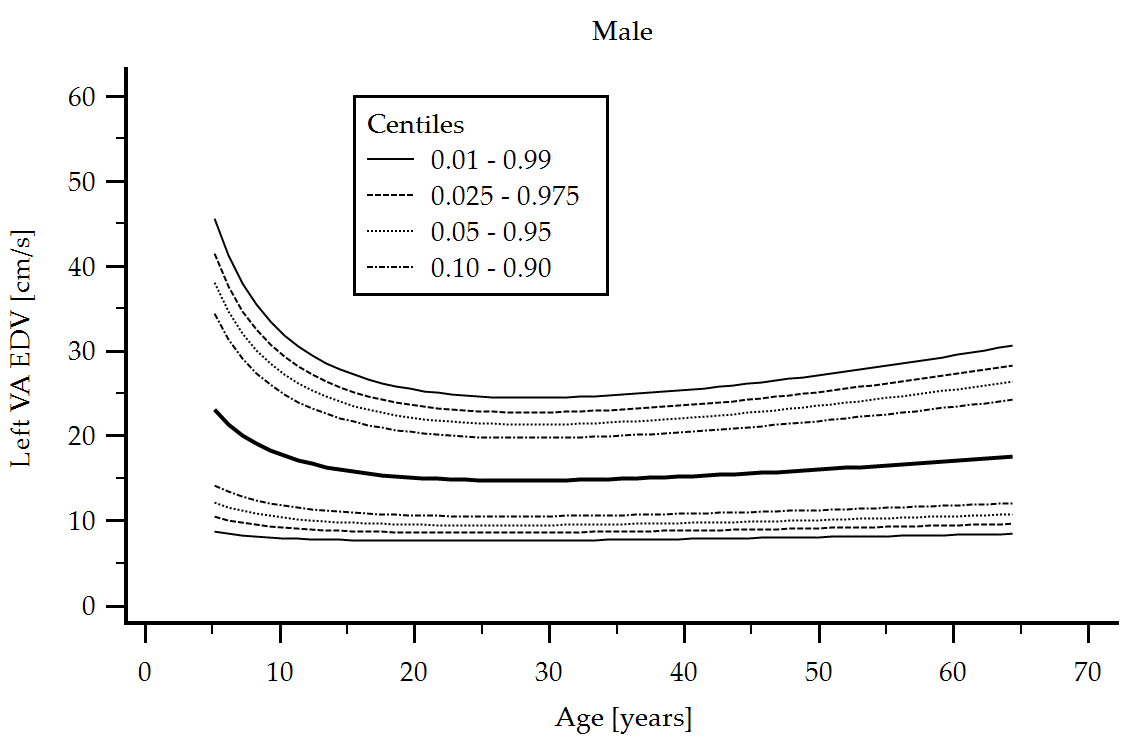


Supplementary Figure 26. Left Vertebral Artery (VA) blood flow velocity percentile curves for all, females and males. EDV: end-diastolic velocity.


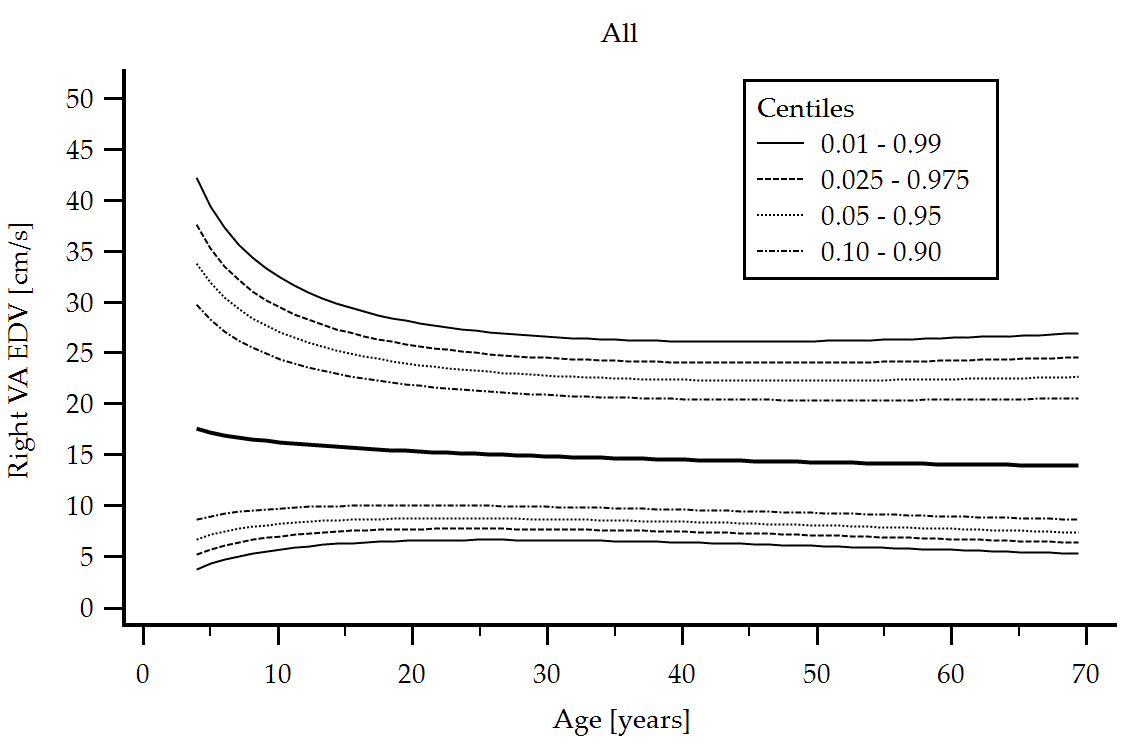

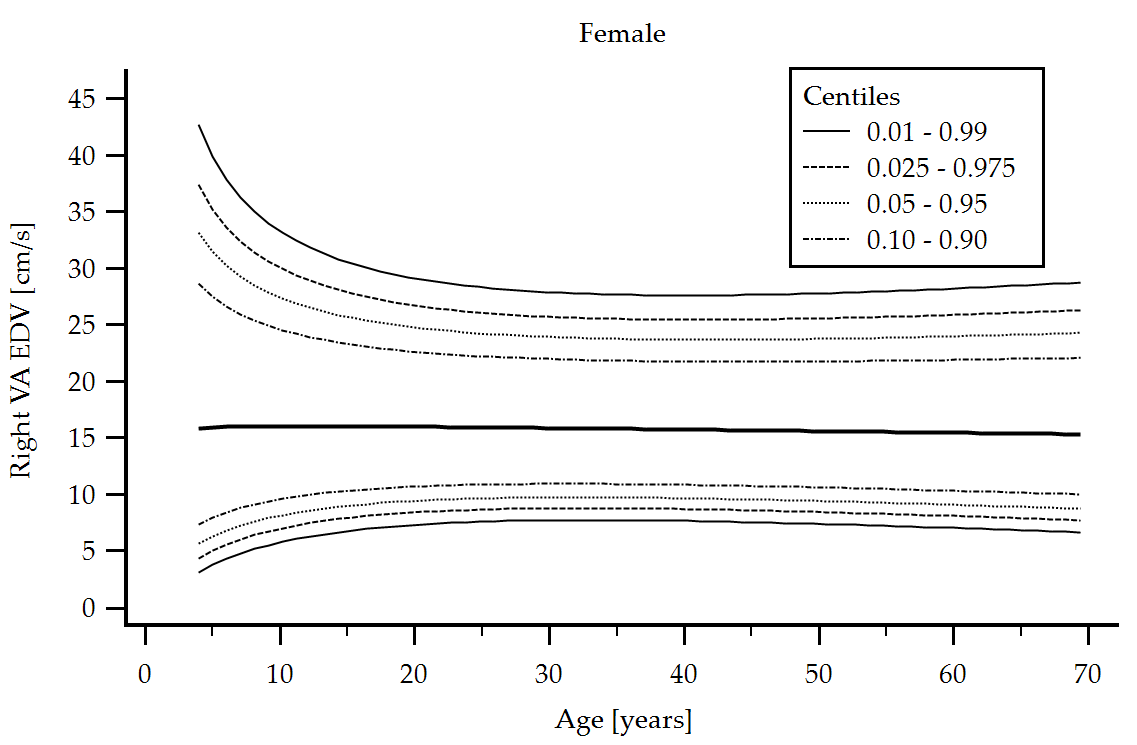

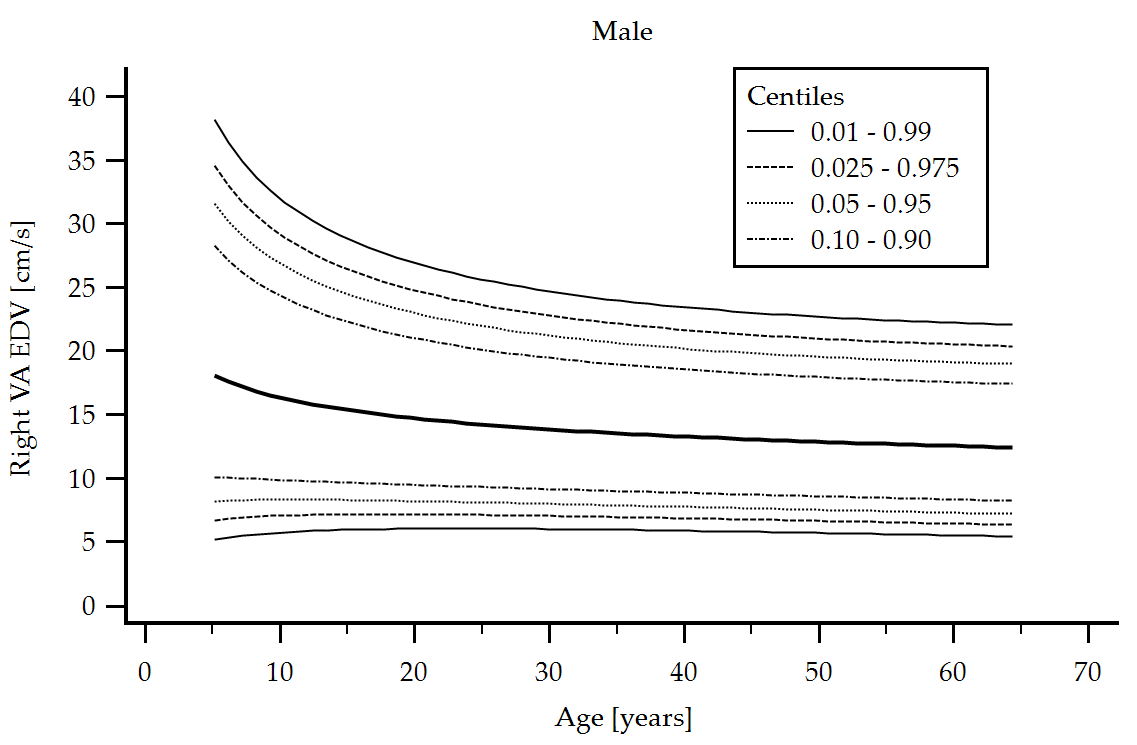


Supplementary Figure 27. Right Vertebral Artery (VA) blood flow velocity percentile curves for all, females and males. EDV: end-diastolic velocity.


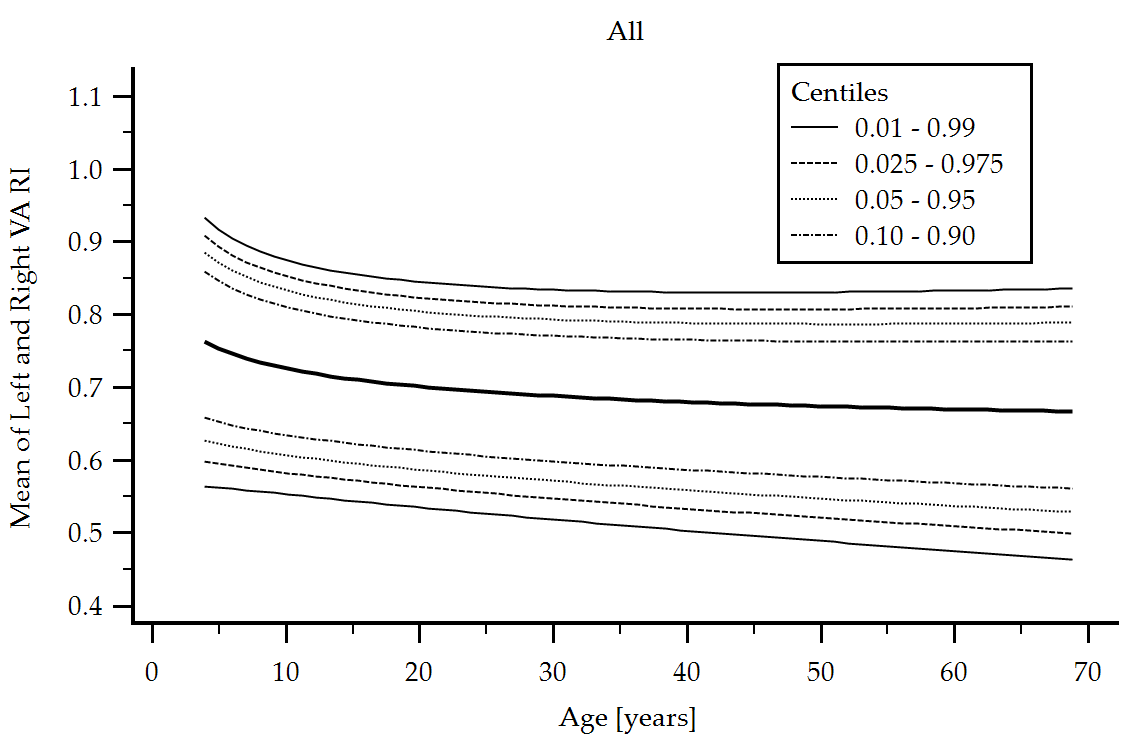

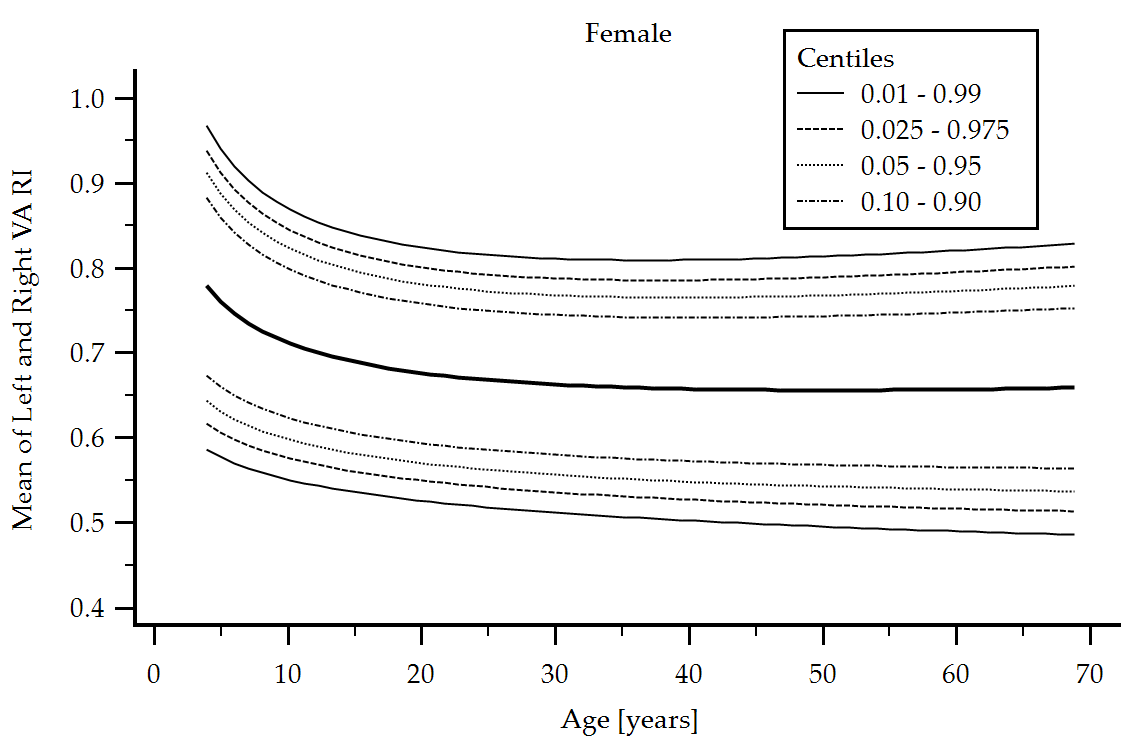

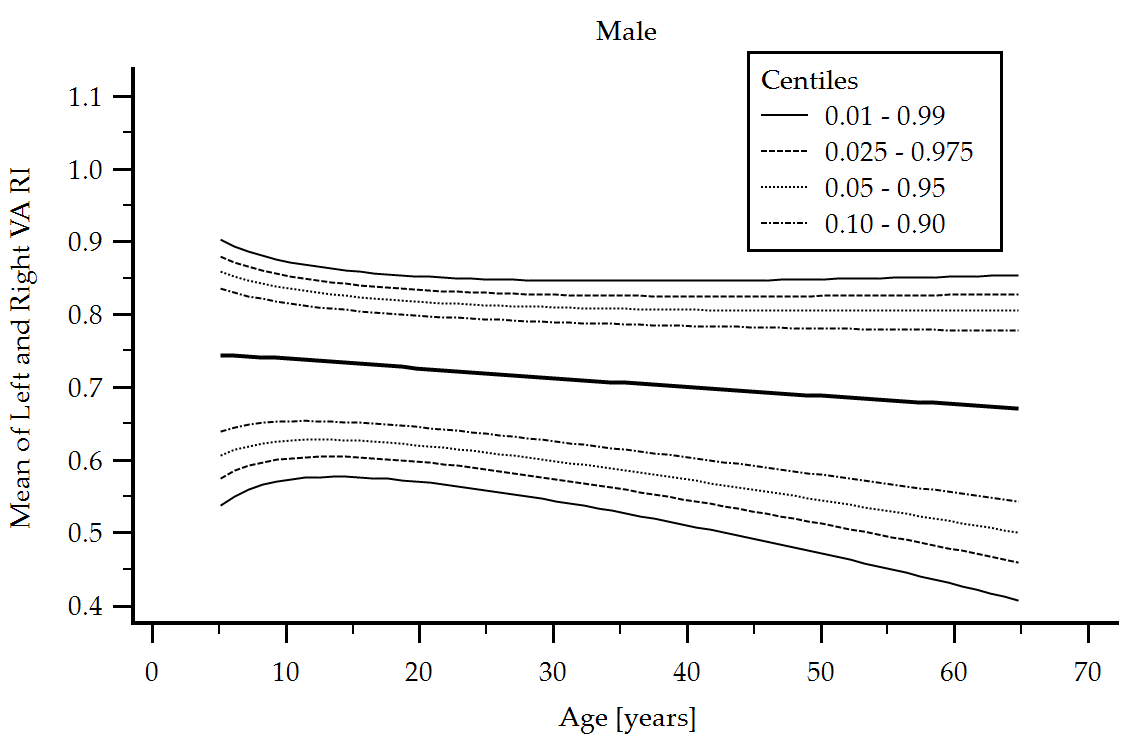


Supplementary Figure 28. Vertebral Artery (VA) blood flow velocity percentile curves for all, females and males. RI: resistive index..


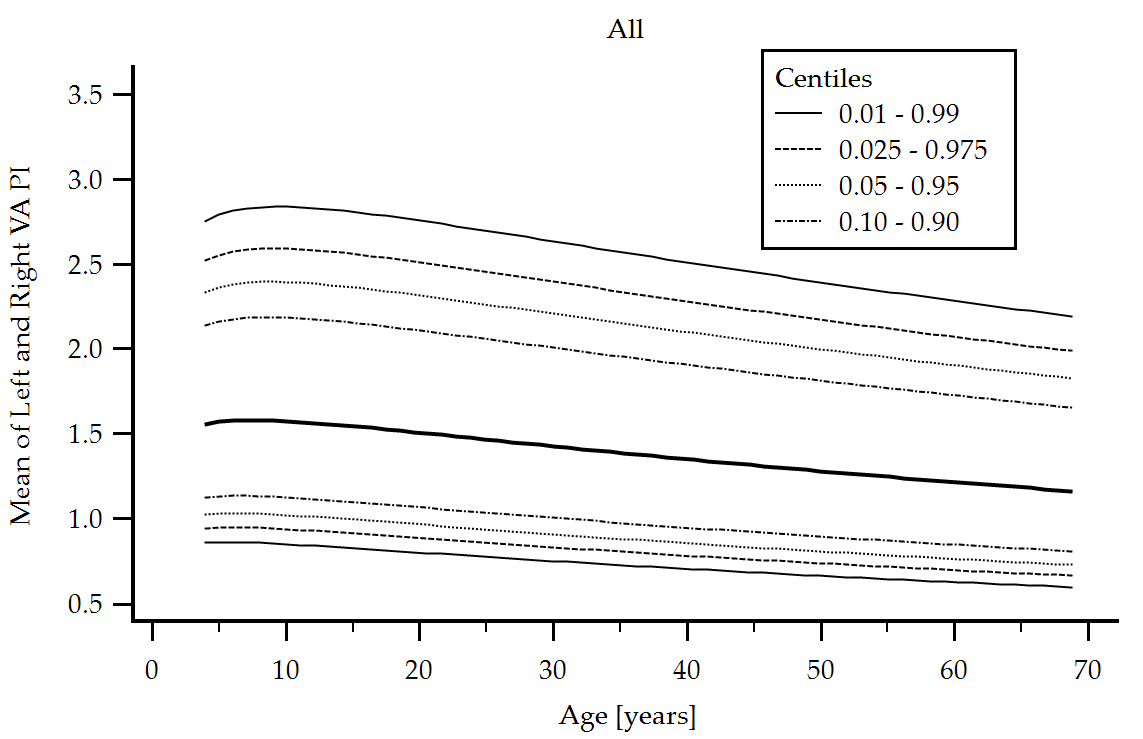

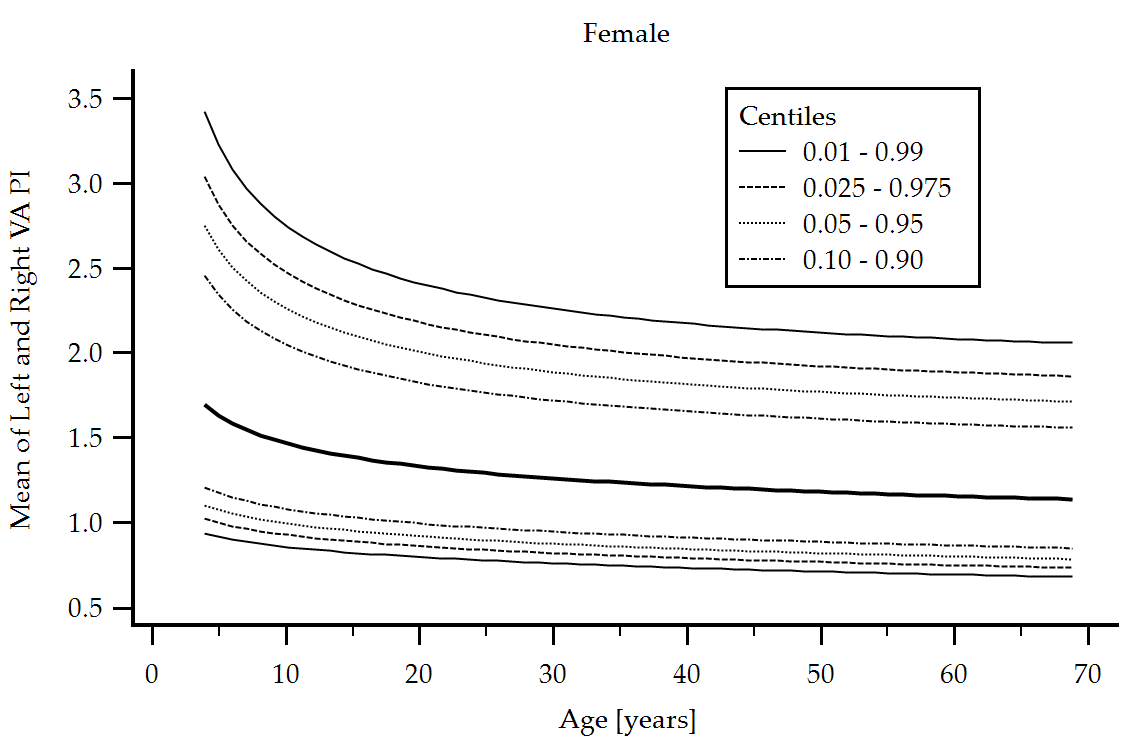

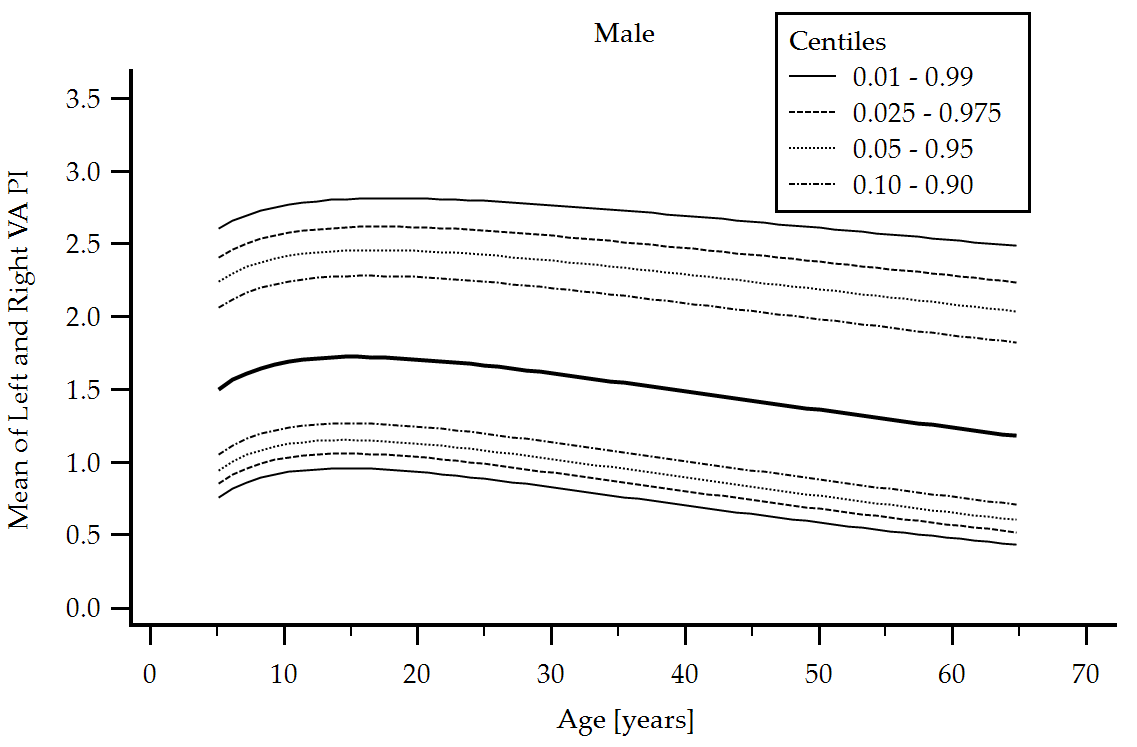


Supplementary Figure 29. Vertebral Artery (VA) blood flow velocity percentile curves for all, females and males. PI: pulsatile index..


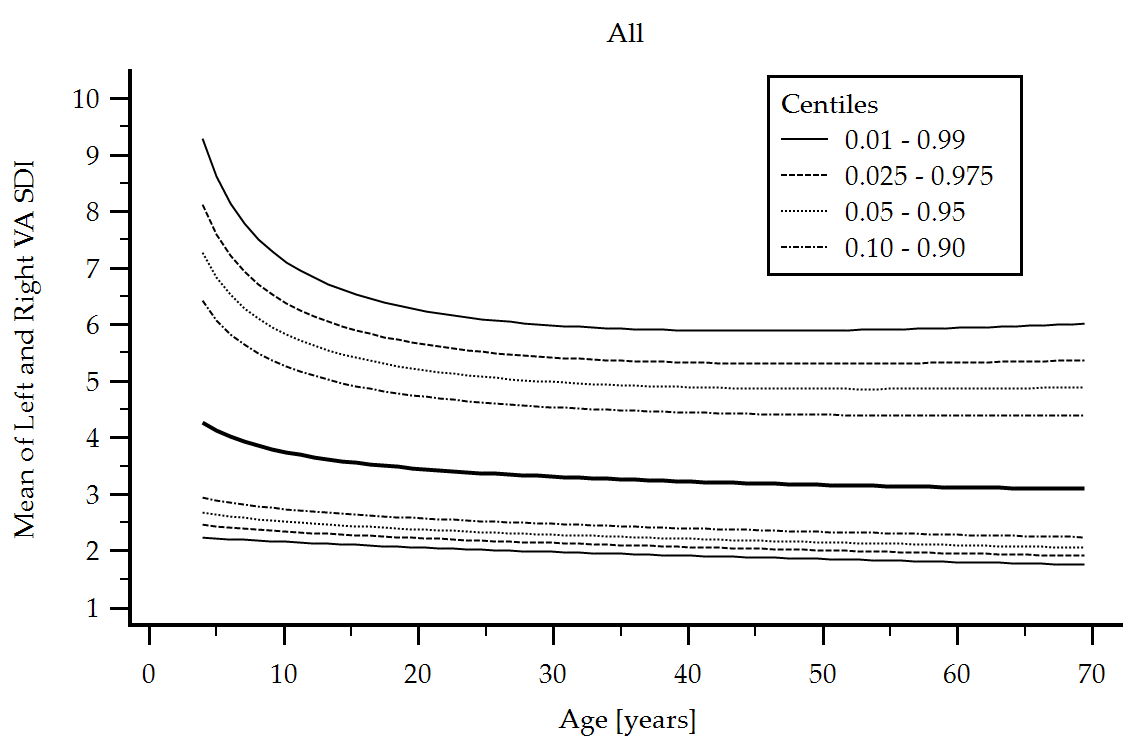

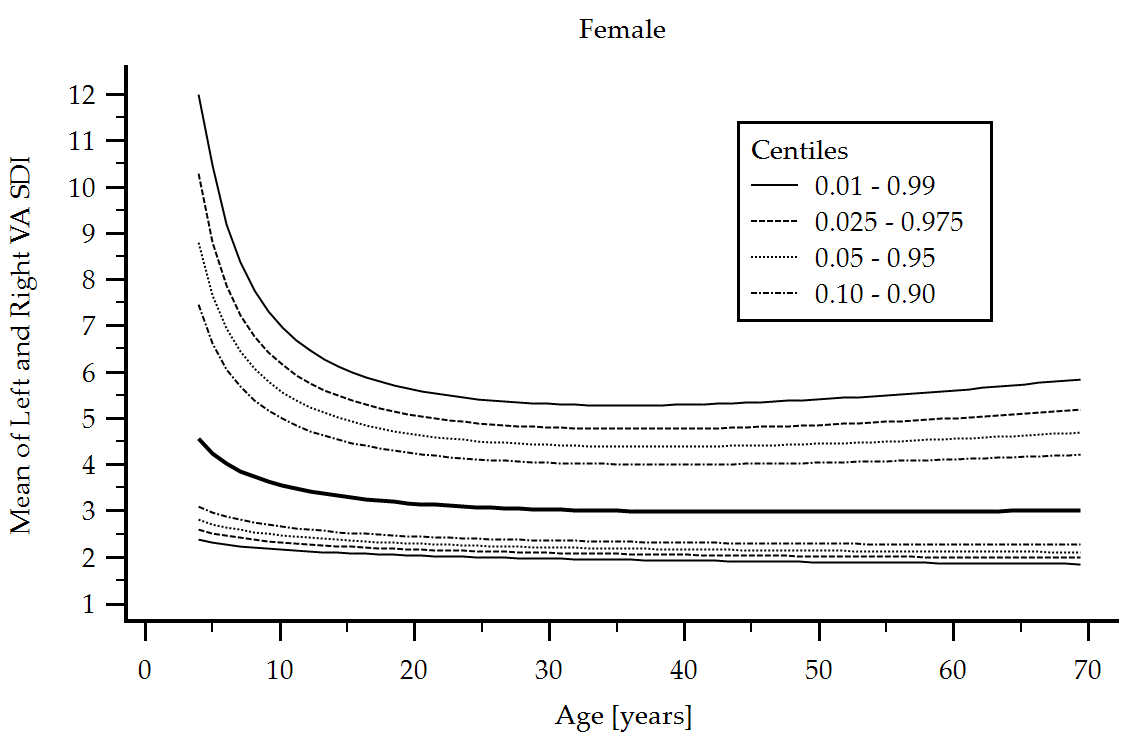

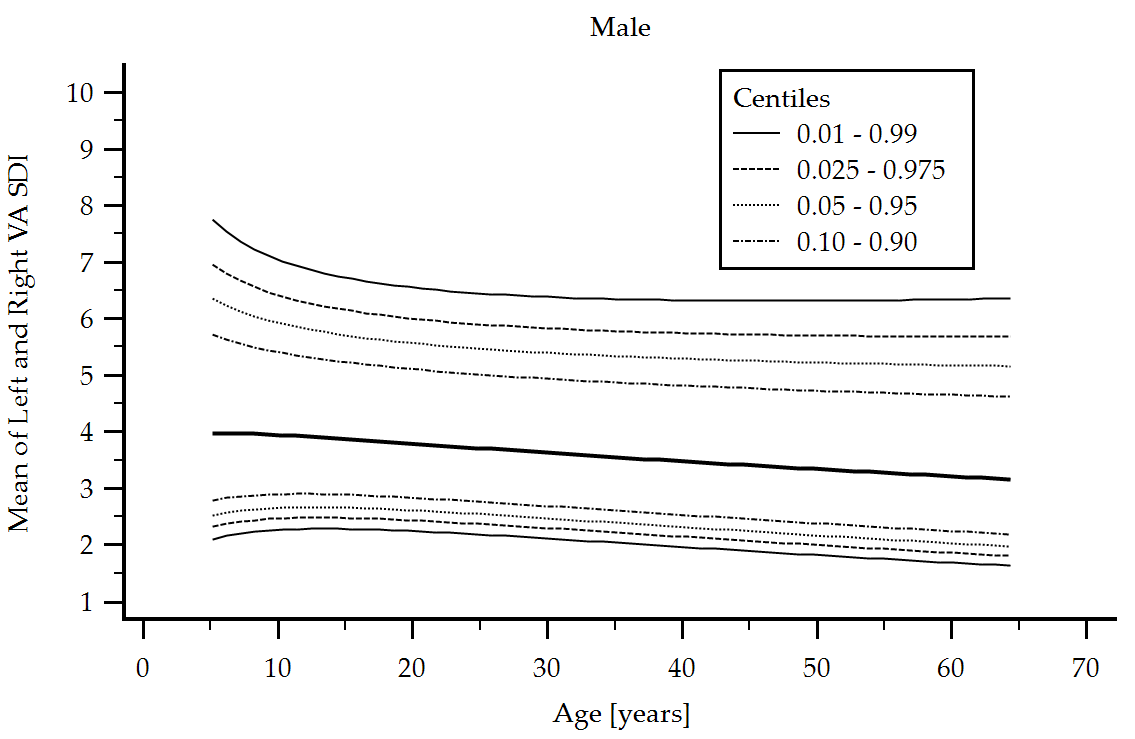


Supplementary Figure 30. Vertebral Artery (VA) blood flow velocity percentile curves for all, females and males. SDI: systo-diastolic index..

**Supplementary Figures: Common Femoral Artery (CFA)**

**
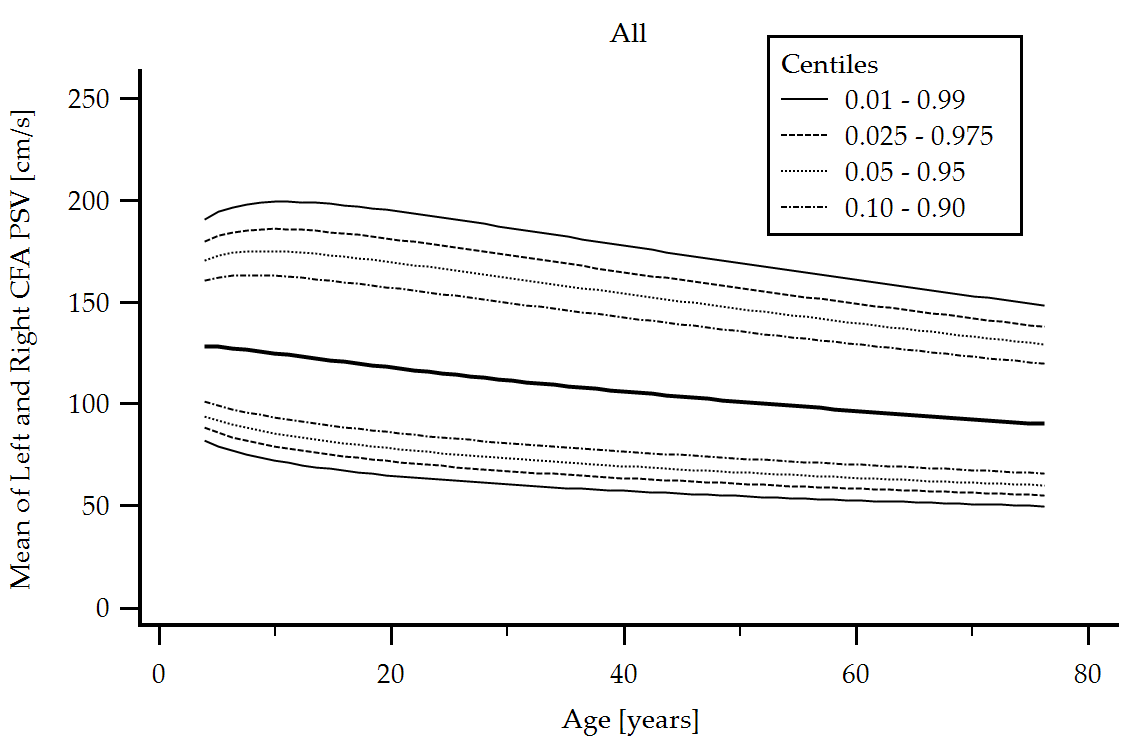

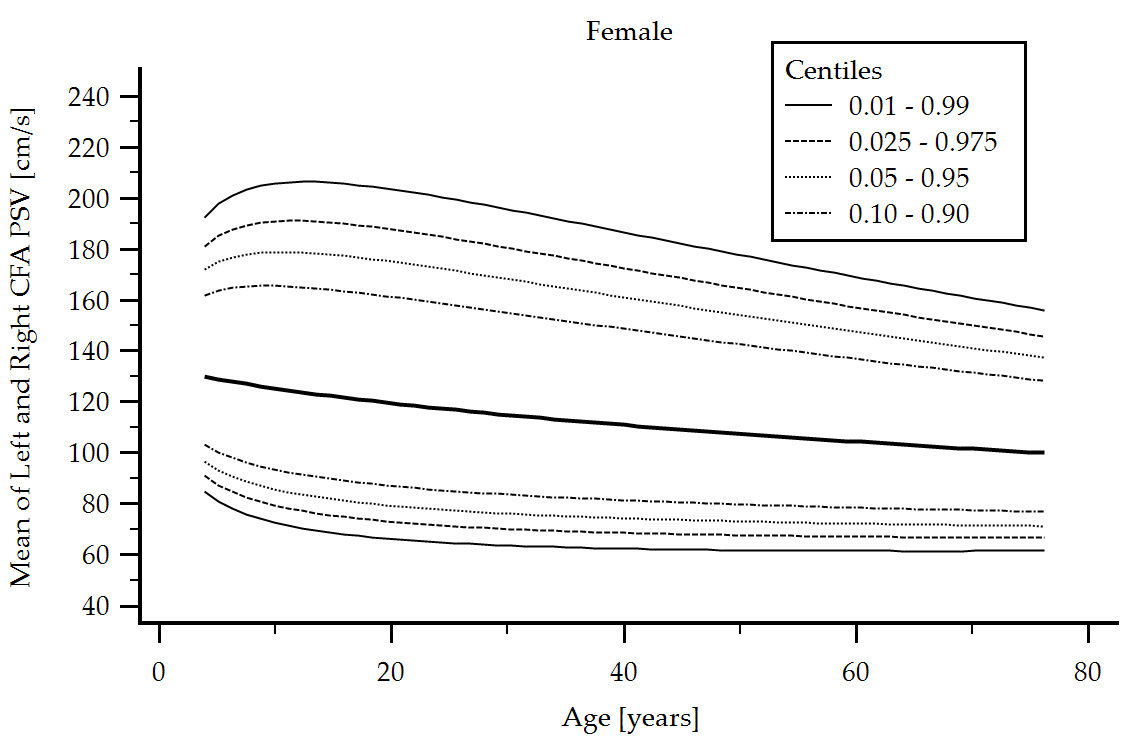

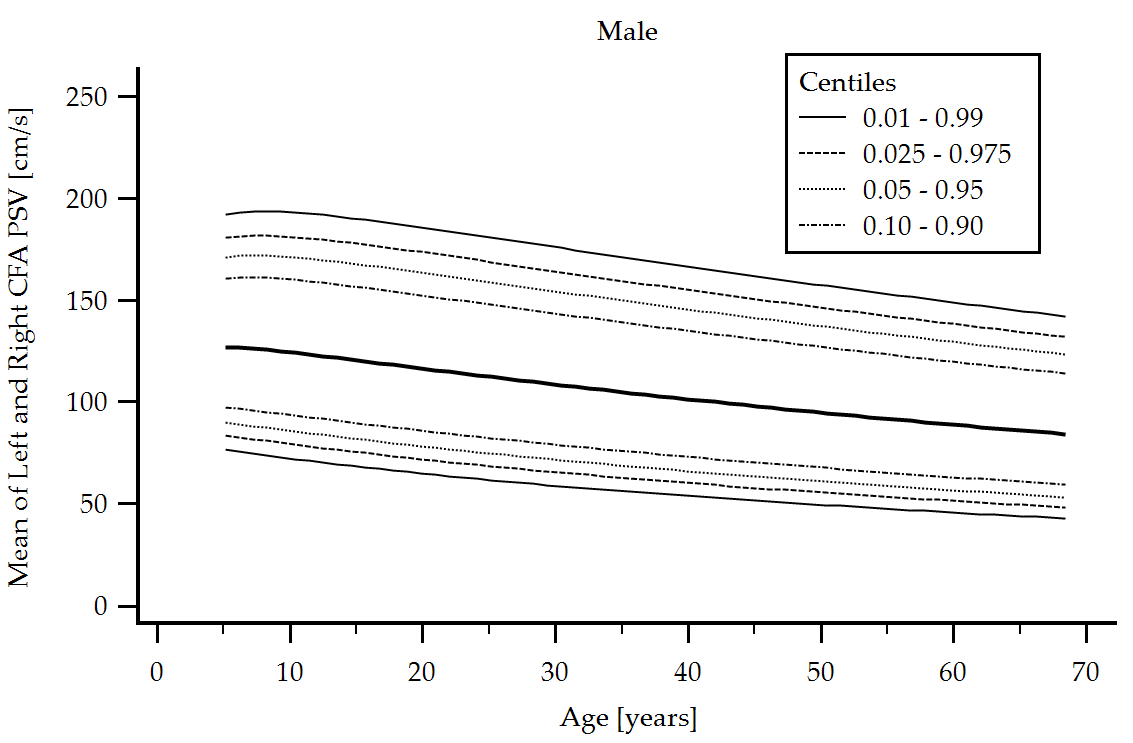
**

Supplementary Figure 31. Common Femoral Artery (CFA) blood flow velocity percentile curves for all, females and males. PSV: peak systolic velocity.


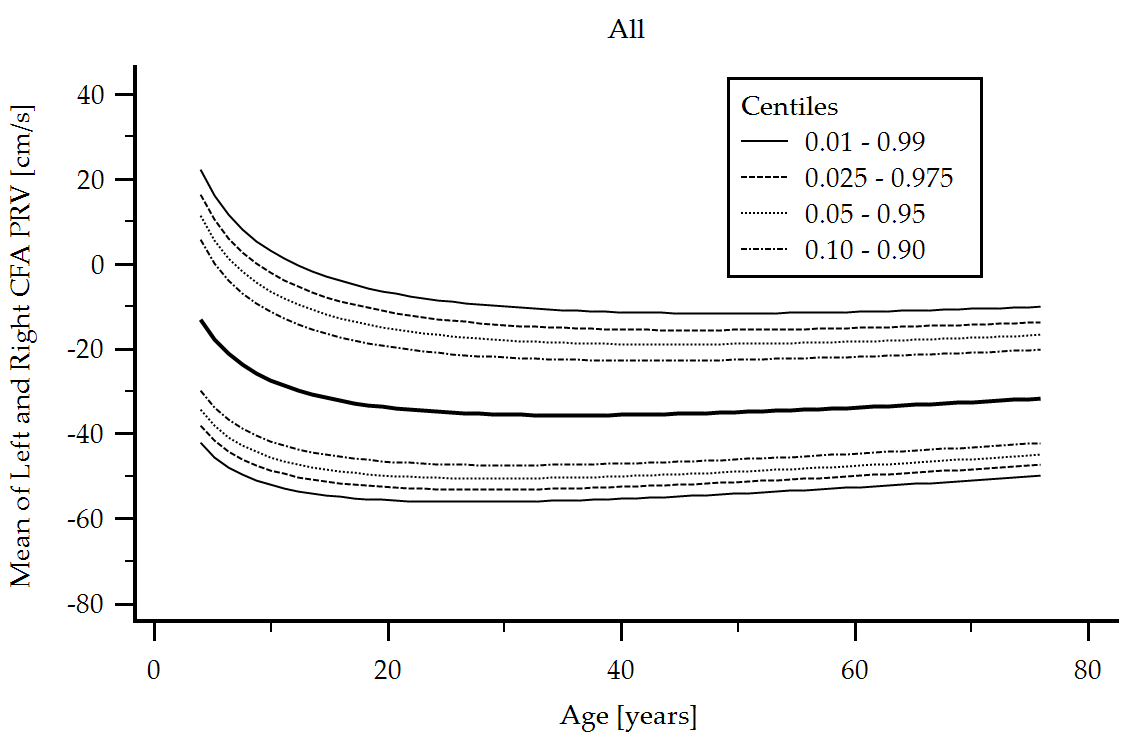


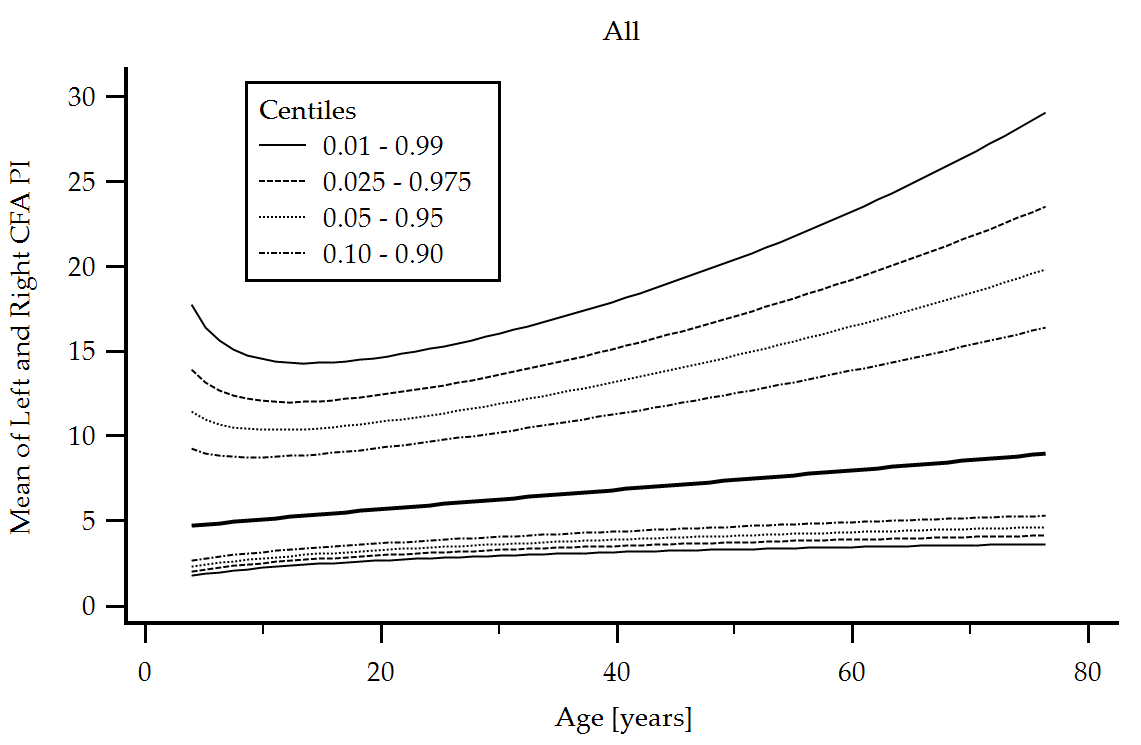


Supplementary Figure 32. Common Femoral Artery (CFA) blood flow velocity percentile curves. PRV: peak reversal velocity. PI: pulsatile index.

**Supplementary Figure: Brachial Artery (BA)**


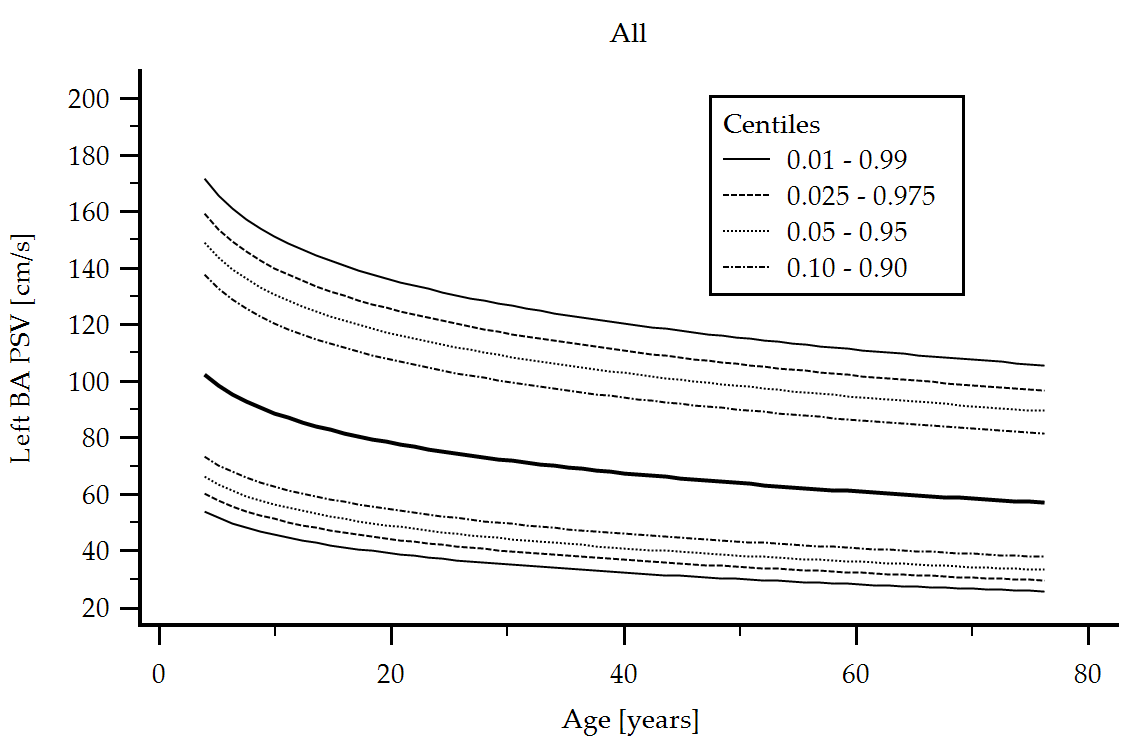


Supplementary Figure 33. Left Brachial Artery (BA) blood flow velocity percentile curves. PSV: peak systolic velocity.
